# Bilayer nanographene reveals halide permeation through a benzene hole

**DOI:** 10.1038/s41586-024-08299-8

**Published:** 2025-01-15

**Authors:** M. A. Niyas, Kazutaka Shoyama, Matthias Grüne, Frank Würthner

**Affiliations:** 1 Institut für Organische Chemie, Universität Würzburg, Würzburg, Germany; 2 Center for Nanosystems Chemistry (CNC), Universität Würzburg, Würzburg, Germany

**Keywords:** Chemistry, Materials science

## Abstract

Graphene is a single-layered *sp*^2^-hybridized carbon allotrope, which is impermeable to all atomic entities other than hydrogen^1,2^. The introduction of defects allows selective gas permeation^3–5^; efforts have been made to control the size of these defects for higher selectivity^6–9^. Permeation of entities other than gases, such as ions^10,11^, is of fundamental scientific interest because of its potential application in desalination, detection and purification^12–16^. However, a precise experimental observation of halide permeation has so far remained unknown^11,15–18^. Here we show halide permeation through a single benzene-sized defect in a molecular nanographene. Using supramolecular principles of self-aggregation, we created a stable bilayer of the nanographene^19–23^. As the cavity in the bilayer nanographene could be accessed only by two angstrom-sized windows, any halide that gets trapped inside the cavity has to permeate through the single benzene hole. Our experiments reveal the permeability of fluoride, chloride and bromide through a single benzene hole, whereas iodide is impermeable. Evidence for high permeation of chloride across single-layer nanographene and selective halide binding in a bilayer nanographene provides promise for the use of single benzene defects in graphene for artificial halide receptors^24,25^, as filtration membranes^26^ and further to create multilayer artificial chloride channels.

## Abstract (web summary)

Halide permeation through a single benzene-sized defect in a molecular nanographene shows the permeability of fluoride, chloride and bromide, whereas iodide is impermeable.

## Main

Understanding the permeation of halides through a single benzene defect in graphene is of high importance in the development of halide battery materials^27,28^, the design of artificial anion receptors^24,25^ and the development of filtration membranes^26^. Theoretical investigation of chloride permeation through holes in graphene indicated a minimum pore size of about 5–7 Å below which the chloride ions are rejected^13,16,18^. As these calculations are done in a water medium, owing to its implication in desalination, the hydration shell around chloride ions limits the permeation. Thus, a clear scientific insight into pristine chloride permeation through a sub-nanometre defect remains unknown. Here we synthesized a nanographene with an atomically precise single benzene pore with a diameter of 1.4 Å (refs. ^19–23^). Experimental evidence for halide permeation was obtained by first forming a stable dimer (thermodynamically and kinetically) of such molecular nanographene with a single benzene-sized pore and subsequent formation of the halide-trapped bilayer nanographene. ^1^H NMR titration experiments, mass spectrometry and single-crystal X-ray crystallographic analyses showed unambiguous evidence for the formation of a three-component complex in which a halide ion is trapped in the bilayer complex. As the halide has only two angstrom-sized pores to reach the cavity, the experimental observation of the three-component supramolecular complex explicitly proves the permeation of halide across a single benzene defect.

## Design and synthesis of nanographene with a single benzene hole

**Fig. 1:**
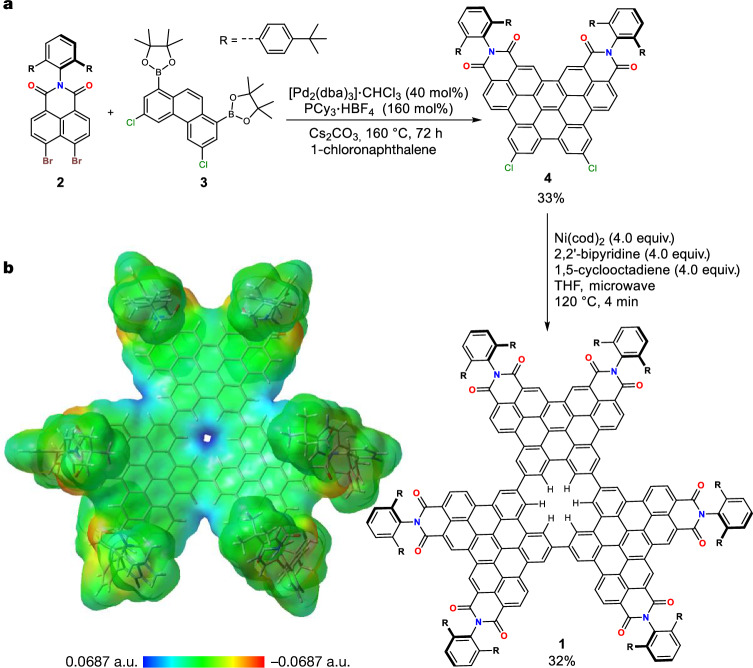
Synthesis of nanographene 1. **a**, Reaction scheme for the synthesis of **1**. **b**, Calculated ESP map of **1**. ESP map shows a positive electrostatic potential in the hole of macrocycle indicating the possibility of anion binding. [Pd_2_(dba)_3_]·CHCl_3_, tris(dibenzylideneacetone)dipalladium(0)–chloroform adduct; PCy_3_·HBF_4_, tricyclohexylphosphonium tetrafluoroborate; Ni(cod)_2_; bis(cyclooctadiene)nickel(0).

**Extended Data Fig. 1:**
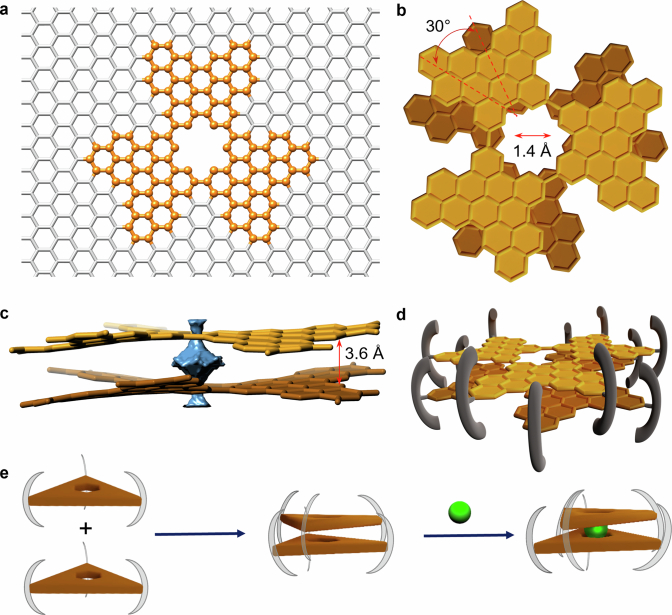
Design of kinetically stable holey bilayer nanographene to prove halide permeation through a single benzene defect. (a) Graphical representation of single benzene defected graphene highlighting the nano-segment that is synthesized in this work. (b) Molecular design of bilayer nanographene showing the twist and pore diameter (defined as the distance between the edges of the van der Waals spheres). (c) Side view of bilayer graphene showing the sub-nanometre channel as blue surface. (d) Design of stable bilayer nanographene showing the clamp-like side chains (in grey colour) that stabilize the dimer kinetically. (e) Schematic representation of dimerization and halide binding reported in this work. Halides permeate through the benzene defect in the kinetically stable dimer and bind inside the cavity.

Nanographene **1** is designed to have a single benzene hole and can form a kinetically stable supramolecular dimer (Extended Data Fig. 1). Monomer **1** consists of a *D*_3h_ symmetric graphene substructure containing a single benzene hole (Fig. 1 for chemical structure and Extended Data Fig. 1a for schematic image). The core structure of **1** consists of a cyclic trimer of phenanthrene, which forms a single benzene hole and six naphthalimide units at the periphery. When two monomers stack with each other with a rotation of about 30° they form a bilayer nanographene with an open channel having a diameter of 1.4 Å (Extended Data Fig. 1b). This angstrom-sized channel has a cavity in the middle, which is accessible from the openings on both sides (Extended Data Fig. 1c). A di-*tert*-butyl-*meta*-terphenyl group was attached at the imide nitrogen to allow nanographene to form a kinetically stable dimer (Extended Data Fig. 1d). We have in our previous work^29,30^ shown the formation of multilayer nanographenes by using the same substituent, which provides interlocking by C–H···π dispersion forces between the substituents and core π-scaffold. The assumed three-component complex would accommodate a halide anion placed at the centre of the bilayer complex of nanographene **1**, in which the halide anion is stabilized by forming a maximum of 12 C–H···X^–^ hydrogen bonds (X^–^ is any halide). These multiple C–H···X^–^ bonds have been successfully applied to realize record-high association constant for chloride binding to a host molecule^31^. With this design in hand, we proposed that an experimental observation of an anion trapped inside the channel would prove the permeation of chloride through the single benzene defect in a graphene (Extended Data Fig. 1e).

**Fig. 2:**
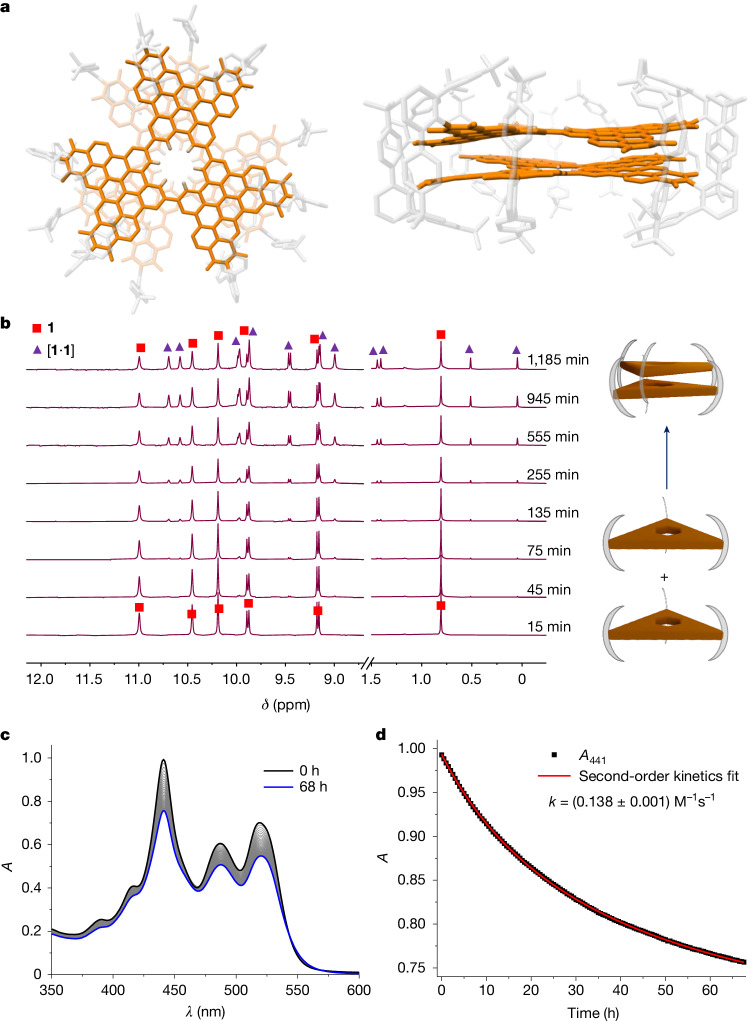
Crystal structure and solution stability of holey bilayer nanographene. **a**, Crystal structure of [**1**·**1**] showing holey bilayer nanographene in top view and side view. **b**, ^1^H NMR spectra of **1** in 1:1 Tol-*d*_*8*_:MeCN-*d*_*3*_ (*c*(**1**) = 2.2 × 10^–4^ M, 295 K, 400 MHz) showing the time-dependent dimerization of **1**. Splitting of multiple signals arising after 2 h in ^1^H NMR spectra correspond to the dimeric structure as seen in the crystal structure. **c**, Time-dependent UV–vis absorption spectra (*c*(**1**) = 5.5 × 10^–5^ M, 295 K, 1:1 Tol:MeCN) showing changes in the spectra with time indicating dimerization. **d**, Time-dependent absorbance for nanographene dimerization at 441 nm with second-order kinetics fit.

Nanographene **1** was synthesized by a recently developed palladium-catalysed cascade annulation reaction^30,32^ to prepare bisimide **4**, followed by a Yamamoto coupling of **4** under microwave conditions (Fig. 1 and Supplementary Information). Cyclotrimerization of π-extended phenanthrene **4** forms a single benzene hole in an otherwise fully π-conjugated nanographene. Nanographene **1** exists as monomers in solubilizing organic solvents (empirically these are solvents that contain multiple chloride substituents or are small aromatic molecules) such as CHCl_3_, CH_2_Cl_2_ and toluene (Tol) at room temperature. Monomeric mass in MALDI and six-fold symmetry in ^1^H NMR were observed in the samples prepared in the above-mentioned solvents, confirming the monomeric nature of **1** in these solvents. ^1^H NMR also confirmed the presence of monomers even at high concentrations (about 10^–3^ M^–1^) for a prolonged time (>2 weeks) in these solvents. These observations indicate that the monomer state is thermodynamically more stable for **1** in good solvating solvents. On the contrary, in solvents such as dimethyl sulfoxide (DMSO), dimethylformamide (DMF) or acetonitrile (MeCN) with higher cohesive energy density^33^, dimers are thermodynamically more stable than monomer of nanographene **1**. The expected bilayer complex of **1** could be observed in the crystal structure of **1** grown from DMSO/CHCl_3_/hexane solution (Fig. 2a). Each molecule of **1** was interlocked onto each other to form dimers in the crystalline state. Dimers are π–π stacked precisely on top of each other, thereby creating an angstrom-sized channel with a single benzene window. Energy decomposition analysis of the dimeric crystal structure computed using absolutely localized molecular orbitals—energy decomposition analysis (ALMO-EDA)^34^ method showed dispersion as the most stabilizing interaction followed by electrostatics (Supplementary Fig. 12 and Supplementary Table 7).

## Kinetic stability of bilayer nanographene

We then investigated the kinetics of dimer formation in solution using ultraviolet–visible (UV–vis) absorption and ^1^H NMR spectroscopy at 295 K. On addition of MeCN to a solution of **1** in Tol (1:0 to 1:4 Tol:MeCN, *c*(**1**) = 5.5 × 10^–5^ M), UV–vis absorption spectra showed time-dependent changes over several hours. Owing to the low solubility of **1** at high MeCN content, we performed all the following experiments at 1:1 Tol:MeCN mixture unless otherwise noted (detailed experimental procedure described in Supplementary Information). Time-dependent ^1^H NMR spectroscopy showed a new set of signals arising over 2 h (Fig. 2b). The splitting of the signals, especially the *tert*-butyl groups on **1**, matched the broken symmetry in the interlocked dimeric structure postulated in Extended Data Fig. 1c. Furthermore, MALDI mass spectrometry of samples prepared from this solvent mixture confirmed the mass of dimer indicating the high stability of dimers also in the gas phase. This time-dependent dimerization intrigued us to further investigate the thermodynamic and kinetic parameters of the dimerization process. The dimerization equilibrium constant, *K*_d_, was calculated to be (5.3 ± 0.5)·10^3^ M^–1^ in 1:1 Tol:MeCN at 295 K, corresponding to a Gibbs free energy release of –21.1 kJ mol^–1^. The second-order rate constant for the dimerization, *k*_d_, was calculated to be 0.14 M^−1^ s^−1^ in 1:1 Tol:MeCN at 295 K (Fig. 2c,d). An activation barrier of 85 kJ mol^–1^ was calculated by fitting the temperature-dependant rate constants (Supplementary Fig. 9). Such a slow kinetics of formation combined with the thermodynamic stability makes it a kinetically stable dimer with an activation energy of 106 kJ mol^–1^ for the dissociation of dimers into monomers.

It is currently impossible to visualize and study the permeation of ions through individual angstrom-sized pores in monolayer graphene by state-of-the-art techniques such as high-resolution transmission electron microscopy, atomic force microscopy or scanning tunnelling microscopy^8^. However, the precise overlap of the single benzene hole in the bilayer nanographene **1** allowed us to explore the possibility of dimers to trap anions thereby providing an unambiguous experimental observation of anions passing through an angstrom-sized hole in graphene. The single benzene hole in **1** is surrounded by six C–H hydrogens that are polarized because of six electron-withdrawing imide groups. The electrostatic surface potential (ESP) map of **1** (Fig. 1b) shows a positive potential at the hole, indicating the polarized nature of the C–H hydrogens at the centre. This polarization allows the C–H hydrogens to act as weak hydrogen bond donors^35^. Although individual C–H⋯X^–^ hydrogen bonds are weak, we proposed that the 12 C–H hydrogens in dimer [**1**·**1**] can strongly bind anions such as halides through multiple C–H⋯X^–^ hydrogen bonds. Notably, anion binding in sandwich-like complexes was reported previously^36,37^, but owing to the thermodynamic equilibrium between 1:1 and 2:1 host:anion species, guest anion influenced the dimerization and thus did not allow the investigation of anion permeation. Here, as we can specifically form kinetically stable dimers (in selected solvents), it is possible to assess anion binding in dimers directly.

## Halides permeation through a benzene hole

**Fig. 3:**
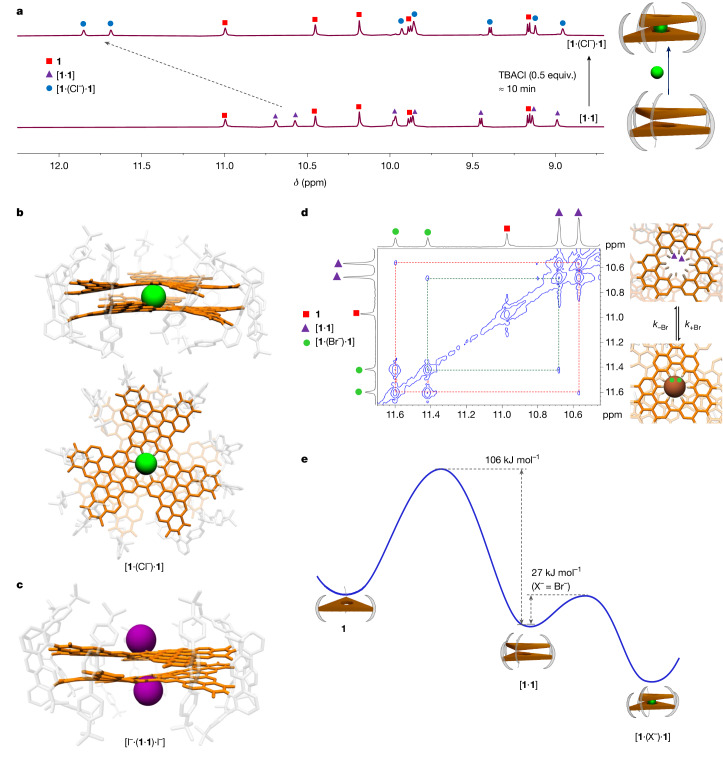
Analysis of halide permeation through a single benzene defect in the bilayer nanographene. **a**, ^1^H NMR spectra of **1** and TBACl (*c*(**1**) = 1.5 × 10^–4^ M, 295 K, 600 MHz) in 1:1 Tol-*d*_*8*_-MeCN-*d*_*3*_ showing the formation of chloride-bound dimers ([**1**·(Cl^–^)·**1**]). All [**1**·**1**] dimers are quickly converted to [**1**·(Cl^–^)·**1**], indicating the faster chloride binding kinetics compared with dimerization. **b**, Crystal structure of [**1**·(Cl^–^)·**1**] showing chloride-bound dimers in side view and top view. Chloride sits at the centre of the cavity formed by 12 central C–H hydrogens. **c**, Crystal structure of [I^–^·(**1**·**1**)·I^–^] showing the blocking of holes in the dimer by iodide. **d**, Excerpt from 2D EXSY spectra of bromide binding taken at 323 K with a mixing time *τ*_m_ of 200 ms (1:1 Tol-*d*_*8*_:MeCN-*d*_*3*_, 600 MHz, *c*(**1**) = 1.5 × 10^–4^ M, *c*(TBABr) = 1.5 × 10^–4^ M) showing separate signals for **1**, [**1**·**1**] and [**1**·(Br^–^)·**1**]. Cross signals between protons in the single benzene holes of [**1**·**1**] and [**1**·(Br^–^)·**1**] correspond to chemical exchange between the two species. **e**, Graphical representation of the energy landscape showing the energy barrier to form dimers from monomers (2(**1**) → [**1**·**1**]) and the barrier for halide binding by dimers ([**1**·**1**] + X^–^ → [**1**·(X^–^)·**1**]).

We first chose chloride as the anion to understand the permeation of chloride across single-benzene-defected graphene. Because dissociation of [**1**·**1**] into 2(**1**) should be much slower than the dimerization event (discussed above), a substantially faster binding of Cl^–^ by [**1**·**1**] than dimerization of **1** into [**1**·**1**] should corroborate chloride permeation through a single benzene hole of [**1**·**1**]. We first created [**1**·**1**] by dissolving **1** in 1:1 Tol:MeCN and incubating for 20 h at 295 K (*c*([**1**]) = 1.5 × 10^–4^ M). Then we added tetra-*n*-butylammonium chloride (TBACl, 0.5 equiv. to **1**) to the dimer solution and found that [**1**·**1**] was quickly (within first ^1^H NMR measurement time (about 10 min)) converted to [**1**·(Cl^–^)·**1**] (Fig. 3a). This binding rate ([**1**·**1**] + Cl^–^ → [**1**·(Cl^–^)·**1**]) is much faster compared with dimerization (2(**1**) → [**1**·**1**], a half-life of 13.4 h at 295 K for 1.5×10^–4^ M concentration of **1**) (Fig. 3) indicating that chloride binds directly to dimer by passing through the single benzene hole. The addition of chloride to a monomer solution to form dimer (2(**1**) + Cl^–^ → [**1**·(Cl^–^)·**1**]) took a similar duration as the dimerization of pure monomer (2(**1**) → [**1**·**1**]), which rules out the possibility of monomer intermediate ([**1**·(Cl^–^)]) involved in the fast complexation process ([**1**·**1**] + Cl^–^ → [**1**·(Cl^–^)·**1**]) (see Supplementary Information for details and several alternate mechanisms we considered). A direct determination of the binding constant for chloride by [**1**·**1**] complex proved challenging as even with the minimum amount of 0.5 equiv. of TBACl (*c*(Cl^–^) = 0.5 × *c*(**1**)), all the [**1**·**1**] were converted to [**1**·(Cl^–^)·**1**] indicating an ultrahigh affinity of chlorides to dimeric [**1**·**1**] that could not be directly evaluated by ^1^H NMR titration. This was not the case when we did the same experiment with bromide (as tetra-*n*-butylammonium bromide, TBABr). Each species ([**1**·**1**] and [**1**·(Br^–^)·**1**]) were observed individually even at an excess of 3 equiv. of TBABr (Supplementary Fig. 4). Thus, a binding constant of 1.2 × 10^3^ M^–1^ was determined for bromide binding (Table 1).

**Table 1:** Halide-binding constants of [1·1] at 295 K in Tol:MeCN (1:1)

Complex	*K*_halide_ (M^−1^)	Δ*G*_halide_ (kJ mol^−1^)
[**1**·(F^–^)·**1**]^a^	(6.8 ± 1.5)·10^7^	–44.2
[**1**·(Cl^–^)·**1**]^b^	(1.0 ± 0.3)·10^7^	–39.6
[**1**·(Br^–^)·**1**]^c^	(1.2 ± 0.4)·10^3^	–17.0
[**1**·(I^–^)·**1**]	No binding	–

^a^Association constant *K*_F–_ determined from a competitive binding ^1^H NMR experiment between F^–^ and Cl^–^. ^b^Association constant *K*_Cl–_ determined from a competitive binding ^1^H NMR experiment between Br^–^ and Cl^–^. ^c^Association constant *K*_Br–_ determined from equilibrium concentrations obtained directly from ^1^H NMR experiment. For all binding experiments, halide was added after dimerization for about 24 h and measured ^1^H NMR spectra within 1 h. The error in *K*_halide_ was obtained from triplicate experiments. Gibbs free energies Δ*G*_halide_ (295 K) are calculated from *K*_halide_ according to Δ*G*_halide_ = −*RT* ln(*K*_halide_). The experimental details are given in Supplementary Information.

Bromide binding allowed us to evaluate the affinity of chloride by a competitive binding experiment. Thus, an addition of around 750 equiv. of TBABr into a solution containing 0.5 equiv. TBACl and [**1**·**1**] showed separate signals for [**1**·(Br^–^)·**1**] and [**1**·(Cl^–^)·**1**] allowing us to determine the equilibrium concentration of each species (Supplementary Fig. 5). A similar competition experiment with chloride anion was used to obtain the binding affinity of fluoride to [**1**·**1**] (Supplementary Fig. 6). From the binding experiments, fluoride showed the highest binding affinity (*K*_F–_ = 6.8 × 10^7^ M^–1^) followed by chloride (*K*_Cl–_ = 1.0 × 10^7^ M^–1^) and bromide (*K*_Br–_ = 1.2 × 10^3^ M^–1^). Furthermore, MALDI showed masses of [**1**·(Cl^–^)·**1**] supporting the high stability of the supramolecular complex. For the chloride-bound dimer ([**1**·(Cl^–^)·**1**]), we presumed the position of chloride at the centre of bilayer nanographene from the ^1^H NMR proton signals shifts (only central C–H protons shift as the chloride binds) and symmetry (*D*_3_). An unambiguous proof for the chloride binding in the cavity created by the single benzene hole in the bilayer nanographene was finally provided by X-ray crystal structure analysis of [**1**·(Cl^–^)·**1**] (Fig. 3b). The crystal structure showed that chloride sits at the centre of the cavity created by single benzene hole defected bilayer nanographene. Chloride anion makes 12 C–H···Cl^–^ hydrogen bonds (*d*_H–Cl_ = 2.5–2.7 Å) in the cavity. The proposed interaction of chloride through multiple hydrogen bonding could also be corroborated by an additional partially occupied chloride ion found at one of the peripheral bay positions (Supplementary Fig. 3). Crystallographic analyses also identified a solvent-accessible void between two neighbouring dimers, which is occupied by the countercation (for crystal structure analysis we used tetraphenylphosphonium, (TPP)^+^) with a high degree of positional disorder (Supplementary Fig. 3, additional discussion in the Methods). The interlocking structure seen in the crystal structure of [**1**·**1**] is also preserved in the crystal structure of [**1**·(Cl^–^)·**1**]. Iodide showed no binding in the cavity even at large excess and thus proved to be too large for the permeation through the single benzene hole (Supplementary Fig. 7). However, a shift in ^1^H NMR signals at the hole indicated a binding outside the hole to form the complex of the type [I^–^·(**1**·**1**)·I^–^]. This was further confirmed by obtaining a co-crystal structure of iodide with **1** (Fig. 3c, Supplementary Table 4 and Supplementary Fig. 3). In the crystal structure of [I^–^·(**1**·**1**)·I^–^], two partially occupied iodides were found outside the hole in a way to block the hole. The structure further showed that the large size of iodide makes it impossible to pass through the single benzene hole.

To obtain further evidence for the dynamic process of halide permeation into bilayer nanographene, we used 2D ^1^H–^1^H exchange spectroscopy (EXSY)^38^ to identify exchange cross signals between [**1**·**1**] and [**1**·(Br^–^)·**1**]. We chose the bromide complex for 2D EXSY as the binding affinity is in the optimal range to see separate signals for [**1**·**1**] and [**1**·(Br^–^)·**1**] in ^1^H NMR spectra (for fluoride and chloride, even with 0.5 equiv. to **1**, all [**1**·**1**] are converted to [**1**·(F^–^/Cl^–^)·**1**]). At 323 K, exchange signals between [**1**·**1**] and [**1**·(Br^–^)·**1**] were observed at a mixing time (*τ*_m_) of 200 ms showing a chemical exchange between the dimer and bromide-bound dimer (Fig. 3d, Supplementary Fig. 10). Exchange signals between protons in the single benzene holes of [**1**·**1**] and [**1**·(Br^–^)·**1**] provide direct evidence for the halide binding of bilayer nanographene through the single benzene defect. By full relaxation matrix analysis of the 2D EXSY signal amplitudes using the EXSYCALC software, a forward rate constant, *k*_+Br_, of 101 M^−1^s^−1^ and a reverse rate constant, *k*_–Br_, of 0.055 s^−1^ was obtained for bromide binding in [**1**·**1**] at 323 K. An activation energy of 27.4 kJ mol^–1^ for bromide binding ([**1**·**1**] + Br^–^ → [**1**·(Br^–^)·**1**]) was obtained from the rate constants evaluated at different temperatures (Fig. 3e, Supplementary Table 6 and Supplementary Fig. 11). We also performed further experiments and theoretical analysis to evaluate possible alternate mechanisms that could explain the halide-binding observations (Supplementary Information section 8). Thus, apart from discarding alternate mechanisms, ^1^H NMR, MALDI and X-ray crystal structure proofs combined with the exchange signals and permeation kinetics obtained from 2D EXSY conclusively supported the mechanistic pathway of the passage of halides (F^–^, Cl^–^ and Br^–^) through a single benzene hole and finally getting trapped in the cavity of the angstrom-sized channel. We further performed computational investigations to estimate the energy barrier and the transition state associated with the chloride passage through a single benzene hole. We used the nudged elastic band—climbing image (NEB-CI)^39,40^ method to estimate the barrier to cross the single benzene hole with a semi-empirical GFN2-xTB^41^ level of theory. Halide permeation trajectory obtained from NEB-CI showed that fluoride permeation is virtually barrier-free, whereas chloride and bromide have significant barriers (Supplementary Figs. 13–16). At the transition state, halide is found at the centre of the single benzene hole, whereas the bilayer nanographene is slightly distorted. On the basis of these results, we can suggest the energy landscape for the subsequent processes of nanographene dimerization and halide ion complexation as shown in Fig. 3e.

## Conclusion

We showed halide permeation through an angstrom-sized single benzene hole in a nanographene. In the first step, we used strong dispersion interaction between nanographenes to construct a noncovalent π–π stacked dimer of nanographene. X-ray crystal structure, ^1^H NMR, MALDI and time-dependent UV–vis conclusively confirmed the structure and thermodynamic and kinetic stability of the holey bilayer nanographene complex in which single benzene holes in the dimers overlap to create a sub-nanometre channel with an angstrom-sized cavity surrounded by 12 polarized C–H hydrogens. Next, we showed the permeation of halides through the single benzene defect by trapping the halide in the cavity created by the holey bilayer nanographene. X-ray crystal structure of chloride-bound bilayer nanographene unambiguously showed the chloride at the centre of the angstrom-sized cavity of the holey bilayer nanographene. The 2D EXSY measurements provided direct evidence for the chemical exchange between halide-bound dimer and free dimer. Experimental binding affinities showed a micromolar affinity for fluoride and chloride, whereas even bigger bromide shows millimolar affinity. Iodide is found to be impermeable through the single benzene hole in the bilayer nanographene. Theoretical investigations of permeation barrier showed that fluoride binding is barrier-free, whereas chloride and bromide binding has a notable barrier to permeate. Although the investigation of the chemical permeability across atomically precise angstrom-sized defects in macroscopic graphene layers was extremely challenging with modern microscopy techniques, here we could prove the halide permeation across atomically precise angstrom-sized defects using the chemical synthesis of a structurally defined nanographene and its supramolecular dimer complex. We anticipate that this supramolecular strategy will be widely adopted to study the permeation of ionic or molecular entities through atomically precise defects in graphene substructures, or ultimately graphene itself.

## Methods

### Synthesis of **1**

Dichlorinated bisimide (**4**, 20 mg, 0.15 mmol, 1.0 equiv.), 2,2′-bipyridine (9.5 mg, 61 µmol, 4.0 equiv.), 1,5-cyclooctadiene (7.4 µl, 61 µmol, 4.0 equiv.) and Ni(cod)_2_ (17 mg, 61 µmol, 4.0 equiv.) were dissolved in 3 ml dry and degassed THF solution in a 5 ml microwave reactor pressure tube. The reaction was stirred at 120 °C for 4 min in a microwave reactor. After the reaction was cooled to room temperature, THF was removed by a rotary evaporator. The crude product was purified by silica-gel column chromatography (elution solvent: 1:0 to 0:1 CH_2_Cl_2_:ethylacetate) and semi-preparative gel-permeation chromatography (GPC, in CHCl_3_). The product fractions obtained by GPC separation were dissolved in THF containing an excess of TBACl and layered with cyclohexane (THF:cyclohexane = 1:4) overnight to afford precipitation of pure chloride complexes ([**1**·(Cl^–^)·**1**] complexes are not soluble in low polar solvent mixtures). The precipitates were filtered and dissolved in CH_2_Cl_2_ and washed with water in an extraction funnel to remove TBACl. A final recrystallization from CH_2_Cl_2_:methanol (1:4) mixture yielded pure **1** as a red solid. Yield: 6 mg (32%).

### Crystallographic analysis

Single crystals of [**1**·**1**] suitable for X-ray diffraction could be grown by slow diffusion of *n*-hexane into a chloroform/DMSO solution of **1** (*c*(**1**) ≈ 2 × 10^–4^ M, 293 K) over a week. Single crystals of [**1**·(Cl^–^)·**1**] was grown by mixing **1** and tetraphenylphosphonium chloride (TPPCl) in 1:1 Tol:MeCN (*c*(**1**) ≈ 2 × 10^–4^ M, *c*(TPPCl) ≈ 3 × 10^–4^ M, 293 K) and slow diffusion of MeCN over a week. Single crystals of [I^–^·(**1**·**1)**·I^–^] were grown by mixing **1** and tetraphenylphosphonium iodide (TPPI) in 1:1 Tol:MeCN (*c*(**1**) ≈ 3 × 10^–4^ M, *c*(TPPI) ≈ 3 × 10^–3^ M, 293 K) and slow diffusion of MeCN over a week.

Single-crystal X-ray crystallography was performed on a Bruker D8 Quest Diffractometer with a PhotonII detector using Cu Kα radiation for [**1**·**1**] or the P11 beamline at DESY for [**1**·(Cl^–^)·**1**] and [I^–^·(**1**·**1)**·I^–^]. The structures were solved using SHELXT^42^, expanded with Fourier techniques and refined using the SHELXL software package^43^. Hydrogen atoms were assigned at idealized positions and were included in the calculation of structure factors. All non-hydrogen atoms in the major disorder part of the main residues were refined anisotropically. In all crystal structures, some of the *N*-substituents (bis(*tert*-butyl)-*meta*-terphenyl groups) and solvent molecules were disordered and modelled with constraints and restraints using standard SHELX commands EADP, DFIX, FLAT, SAME, SADI, DELU, SIMU, CHIV, ISOR and RIGU.

The amount of chloride found in [**1**·(Cl^–^)·**1**] summed up to 1.40 equiv., comprising a fully occupied (1.00 equivalent) chloride ion in the cavity of dimer (**1**·**1**) and a partially occupied chloride ion at the periphery (Supplementary Fig. 3, 0.40 equiv.). In the crystal structure of [I^–^·(**1**·**1**)·I^–^], the iodide ions placed on top of both the holes of the dimer (**1**·**1**) are only partially occupied with occupancies of 0.191 and 0.142, respectively. The iodide at the periphery (Supplementary Fig. 3) had an occupancy of 0.072. These numbers account for the equivalents of respective iodide for each dimer (**1**·**1**).

For the refinement of [**1**·(Cl^–^)·**1**] and [I^–^·(**1**·**1**)·I^–^], the disordered countercation (TPP)^+^ residues were modelled either by rigid group constraints of the whole molecule using the FRAG command of SHELXL or by a combination of the phenyl ring constrain (AFIX 66 command) and distance restraints (DFIX and DANG commands). For [**1**·(Cl^–^)·**1**], the geometry of (TPP)^+^ optimized by density functional theory (DFT) calculations at the B3LYP/6-311 + G(d,p) level of theory was used. For [I^–^·(**1**·**1**)·I^–^], the four phenyl rings of (TPP)^+^ are constrained by AFIX 66 command of SHELXL and distances between P and phenyl rings were restrained by DFIX and DANG commands. The common plane restraints using the FLAT command of SHELXL was also applied to stabilize the connection between P and phenyl rings as well. The rigid group constraints were also applied for heavily disordered acetonitrile molecules using the geometry obtained by the DFT PBE1PBE/6-311++G(3df,3pd) level of theory^44^. The analysis of solvent-accessible voids identified a volume of about 1.4 nm^3^ (around 340 e^–^) for [**1**·(Cl^–^)·**1**] and about 3.2 nm^3^ (around 780 e^–^) for [I^–^·(**1**·**1**)·I^–^] per asymmetric unit, which contains one equivalent of dimer (**1**·**1**). These volumes are sufficient for accommodating necessary amounts of (TPP)^+^ (calculated volume of 0.34 nm^3^, 178 e^–^) for both structures (1.40 equiv. for[**1**·(Cl^–^)·**1**] and 0.41 equiv. for [I^–^·(**1**·**1**)·I^–^]).

Because modelling the countercation (TPP)^+^ in the above-mentioned way does not give strong evidence for the composition of crystal structure, we have additionally conducted ^1^H NMR experiments to provide the overall compositions of the crystals to fully corroborate the modelled structures. We have isolated the crystals of [**1**·(Cl^–^)·**1**] and washed them to remove any unbound chlorides. Further ^1^H NMR spectra were obtained after dissolving the crystals in CDCl_3_ to identify the countercation signals. ^1^H NMR signals corresponding to protons in (TPP)^+^ were observed in 1.2 equiv. to [**1**·**1**], corroborating the found amount of chloride in the crystal structure (1.40 equiv.), indicating full complexation with [**1**·**1**] (Supplementary Fig. 2).

Owing to disordered solvent molecules, and also large unit cells, some of the quality factors of these structures (explained in detail in Supplementary Information) resulted in level A or B Alerts in the checkCIF routine implemented in the PLATON software^45^.

### Dimerization studies

Time-dependent dimerization of **1** was probed by UV–vis absorption spectroscopy and ^1^H NMR spectroscopy. The dimerization constant was obtained from the concentrations of monomer and dimer species (from integrating the respective peaks) as observed by ^1^H NMR after 20 h (monomer and dimer species are at slow exchange in ^1^H NMR time scale). The rate constant of dimerization was obtained by fitting time-dependent UV–vis absorption data using second-order kinetics. For both UV–vis and ^1^H NMR studies, the following sample preparation routine was used. A monomeric solution was first prepared in Tol and further mixed with MeCN (*c*(**1**) = 5.5 × 10^−5^ M for UV–vis and 2.2 × 10^−4^ M for ^1^H NMR) to get a 1:1 mixture. For kinetic studies, the measurement was started immediately after the addition of MeCN and measured every 30 min using an automatic time-dependant measurement program in the respective spectrometers.

### Halide-binding studies

Owing to the slow exchange in the NMR time scale, binding constants of halides were obtained by direct evaluation of the concentration of bound and unbound species at equilibrium from ^1^H NMR spectroscopy. For the halide-binding experiments, dimers were prepared in 1:1 Tol:MeCN mixture by equilibrating for 20 h followed by the addition of halides. Furthermore, ^1^H NMR and 2D EXSY were performed to evaluate the binding constants and exchange rates, respectively.

### 2D EXSY

All 2D EXSY experiments with the bromide and chloride complex were carried out after sample equilibration for 20 h (1:1 Tol-*d*_*8*_:MeCN-*d*_*3*_) at 600 MHz with a ^13^C/^1^H cryoprobe. First, we measured the ^1^H spin-lattice relaxation times *T*_1_ individually for each sample and each temperature using the inversion recovery pulse sequence (Bruker pulse program t1ir) with a recycle delay of a minimum of 20 s. EXSY spectra were recorded with the Bruker pulse sequence noesygpphpp (ref. ^46^) at 295 K, 323 K, 333 K and 343 K for mixing times *τ*_m_ between 3 ms and 1,250 ms (refs. ^47,48^). All EXSY data used for quantitative evaluation were acquired with recycling delays of at least 5 × *T*_1_ of the protons in the single benzene holes. The intensities of the diagonal and the exchange signals of the mentioned protons were evaluated by a full relaxation matrix analysis^49^ using the EXSYCALC software (https://mestrelab.com/software/freeware/) to obtain the magnetization rate constants that were converted to chemical exchange rates. More details and additional data are given in Supplementary Information.

### Computational methods

The pore diameter in the bilayer holey nanographene was calculated using pywindow^50^ and the subnanometre channel was constructed using molovol program^51^. The nudged elastic band—climbing image (NEB-CI)^39^ method was used to estimate the energy barrier of halide binding in the bilayer nanographene. NEB-CI was performed using semiempirical GFN2-xTB^41^ level of theory as the size of bilayer nanographene was prohibitive to do higher level DFT calculations. For the chloride complex, a transition state calculation was also performed from the converged structure obtained from the NEB-CI calculation. ESP was calculated from DFT optimized structure using B3LYP/6-31G(d) level of theory.

### Programs used

ALMO-EDA calculations were performed using Q-Chem 5.1 (ref. ^52^) with B3LYP-D3/6-311G(d) level of theory. NEB-CI calculations were performed using ORCA 5.0 (ref. ^53^). Semiempirical GFN2-xTB calculations were performed using xtb program^41^.

## Online content

Any methods, additional references, Nature Portfolio reporting summaries, source data, extended data, supplementary information, acknowledgements, peer review information; details of author contributions and competing interests; and statements of data and code availability are available at 10.1038/s41586-024-08299-8.

## Supplementary information

Supplementary Information This file contains additional data (1H NMR, EXSY and UV–vis) in Supplementary Sections 1–11, including Supplementary Figs. 1–36 and Supplementary Tables 1–10, which support the findings of the Article.

## Data Availability

X-ray crystallographic data are available free of charge from the Cambridge Crystallographic Data Centre under the reference numbers CCDC 2308809, 2308810 and 2369264 (https://www.ccdc.cam.ac.uk/structures/). Additional data supporting the findings are contained in the main text or Supplementary Information. All source files are openly available at Zenodo (10.5281/zenodo.8374020).
