# Supplementary material for: Bilayer nanographene reveals halide permeation through a benzene hole
Source: Nature. 2025 Jan 15;637(8047):854–9. doi: 10.1038/s41586-024-08299-8 (PMC11754092; doi:10.1038/s41586-024-08299-8)
Supplement: Supplementary file 1 — This file contains additional data (1H NMR, EXSY and UV–vis) in Supplementary Sections 1–11, including Supplementary Figs. 1–36 and Supplementary Tables 1–10, which support the findings of the Article. [file 41586_2024_8299_MOESM1_ESM.pdf]

nature portfolio

https://doi.org/10.1038/s41586-024-08299-8

Supplementary information

Bilayer nanographene reveals halide permeation through a benzene hole

In the format provided by the authors and unedited

Supplementary Information

Bilayer nanographene reveals halide permeation through a benzene hole

M. A. Niyas[†], Kazutaka Shoyama[†,‡*], Matthias Grüne[†] and Frank Würthner[†,‡,*]

[†]Institut für Organische Chemie, Universität Würzburg, Am Hubland, 97074 Würzburg, Germany
[‡]Center for Nanosystems Chemistry (CNC), Universität Würzburg, Theodor-Boveri-Weg, 97074 Würzburg, Germany

Table of Contents

1 General Methods 3

2 Synthesis 4

3 Optical and Electronic Properties 9

4 Crystallographic Analysis 10

5 Dimerization and anion binding studies 20

6 2D EXSY NMR experiments 27

7 Theoretical Calculations 31

8 Alternate mechanisms 36

9 NMR Spectroscopy 44

10 Mass Spectrometry 54

11 References 56

1 General Methods

All commercial chemicals and reagents, unless otherwise stated, were used without further purification. All air or moisture sensitive reactions were carried out under a nitrogen atmosphere by standard Schlenk-techniques. Photocyclization was performed at rt using a Rayonet photochemical reactor (RPR–100) with 350 nm lamps (power: 14 W, model: RPR–3500A°, company: Southern New England Ultraviolet Company). Microwave reaction was performed with CEM Discover microwave reactor. Column chromatography was performed on silica-gel with a particle size of 0.040–0.063 mm. Recycling semi-preparative gel-permeation chromatography (GPC) was performed on a Shimadzu Prominence CBM and HPLC grade chloroform as the solvent. Melting points (M.p.) were acquired with an Olympus BX41 polarization microscope and are uncorrected. ^{1}H and ^{13}C NMR spectroscopy was performed with a Bruker Avance III HD 400 MHz or Bruker Avance III HD 600 MHz spectrometer. ^{13}C NMR spectra are broad band proton decoupled. The chemical shifts (*δ*) are reported in parts per million (ppm), reported relative to tetramethylsilane and referenced internally to the residual proton solvent resonances (CD_2Cl_2: 5.32; $CDCl_3$: 7.26, tetrachloroethane-d_2 (TCE-d_2): 6.00, Tol: 7.09) or natural abundance carbon resonances ($CDCl_3$: 77.16). For Tol-d_8:MeCN-d_3 solvent mixture, the most downfield signal of Tol was used as reference (δ = 7.09 ppm). The coupling constants (J) are listed in Hertz (Hz). MALDI-TOF mass spectrometry was performed with a Bruker Daltonics ultrafleXtreme mass spectrometer. ESI-TOF mass spectra were recorded on a Bruker Daltonics micrOTOF-Q III spectrometer. UV/vis absorption measurements were conducted with spectroscopic grade solvents. UV/vis absorption spectra were recorded with a Jasco V-770-ST spectrometer.

The catalyst tris(dibenzylideneacetone)dipalladium(0)-chloroform adduct $[Pd_2(dba)_3]\cdot CHCl_3$,[1] **2**,[2] were synthesized according to the literature. 1-Bromo-3-chlorobenzaldehyde (**5**) was purchased from commercial sources and used without further purification.

2 Synthesis

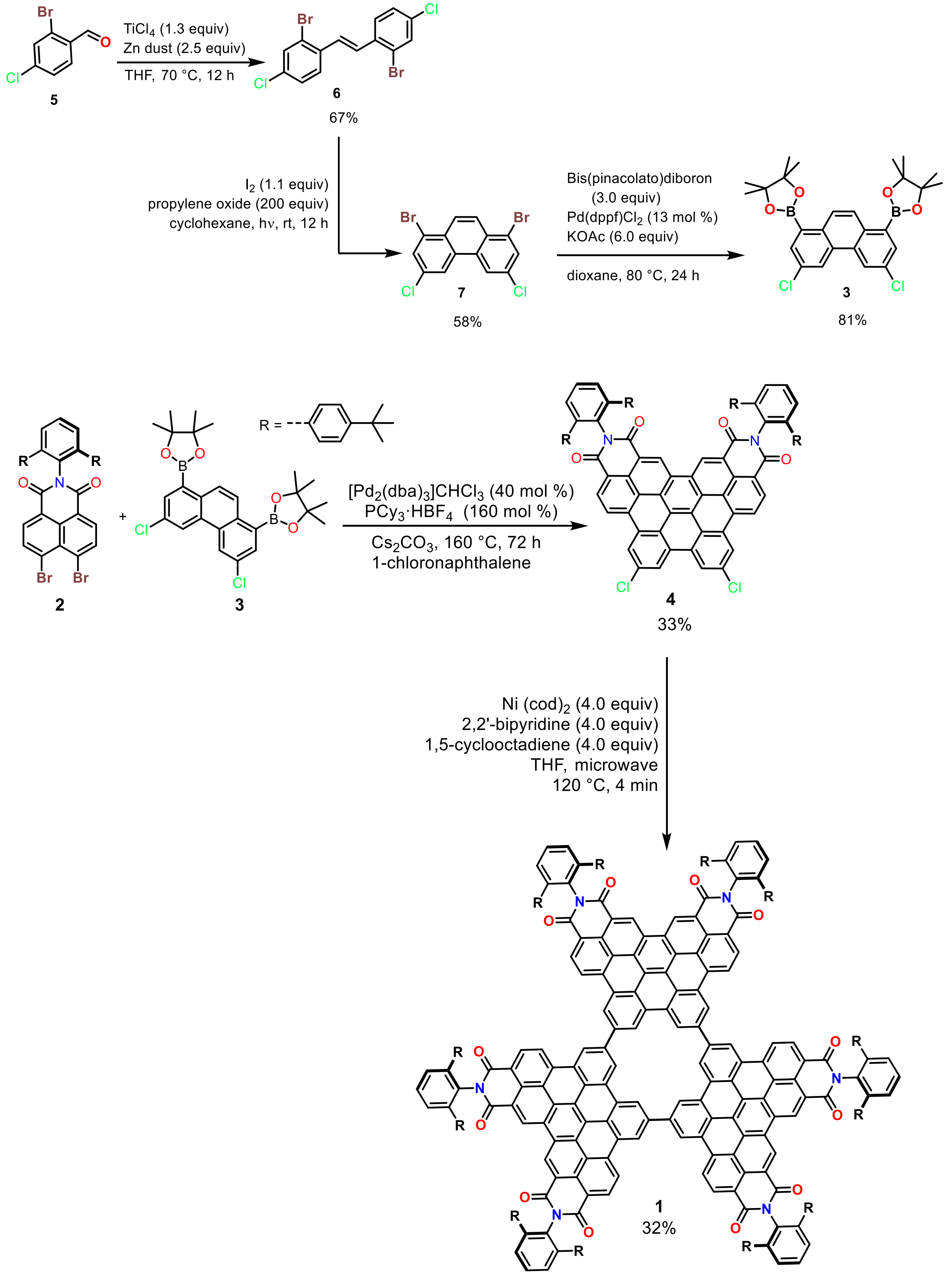

Supplementary Scheme 1. Synthetic scheme for the preparation of **1**.

Synthesis of 2,2'-dibromo-4,4'-dichlorostilbene (6)

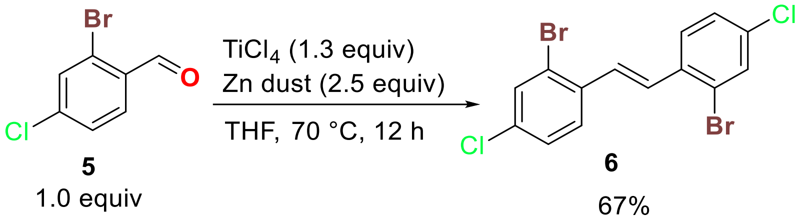

In a 50 mL Schlenk round bottom flask, Zn dust (0.37 g, 5.6 mmol) was mixed in 10 mL dry THF and degassed for 15 min at rt. $TiCl_4$ (0.32 mL, 2.9 mmol) was added through a syringe and was continued stirring for 10 min at rt. Subsequently, 2-bromo-4-chlorobenzaldehyde (**5**, 0.50 g, 2.2 mmol) was dissolved in 2.5 mL dry THF and added to the mixture through a syringe. The mixture was refluxed at 70 °C for 16 h. After cooling to rt, the resulting mixture was filtered. The solvent was removed from the filtrate by rotary evaporator. The residue was dissolved in dichloromethane and washed with brine. The crude product was recrystallized from dichloromethane and methanol to afford the product. Yield: 0.31 g (67%), white crystalline powder. M.p.: 167–168 °C, ^{1}H NMR (400 MHz, $CDCl_3$): δ/ppm = 7.63 (d, J = 8.4 Hz, 2H), 7.61 (s, 2H), 7.32 (d, J = 8.5 Hz, 2H), 7.30 (s, 2H). ^{13}C{^{1}H} NMR (101 MHz, $CDCl_3$): δ/ppm = 135.2, 134.4, 132.8, 129.5, 128.2, 127.8, 124.4. HRMS (APCI$^+$): calcd for $C_{14}H_8Br_2Cl_2$, 405.8349; found, 405.8350.

Synthesis of 1,8-dibromo-3,6-dichlorophenanthrene (7)

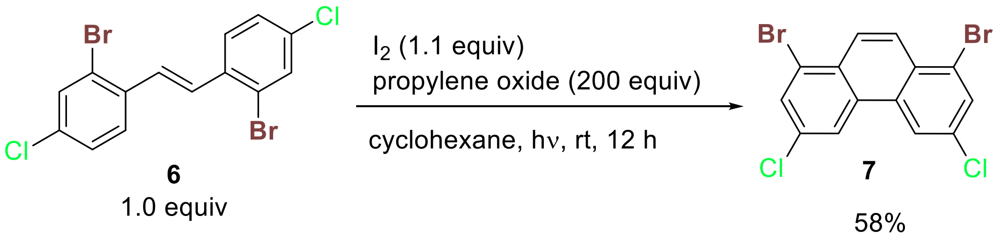

A solution of 2,2'-dibromo-4,4'-dichlorostilbene (**6**, 0.30 g, 0.73 mmol) and I_2 (0.21 g, 0.81 mmol, 1.1 equiv) in 200 mL cyclohexane was stirred in a 500 mL Erlenmeyer flask until all the compounds were fully dissolved. Propylene oxide (8.5 g, 0.14 mol, 200 equiv) was added to the solution and was kept for 16 h in the UV reactor (350 nm, more details in General Methods, Supplementary Information). White crystals of **7** precipitated out and were collected by filtration. Yield: 0.17 g (58%), white crystals. M.p.: 253–254 °C. ^{1}H NMR (400 MHz, $CDCl_3$): δ/ppm = 8.54 (s, 2H), 8.25 (s, 2H), 7.95 (s, 2H). ^{13}C{^{1}H} NMR (101 MHz, $CDCl_3$): δ/ppm = 133.5, 132.3, 131.5, 129.7, 127.0, 124.7, 122.7. HRMS (APCI$^+$): calcd for $C_{14}H_6Br_2Cl_2$, 403.8193; found, 403.8192.

Synthesis of 3,6-dichloro-1,8-phenanthrene diboronic acid pinacol ester (3)

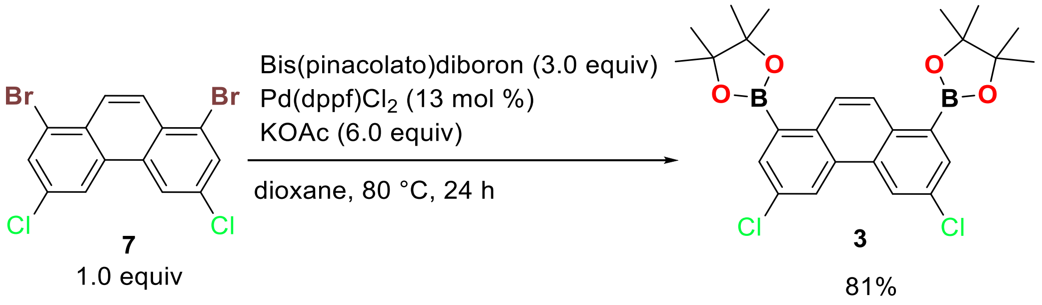

A solution of 1,8-dibromo-3,6-dichlorophenanthrene (**7**, 0.17 g, 0.41 mmol), bis(pinacolato)diboron (0.31 g, 1.2 mmol, 3.0 equiv), [1,1′-bis(diphenylphosphino)ferrocene]dichloropalladium(II) (Pd(dppf)Cl_2, 39 mg, 54 µmol, 13 mol %) and potassium acetate (0.24 g, 2.5 mmol, 6.0 equiv) in degassed dioxane (6 mL) was stirred at 80 °C for 24 h in a Schlenk tube. After the reaction was cooled to rt, dioxane was removed by rotary evaporator. The crude product was then purified by silica-gel column chromatography (eluted with 1:1 dichloromethane:cyclohexane solution). Further recrystallization from dichloromethane solution with methanol layering yielded **3**. Yield: 0.17 g (81%), white powder. M.p.: >300 °C. ^{1}H NMR (400 MHz, $CDCl_3$): δ/ppm = 8.70 (s, 2H), 8.66 (s, 2H), 8.12 (s, 2H), 1.45 (s, 24H). ^{13}C{^{1}H} NMR (101 MHz, $CDCl_3$): δ/ppm = 136.2, 134.4, 132.2 (2C), 130.7, 127.3, 125.4, 84.4, 25.1. HRMS (ESI-TOF^{+}): calcd for $C_{26}H_{30}B_2Cl_2NaO_4$, 521.1599; found, 521.1563.

Synthesis of *N,N*'-bis[2,6-bis(4-*tert*-butylphenyl)phenyl]-5,8-dichloro-peryleno[3,2,1,12b,12a,12,11,10-*pqrstuv*]-picene-1,16:12,13-bis(dicarboximide) (4)

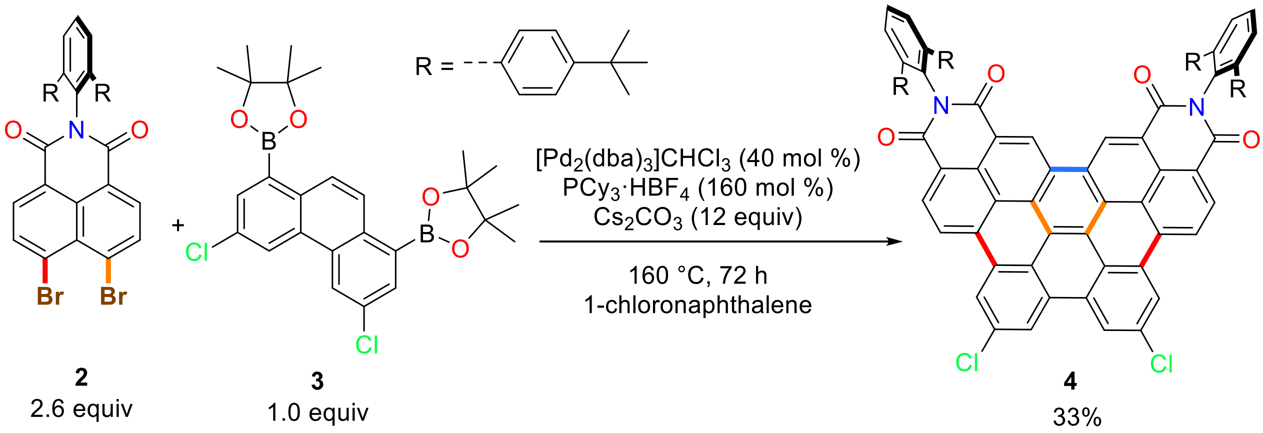

A solution of 4,5-dibromo-1,8-naphthalimide (**2**, 72 mg, 0.10 mmol, 2.6 equiv), 3,6-dichloro-1,8-phenanthrene diboronic acid bis(pinacol)ester (**3**, 20 mg, 0.040 mmol, 1.0 equiv), tris(dibenzylideneacetone)dipalladium(0)-chloroform adduct ([$Pd_2(dba)_3$]·$CHCl_3$, 17 mg, 16 µmol, 40 mol%), tricyclohexylphosphine tetrafluoroborate (PCy_3·HBF_4, 24 mg, 64 µmol, 160 mol %), cesium carbonate (Cs_2CO_3, 0.16 g, 0.48 mmol, 12 equiv) and 1-chloronaphthalene (0.70 mL) was taken in a Schlenk

tube and heated at 160 °C for 72 h. After cooling to rt, the reaction mixture was filtered with cyclohexane over a silica pad to remove 1-chloronaphthalene. The crude product was purified by silica-gel column chromatography (eluted with dichloromethane). Yield: 17 mg (33%), red solid. ^{1}H NMR (400 MHz, TCE-d_2): δ/ppm = 10.06 (s, 2H), 8.47 (d, J = 8.0 Hz, 2H), 8.08 (s, 2H), 7.93 (d, J = 8.1 Hz, 4H), 7.75–7.79 (m, 8H), 7.66 (d, J = 7.4 Hz, 4H), 7.33 (d, J = 8.5 Hz, 4H), 7.05 (s, 2H), 6.98 (d, J = 8.5 Hz, 4H), 0.94 (s, 18H), 0.80 (s, 18H). ^{13}C{^{1}H} NMR (101 MHz, $CDCl_3$): δ/ppm = 163.7, 163.3, 150.2, 149.8, 142.0, 141.1, 137.6, 135.8, 134.2, 131.4, 131.1, 130.9, 130.3, 129.3, 129.2, 128.9, 128.8, 127.9, 126.0, 125.5, 124.6, 124.5, 124.5, 124.0, 122.6, 122.0, 121.7, 121.5, 121.3, 120.7, 120.5, 34.2, 33.8, 30.8, 30.7. 1 MS (MALDI-TOF^{-}): calcd for $C_{90}H_{68}Cl_2N_2O_4$, 1310.4556; found, 1310.5614 [M^{-}]

Synthesis of *N,N',N'',N''',N'''',N'''''*-hexakis[2,6-bis(4-*tert*-butylphenyl)phenyl]-1,2,3(5,8)peryleno-[3,2,1,12b,12a,12,11,10-*pqrstuv*]picenatriphane-1^{1},1^{16}:1^{12},1^{13}:2^{1},2^{16}:2^{12},2^{13}:3^{1},3^{16}:3^{12},3^{13}-hexakis(di-carboximide) (1)

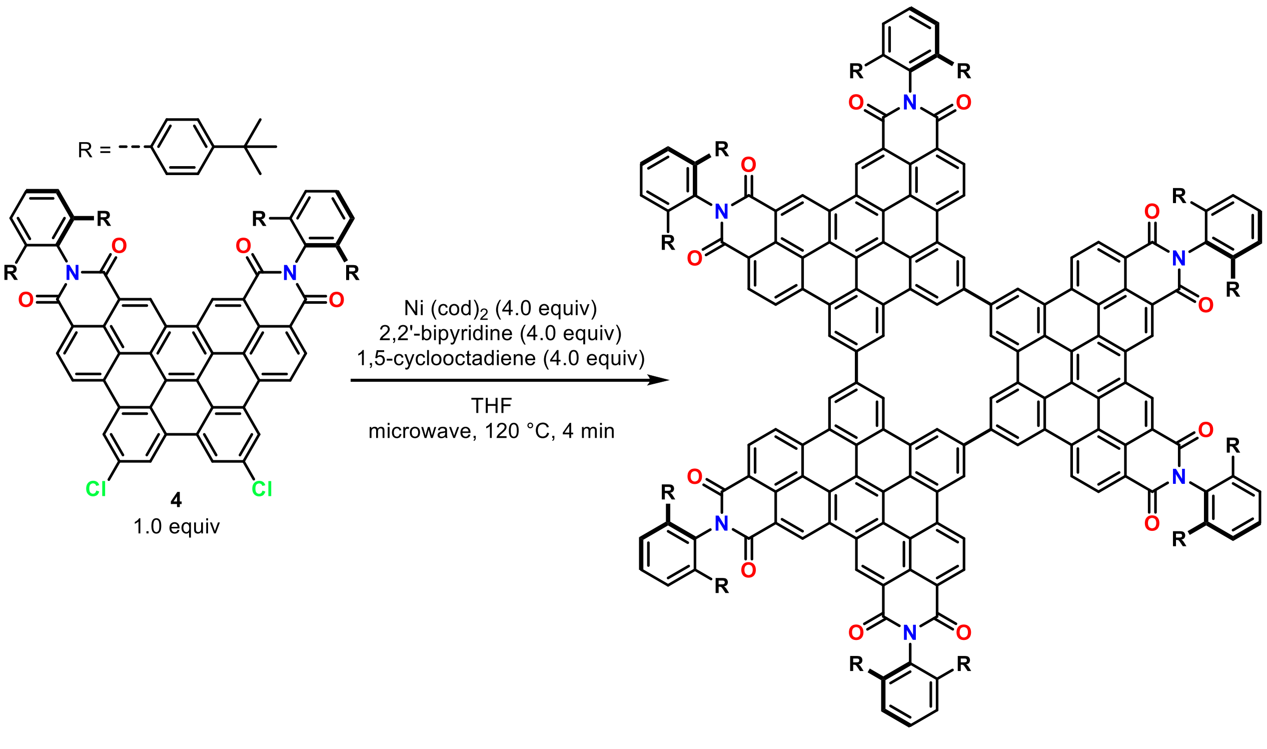

Dichlorinated bisimide (**4**, 20 mg, 0.15 mmol, 1.0 equiv), 2,2'-bipyridine (9.5 mg, 61 µmol, 4.0 equiv), 1,5-cyclooctadiene (7.4 µL, 61 µmol, 4.0 equiv) and Ni(cod)$_2$ (17 mg, 61 µmol, 4.0 equiv) were dissolved in 3 mL dry and degassed THF solution in a 5 mL microwave reactor pressure tube. The reaction was stirred at 120 °C for 4 min in a microwave reactor. After the reaction was cooled to rt, THF was removed by rotary evaporator. The crude product was purified by silica-gel column chromatography (elution solvent: 1:0 to 0:1 CH_2Cl_2:ethylacetate) and semi-preparative gel-permeation chromatography (GPC, in $CHCl_3$). Product fractions obtained by GPC separation were dissolved in THF containing excess of TBACl and layered with

cyclohexane (THF:cyclohexane = 1:4) overnight to afford precipitation of pure chloride complexes ([**1**·(Cl^-)·**1**] complexes are not soluble in low polar solvent mixtures). Precipitates were filtered and dissolved in CH_2Cl_2 and washed with water in an extraction funnel to remove TBACl. A final recrystallization from CH_2Cl_2:methanol (1:4) mixture yielded pure **1** as a red solid. Yield: 6 mg (32%). ^1H NMR (400 MHz, CD_2Cl_2): δ/ppm = 10.95 (s, 6H), 10.62 (s, 6H), 10.51 (s, 6H), 9.87 (d, J = 8.6 Hz, 6H), 9.25 (d, J = 8.1 Hz, 6H), 7.75 (t, J = 7.9 Hz, 6H), 7.63 (d, J = 7.8 Hz, 12H), 7.45 (d, J = 8.7 Hz, 24H), 7.21 (d, J = 8.7 Hz, 24H), 1.00 (s, 72H).). $^{13}C\{^1H\}$ NMR (101 MHz, CD_2Cl_2): δ/ppm = 164.9, 150.6, 142.3, 138.1, 136.7, 135.4, 132.6, 132.2, 131.9, 130.7, 130.2, 130.0, 129.6, 128.4, 128.0, 126.7, 126.5, 125.4, 125.1, 124.6, 124.2, 123.0, 122.9, 122.7, 122.3, 121.6, 31.1. HRMS (MALDI-TOF, negative mode, DCTB in chloroform): calcd. for $C_{270}H_{204}N_6O_{12}$, 3724.5684; found, 3724.5964 [M^-]

3 Optical and Electronic Properties

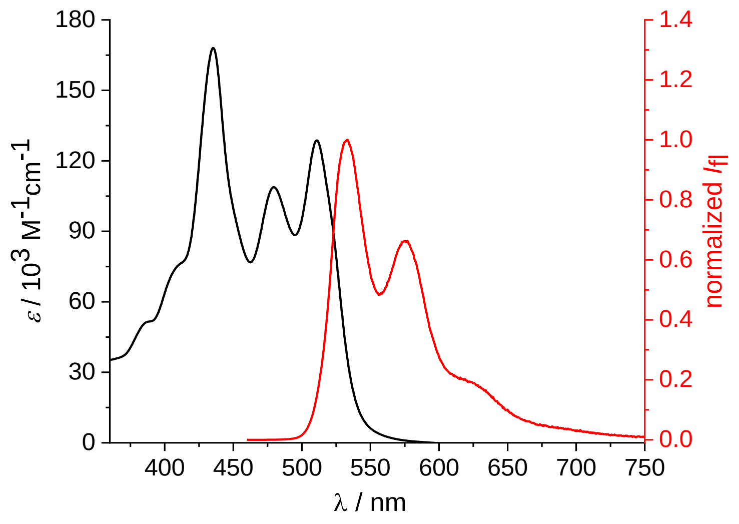

Supplementary Figure 1. UV/vis absorption (black, c = 2.6×10^{-6} M) and normalized fluorescence (red, λ_{ex} = 435 nm, c ~ 10^{-7} M) spectra of **1** in chloroform solutions at room temperature.

Supplementary Table 1. Summary of the optical properties of **1** in chloroform solution at room temperature.[a]

	λ_{abs} /nm (ε / M^{-1} cm^{-1})	λ_{em} / nm[b]	Φ_{fl} / %[c]	τ / ns[d]
**1**[a]	511 (129000)<br>435 (168000)	533	32 ± 1	5.78 ± 0.01

[a]Measurements were conducted in chloroform solutions at room temperature (UV/vis: c~10^{-6} M; fluorescence: c~10^{-7} M). [b]Excitation wavelength: λ_{ex} = 530 nm. [c]Determined using the relative method (A < 0.05) with perylene red as reference (Φ_{fl} ($CHCl_3$) = 96%). [d]Excitation wavelength: λ_{ex} = 435 nm; detection wavelength: λ_{em} = 533 nm

4 Crystallographic Analysis

Single crystals of [**1**·**1**] suitable for X-ray diffraction could be grown by slow diffusion of *n*-hexane into a chloroform/DMSO solution of **1** (c(**1**) ≈ 2 ×10^{-4} M, 293 K) over a week. Single crystals of [**1**·(Cl^-)·**1**] was grown by mixing **1** and tetraphenylphosphonium chloride (TPPCl) in 1:1 Tol:MeCN (c(**1**) ≈ 2 ×10^{-4} M, c(TPPCl) ≈ 3 ×10^{-4} M, 293 K) and slow diffusion of MeCN over a week. Single crystals of [I^-·(**1**·**1**)·I^-] was grown by mixing **1** and tetraphenylphosphonium iodide (TPPI) in 1:1 Tol:MeCN (c(**1**) ≈ 3 ×10^{-4} M, c(TPPI) ≈ 3 ×10^{-3} M, 293 K) and slow diffusion of MeCN over a week.

Single crystal X-ray crystallography were performed on a Bruker D8 Quest Diffractometer with a PhotonII detector using Cu K_α radiation for [**1**·**1**] or the P11 beamline at DESY for [**1**·(Cl^-)·**1**] and [I^-·(**1**·**1**)·I^-]. The structures were solved using SHELXT,[3] expanded with Fourier techniques and refined using the SHELXL software package.[4] Hydrogen atoms were assigned at idealized positions and were included in the calculation of structure factors. All non-hydrogen atoms in the major disorder part of main residues were refined anisotropically. In all crystal structures some of the *N*-substituents (bis(*tert*-butyl)-*meta*-terphenyl groups) and solvent molecules were disordered and modelled with constraints and restraints using standard SHELX commands EADP, DFIX, FLAT, SAME, SADI, DELU, SIMU, CHIV, ISOR, and RIGU.

The amount of chloride found in [**1**·(Cl^-)·**1**] summed up to 1.40 equivalents, comprising fully occupied (1.00 equivalent) chloride ion in the cavity of dimer (**1**·**1**) and a partially occupied chloride ion at the periphery (Supplementary Figure 3, 0.40 equivalents). In the crystal structure of [I^-·(**1**·**1**)·I^-], the iodide ions placed on top of the both holes of dimer (**1**·**1**) are only partially occupied with occupancies of 0.191 and 0.142, respectively. The iodide at the periphery (Supplementary Figure 3) had an occupancy of 0.072. These numbers account for the equivalents of respective iodide for each dimer (**1**·**1**).

For the refinement of [**1**·(Cl^-)·**1**] and [I^-·(**1**·**1**)·I^-], the disordered counter cation $(TPP)^+$ residues were modelled either by rigid group constraints of the whole molecule using the FRAG command of SHELXL or by a combination of the phenyl ring constrain (AFIX 66 command) and distance restraints (DFIX and DANG commands). For [**1**·(Cl^-)·**1**], the geometry of $(TPP)^+$ optimized by density functional theory (DFT) calculations at the B3LYP/6-311+G(d,p) level of theory was used. For [I^-·(**1**·**1**)·I^-], the four phenyl rings of $(TPP)^+$ are constrained by AFIX 66 command of SHELXL and distances between P and phenyl rings were restrained by DFIX and DANG commands. The common plane restraints using the FLAT command of SHELXL was also applied to stabilize the connection between P and phenyl rings as well. The rigid group constraints were also applied for heavily disordered acetonitrile molecules using the geometry obtained by the DFT PBE1PBE/6-311++G(3df,3pd) level of theory.[5] The analysis of solvent accessible voids identified a volume of ca. 1.4 nm^3 (ca. 340 e^-) for [**1**·(Cl^-)·**1**] and ca. 3.2 nm^3 (ca. 780 e^-) for [I^-·(**1**·**1**)·I^-] per asymmetric unit, which contains one equivalent of dimer (**1**·**1**). These volumes are sufficient for accommodating

necessary amounts of $[P(Ph)_4]^+$ (calculated volume of 0.34 nm^3, 178 e^-) for both structures (1.40 equivalents for[**1**·(Cl^-)·**1**] and 0.41 equivalents for [I^-·(**1**·**1**)·I^-]).

Because modelling the counter cation $(TPP)^+$ in abovementioned way does not give strong evidence for the composition of crystal structure, we have additionally conducted ^{1}H NMR experiments to provide the overall compositions of the crystals to fully corroborate the modelled structures. We have isolated the crystals of [**1**·Cl^-·**1**] and washed it with methanol to remove any unbound chlorides. Further ^{1}H NMR spectra was obtained after dissolving the crystals in $CDCl_3$ to identify the counter-cation signals. ^{1}H NMR signals corresponding to protons in tetraphenyl phosphonium cation was observed in 1.2 equivalents to [**1**·**1**], corroborating the found amount of chloride in the crystal structure (1.40 equivalents), indicating full complexation with [**1**·**1**] (Supplementary Figure 2).

Owing to disordered solvent molecules, and additionally due to large unit cells, some of the quality factors of these structures (explained below in details) resulted in level A and/or B Alerts in the checkcif routine implemented in the PLATON software.[6]

Explanation for the level A and B alerts for the crystal structure data of [1·1]

PLAT260_ALERT_2_B Large Average Ueq of Residue Including S1_112 0.575 Check
PLAT260_ALERT_2_B Large Average Ueq of Residue Including S1_114 0.516 Check
PLAT260_ALERT_2_B Large Average Ueq of Residue Including S1_116 0.517 Check
PLAT260_ALERT_2_B Large Average Ueq of Residue Including S1_118 0.504 Check
PLAT260_ALERT_2_B Large Average Ueq of Residue Including S1_119 0.378 Check
PLAT260_ALERT_2_B Large Average Ueq of Residue Including S1_120 0.371 Check
PLAT340_ALERT_3_B Low Bond Precision on C-C Bonds .... 0.01603 Ang.
Large displacement parameters of the DMSO residues and low bond precision (C-C) for this crystal structure arises from large unit cell volume and solvent accessible voids, which results in weak diffraction in the outer resolution shells.

Explanation for the level A alerts for the crystal structure data of [1·Cl⁻·1]

PLAT026_ALERT_3_A Ratio Observed / Unique Reflections (too) Low .. 24% Check
PLAT029_ALERT_3_A _diffrn_measured_fraction_theta_full value Low . 0.934 Why?
PLAT084_ALERT_3_A High wR2 Value (i.e. > 0.25) .... 0.54 Report
The apparent resolution for the diffraction data of this crystal structure is until 1.0 angstrom (I/sigma > 2.0). This caused many reflections rejected during the integration and scaling steps, which resulted in low completeness for resolution shells below that limit and low observed / unique reflections ratio. The high final wR2 value also arises from weakly diffracting lower resolution shells.

PLAT241_ALERT_2_B High ’MainMol’ Ueq as Compared to Neighbors of C56_1 Check
PLAT241_ALERT_2_B High ’MainMol’ Ueq as Compared to Neighbors of C75_2 Check
PLAT241_ALERT_2_B High ’MainMol’ Ueq as Compared to Neighbors of C47_3 Check
PLAT242_ALERT_2_B Low ’MainMol’ Ueq as Compared to Neighbors of C51_1 Check
PLAT242_ALERT_2_B Low ’MainMol’ Ueq as Compared to Neighbors of C61_1 Check
PLAT242_ALERT_2_B Low ’MainMol’ Ueq as Compared to Neighbors of C77_1 Check
PLAT242_ALERT_2_B Low ’MainMol’ Ueq as Compared to Neighbors of C51_2 Check
PLAT242_ALERT_2_B Low ’MainMol’ Ueq as Compared to Neighbors of C71_2 Check
PLAT242_ALERT_2_B Low ’MainMol’ Ueq as Compared to Neighbors of C74_2 Check
PLAT242_ALERT_2_B Low ’MainMol’ Ueq as Compared to Neighbors of C77_11 Check
PLAT242_ALERT_2_B Low ’MainMol’ Ueq as Compared to Neighbors of C61_13 Check
PLAT250_ALERT_2_B Large U3/U1 Ratio for <U(i,j)> Tensor(Resd 1) 4.7 Note
PLAT250_ALERT_2_B Large U3/U1 Ratio for <U(i,j)> Tensor(Resd 2) 5.3 Note
PLAT260_ALERT_2_B Large Average Ueq of Residue Including Cl1_21 0.421 Check
The *meta*-terphenyl groups and *tert*-butyl groups thereof in this crystal structure contain disorder and this caused prolate ellipsoids or ellipsoids with larger/smaller U_{eq} compared to neighbouring atoms. Ellipsoids in the solvent residues are also large presumably due to large thermal motions in the crystal structure.

PLAT315_ALERT_2_B Singly Bonded Carbon Detected (H-atoms Missing). C2_105 Check
Some of the acetonitrile solvates in this crystal structure could only be modelled by using rigid group constrains without adding hydrogen atoms. The refinement of main rotation axis was instable due to heavy disorder, which hampered assigning the positions of hydrogen atoms.

PLAT934_ALERT_3_B Number of (Iobs-Icalc)/Sigma(W) > 10 Outliers .. 6 Check
-4 2 2, 12 2 3, 1 3 3, -16 0 6, 8 3 8, 0 3 11,
The heavy disorder throughout the whole molecule as well as the solvent molecules in the solvent accessible voids caused many outliners. Because crystallography is a statistic analysis, these outliners were not removed by the OMIT instruction to avoid modification of measurement data.

Explanation for the level A alerts for the crystal structure data of [I^{-}·(1·1)·I^{-}],

PLAT026_ALERT_3_A Ratio Observed / Unique Reflections (too) Low .. 29% Check
PLAT029_ALERT_3_A _diffrn_measured_fraction_theta_full value Low . 0.937 Why?
PLAT084_ALERT_3_A High wR2 Value (i.e. > 0.25) .... 0.52 Report
The apparent resolution for the diffraction data of this crystal structure is until 1.0 angstrom (I/sigma > 2.0). This caused many reflections rejected during the integration and scaling steps, which resulted in low completeness for resolution shells below that limit and low observed / unique reflections ratio. The high final wR2 value also arises from weakly diffracting lower resolution shells.

PLAT245_ALERT_2_A U(iso) H7_101 Smaller than U(eq) C6_313 by 0.157 Ang**2 Residues 101 (toluene) and 313 (tetraphenylphosphine cation) are in different parts. Because they occupy the same space and should not exist in the same asymmetric unit, the displacement factors do not need to coincide each other.

PLAT220_ALERT_2_B NonSolvent Resd 1 C Ueq(max)/Ueq(min) Range 7.8 Ratio
PLAT220_ALERT_2_B NonSolvent Resd 2 C Ueq(max)/Ueq(min) Range 6.9 Ratio
PLAT241_ALERT_2_B High 'MainMol' Ueq as Compared to Neighbors of C49_11 Check
PLAT242_ALERT_2_B Low 'MainMol' Ueq as Compared to Neighbors of C45_11 Check
PLAT242_ALERT_2_B Low 'MainMol' Ueq as Compared to Neighbors of C48_11 Check
PLAT242_ALERT_2_B Low 'MainMol' Ueq as Compared to Neighbors of C51_11 Check
PLAT242_ALERT_2_B Low 'MainMol' Ueq as Compared to Neighbors of C51_13 Check
PLAT242_ALERT_2_B Low 'MainMol' Ueq as Compared to Neighbors of C51_1 Check
PLAT242_ALERT_2_B Low 'MainMol' Ueq as Compared to Neighbors of C61_1 Check
PLAT242_ALERT_2_B Low 'MainMol' Ueq as Compared to Neighbors of C77_3 Check
The *meta*-terphenyl groups and *tert*-butyl groups thereof in this crystal structure contain disorder and this caused prolate ellipsoids, ellipsoids with larger/smaller U_{eq} compared to neighbouring atoms, and/or large Ueq(max)/Ueq(min) Range.

PLAT250_ALERT_2_B Large U3/U1 Ratio for <U(i,j)> Tensor(Resd 10) 4.3 Note
Large U3/U1 ratio observed for several solvent residues are due to heavy disorder in the solvent accessible voids.

PLAT260_ALERT_2_B Large Average Ueq of Residue Including I2_21 0.303 Check
PLAT260_ALERT_2_B Large Average Ueq of Residue Including P1_301 0.323 Check
PLAT260_ALERT_2_B Large Average Ueq of Residue Including P1_303 0.318 Check
PLAT260_ALERT_2_B Large Average Ueq of Residue Including C1_104 0.396 Check
PLAT260_ALERT_2_B Large Average Ueq of Residue Including C1_112 0.306 Check
PLAT260_ALERT_2_B Large Average Ueq of Residue Including N1_209 0.318 Check
PLAT260_ALERT_2_B Large Average Ueq of Residue Including N1_215 0.305 Check
PLAT260_ALERT_2_B Large Average Ueq of Residue Including N1_216 0.352 Check
PLAT260_ALERT_2_B Large Average Ueq of Residue Including N1_217 0.307 Check
PLAT260_ALERT_2_B Large Average Ueq of Residue Including N1_218 0.365 Check
PLAT260_ALERT_2_B Large Average Ueq of Residue Including N1_223 0.487 Check
PLAT260_ALERT_2_B Large Average Ueq of Residue Including N1_224 0.441 Check
PLAT260_ALERT_2_B Large Average Ueq of Residue Including N1_225 0.504 Check
PLAT260_ALERT_2_B Large Average Ueq of Residue Including N1_227 0.309 Check
PLAT260_ALERT_2_B Large Average Ueq of Residue Including N1_228 0.427 Check

PLAT260_ALERT_2_B Large Average Ueq of Residue Including N1_229 0.306 Check
PLAT260_ALERT_2_B Large Average Ueq of Residue Including N1_231 0.709 Check
PLAT260_ALERT_2_B Large Average Ueq of Residue Including N1_233 0.687 Check
PLAT260_ALERT_2_B Large Average Ueq of Residue Including N1_212 0.387 Check
PLAT260_ALERT_2_B Large Average Ueq of Residue Including N1_220 0.351 Check
PLAT260_ALERT_2_B Large Average Ueq of Residue Including N1_226 0.499 Check
PLAT260_ALERT_2_B Large Average Ueq of Residue Including N1_236 0.480 Check
Large average Ueq observed for iodide and several solvent residues is due to heavy disorder in the solvent accessible voids.

PLAT315_ALERT_2_B Singly Bonded Carbon Detected (H-atoms Missing). C1_210 Check
Some of the acetonitrile solvates in this crystal structure could only be modelled by using rigid group constrains without adding hydrogen atoms. The refinement of main rotation axis was instable due to heavy disorder, which hampered assigning the positions of hydrogen atoms.

The diffraction resolution of the crystallographic data for [**1**·(Cl^-)·**1**] lay, according to the typical definition of $I/\sigma > 2$, at 1.0 Å. Nevertheless, we have used all the diffraction data measured down to a minimum of 0.66 Å with rather low completeness between 0.80 and 0.66 Å due to following reasons. First, it has been proposed that inclusion of diffraction data lower than the resolution limit may make some improvements and does not deteriorate the model.[7] Second, we find that the refinement of such large crystal structure that includes heavy disorder both in the main residues and the solvent accessible voids are more stable with inclusion of high-resolution diffraction data below typical resolution limit.

Regarding the crystal structure of [**1**·(Cl^-)·**1**], we also considered the possibility that the peak assigned as the second chloride ion at the bay region (Supplementary Figure 3) might be attributed to water. We tried refinement by placing H_2O instead of the second Cl^-, which resulted in a higher wR_2 value (0.5415) than that obtained for Cl^- (0.5404). Additionally, it is unlikely that such water molecule would be in the cavity surrounded by *tert*-butyl phenyl groups and the π-conjugated core scaffold of **1** without hydrogen bond acceptor sites. We thus consider Cl^- as a more appropriate assignment for the found electron density at the bay region.

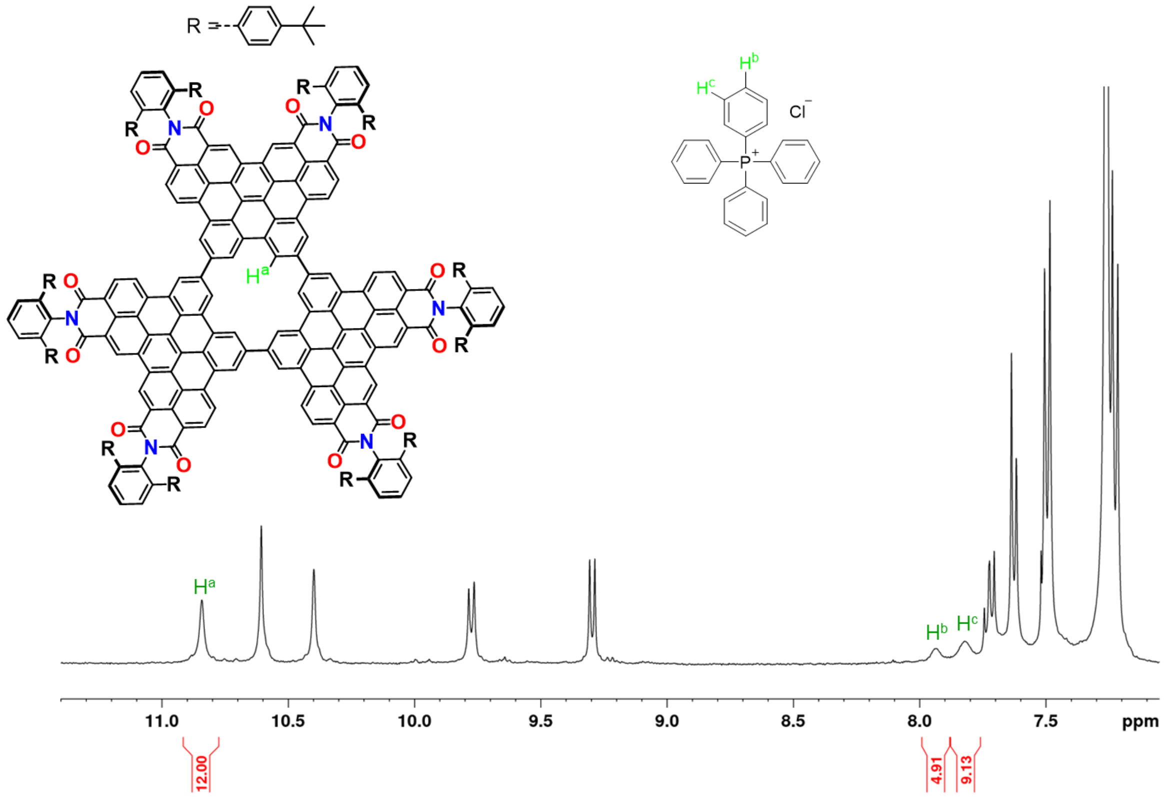

Supplementary Figure 2. (a) ^{1}H NMR spectra of crystals of [**1**·(Cl⁻)·**1**] dissolved in $CDCl_3$. Crystals were isolated, washed with methanol, dried under vacuum, and dissolved in $CDCl_3$ before performing ^{1}H NMR experiment.

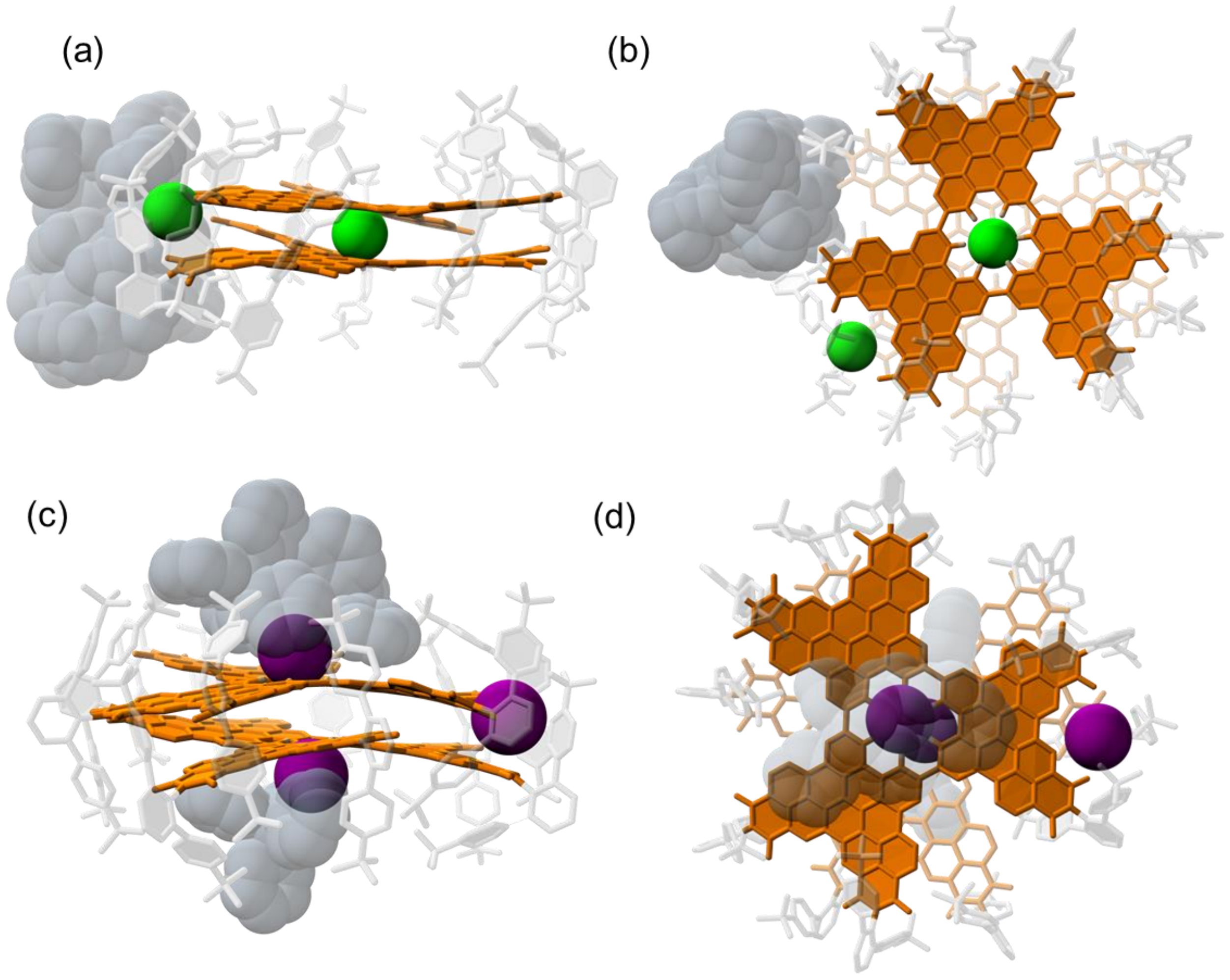

Supplementary Figure 3. (a, b) X-ray crystal structure of [**1**·(Cl⁻)·**1**] showing occupancy of chlorides at the hole and at the periphery of dimer in (a) side view and (b) top view. (c, d) X-ray crystal structure of [I⁻·(**1**·**1**)·I⁻] complex showing all three iodide occupying sites in (c) side view and (d) top view. Crystal structure shows the blocking of hole by iodide. Disordered occupancy of TPP^+ counter cations are shown in grey spheres

Supplementary Table 2. Crystallographic data and structure refinement for dimer of **1** ([**1**·**1**]).

Identification code	CCDC 2308809
Empirical formula	$C_{601.83}H_{587.50}Cl_9 N_{12}O_{53.42}S_{29.42}$
Formula weight	10105.12
Temperature	100(2) K
Wavelength	1.54178 Å
Crystal system	Trigonal
Space group	*R3*
Unit cell dimensions	*a* = 31.3052(16) Å
	b = 31.3052(16) Å
	c = 48.324(3) Å
	α = 90°
	β = 90°
	γ = 120°
Volume	41014(5) $Å^3$
Z	3
Density (calculated)	1.227 g/cm^3
Absorption coefficient	2.012 mm^{-1}
F(000)	16000.4
Crystal size	0.209 × 0.171 × 0.144 mm^3
Theta range for data collection	2.449 to 83.339°.
Index ranges	$-39 \le h \le 39$, $-40 \le k \le 39$, $-61 \le l \le 58$
Reflections collected	298654
Independent reflections	39305 [R_{int} = 0.0988]
Completeness to theta = 67.679°	100.0%
Absorption correction	Semi-empirical from equivalents
Max. and min. transmission	0.7543 and 0.5768
Refinement method	Full-matrix least-squares on F^2
Data / restraints / parameters	39305 / 3387 / 2548
Goodness-of-fit on F^2	1.056
Final *R* indices [$I > 2\sigma(I)$]	R_1 = 0.0975, wR_2 = 0.2473
R indices (all data)	R_1 = 0.1399, wR_2 = 0.2943
Largest diff. peak and hole	0.595 and –0.462 e·$Å^{-3}$

Supplementary Table 3. Crystallographic data and structure refinement for tricomponent 2:1 cocrystal from **1**:TPPCl ([**1**·(Cl⁻)·**1**]).

Identification code	CCDC 2308810
Empirical formula	$C_{618.26}H_{481.50}Cl_{1.40}N_{16.20}O_{24}P_{1.40}$
Formula weight	8614.66
Temperature	100(2) K
Wavelength	0.61992 Å
Crystal system	Monoclinic
Space group	*P2₁/n*
Unit cell dimensions	*a* = 30.906(3) Å
	b = 27.412(3) Å
	c = 54.609(5) Å
	α = 90°.
	β = 90.1300(10)°.
	γ = 90°.
Volume	46264(7) $Å^3$
Z	8
Density (calculated)	1.237 g/cm^3
Absorption coefficient	0.065 mm^{-1}
F(000)	18165.1
Crystal size	0.100 × 0.050 × 0.050 mm^3
Theta range for data collection	0.650 to 28.109°.
Index ranges	$-46 \le h \le 46$, $-35 \le k \le 35$, $-75 \le l \le 77$
Reflections collected	879287
Independent reflections	115444 [R_{int} = 0.1110]
Completeness to theta = 21.836°	93.4%
Absorption correction	None
Refinement method	Full-matrix least-squares on F^2
Data / restraints / parameters	115444 / 18122 / 5875
Goodness-of-fit on F^2	1.062
Final R indices [$I > 2\sigma(I)$]	R_1 = 0.1547, wR_2 = 0.4202
R indices (all data)	R_1 = 0.3111, wR_2 = 0.5404
Largest diff. peak and hole	0.553 and –0.954 e·$Å^{-3}$

Supplementary Table 4. Crystallographic data and structure refinement for tetracomponent 2:2 cocrystal from **1**:TPPI ([I^-·(**1**·**1**)·I^-]

Identification code	CCDC 2369264
Empirical formula	$C_{641.16}H_{498.21}I_{0.41}N_{36.48}O_{24}P_{0.41}$
Formula weight	9161.63
Temperature	100(2) K
Wavelength	0.61991 Å
Crystal system	Monoclinic
Space group	*P2₁/n*
Unit cell dimensions	a = 19.130(9) Å
	b = 53.96(2) Å
	c = 54.45(2) Å
	α = 90°
	β = 97.669(11)°
	γ = 90°
Volume	55702(41) Å^3
Z	4
Density (calculated)	1.092 g/cm^3
Absorption coefficient	0.066 mm^{-1}
F(000)	19280.4
Crystal size	0.050 × 0.030 × 0.030 mm^3
Theta range for data collection	0.465 to 27.909°.
Index ranges	$-24 \leq h \leq 24$, $-59 \leq k \leq 63$, $-69 \leq l \leq 69$
Reflections collected	1057074
Independent reflections	138822 [R_{int} = 0.1409]
Completeness to theta = 67.679°	93.7%
Absorption correction	None
Max. and min. transmission	0.7543 and 0.5768
Refinement method	Full-matrix least-squares on F^2
Data / restraints / parameters	138822 / 22695 / 7200
Goodness-of-fit on F^2	0.972
Final *R* indices [$I > 2\sigma(I)$]	R_1 = 0.1483, wR_2 = 0.3951
R indices (all data)	R_1 = 0.2914, wR_2 = 0.5226
Largest diff. peak and hole	0.740 and –0.501 e·Å^{-3}

5 Dimerization and anion binding studies

General Considerations

Dimerization

Time-dependant dimerization of **1** was probed by UV/Vis absorption spectroscopy and ^{1}H NMR spectroscopy. The dimerization constant was obtained from the concentrations of monomer and dimer species as observed by ^{1}H NMR after 20 h (species in slow exchange). The rate constant of dimerization was obtained by fitting time-dependant UV/Vis absorption data using second order kinetics. For both UV/Vis and ^{1}H NMR studies, following sample preparation routine was used. A monomeric solution was first prepared in Tol and further mixed with MeCN (c(**1**) = 5.5×10^{-5} M for UV/Vis and 2.2×10^{-4} for ^{1}H NMR) to get a 1:1 mixture. The measurement was started immediately after addition of MeCN for kinetic studies. Spectra at every 30 min were measured using an automatic time-dependant measurement program in respective spectrometers.

For equilibrium dimerization constant, due to the slow exchange, monomer and dimer concentrations could be identified separately in ^{1}H NMR (after 20 h equilibration time) and thus integrating the respective peaks gave the concentration of monomer and dimer at equilibrium. From the equilibrium concentration of monomer and dimer, dimerization constant was calculated using eq. (2).

$$2\ \mathrm{M} \rightleftharpoons \mathrm{D} \qquad (1)$$

$$K_{\mathrm{d}} = \frac{c_{\mathrm{D}}}{{c_{\mathrm{M}}}^{2}}. \qquad (2)$$

Here, K_d is the dimerization constant. c_D and c_M are concentration of dimer and monomer respectively at equilibrium. With a known total concentration of sample and slow exchange between equilibrium species, c_D and c_M are obtained from the integration of ^{1}H NMR spectra after 20 h.

Activation energy of dimerization (E_d) and activation energy of bromide binding (E_{Br}) was calculated from Arrhenius equation by linear fitting of ln(k) to 1/T according to equation (3). A is the preexponential factor in Arrhenius equation. E_d/E_{Br} is then calculated from the slope (slope = $-E_{d/Br}/R$) where R is the gas constant (8.314 JK^{-1}mol^{-1}).

$$\ln(k) = \frac{-E_d}{R} \times \frac{1}{T} + \ln(A) \quad (3)$$

Halide binding

Halide binding by dimers was evaluated by directly calculating concentrations of equilibrium species from ^{1}H NMR spectra at 295 K. This was possible due to the slow exchange between equilibrium species involved.

$$\mathrm{D} + \mathrm{X}^- \rightleftharpoons \mathrm{D} \cdot \mathrm{X}^- \quad (4)$$

$$K_{\mathrm{X}^-} = \frac{c_{\mathrm{D \cdot X}^-}}{c_{\mathrm{D}} \times c_{\mathrm{X}^-}} \quad (5)$$

D is dimer and X^- is any halide. K_{X^-} is the binding constant corresponding to halide binding in dimers. $c_{D \cdot X^-}$, c_D and c_{X^-} are concentration of dimer·X^-, free dimer, and free halide respectively at equilibrium. Bromide binding constant was calculated directly. For chloride and fluoride, as the binding constant is higher in the micromolar range, almost all the dimers are converted to D·Cl^-/ D·F^- with only half equivalence of chloride/fluoride (c(Cl^-/F^-) = 0.5×c(**1**)). Thus, we performed a competitive ^{1}H NMR experiment to calculate the binding constant of chloride and fluoride binding in dimer. A low binding anion as a competing anion was used to find the binding constant of chloride (Bromide as the competing anion) and fluoride (chloride as the competing anion) by competitive addition experiment.

$$\mathrm{D} + \mathrm{Br}^- \rightleftharpoons \mathrm{D} \cdot \mathrm{Br}^- \quad (6)$$

$$K_{\mathrm{Br}^-} = \frac{c_{\mathrm{D \cdot Br}^-}}{c_{\mathrm{D}} \times c_{\mathrm{Br}^-}} \quad (7)$$

Here, K_{Br^-} is the binding constant corresponding to bromide binding by dimers. $c_{D \cdot Br^-}$, c_D and c_{Br^-} are the concentrations of dimer·Br^-, free dimer, and free bromide respectively at equilibrium. Due to the slow exchange between all the species (D·Br^-, D, Br^-) in equilibrium, concentrations of each species were evaluated by integrating corresponding signals in ^{1}H NMR spectra. With the equilibrium concentrations of each species, K_{Br^-} was calculated according to equation (7). With K_{Br^-} in hand, a competitive binding experiment was performed to calculate the binding constant of chloride in dimers (K_{Cl^-}).

For a competitive binding experiment, Cl^- is added to the dimer solution followed by addition of a large amount of Br^- to observe both [**1**·(Cl^-)·**1**] and [**1**·(Br^-)·**1**] in the ^{1}H NMR spectra. Concentration of each species was further evaluated by integration of the corresponding ^{1}H NMR signals and used in equation (10) to obtain K_{Cl^-}. With K_{Cl^-} in hand, a similar competition binding experiment (with chloride (as TBACl) as the competitive anion) was performed to calculate the binding constant of fluoride in dimers (K_{F^-}). Binding constants were evaluated within 1 h of addition of halides.

$$\mathrm{D\cdot Cl^- + Br^- \rightleftharpoons D + Cl^- + Br^- \rightleftharpoons D\cdot Br^- + Cl^-} \tag{8}$$

$$\frac{c_{\mathrm{D\cdot Br^-}}}{c_{\mathrm{D\cdot Cl^-}}} = \frac{K_{\mathrm{Br^-}} \times c_{\mathrm{D}} \times c_{\mathrm{Br^-}}}{K_{\mathrm{Cl^-}} \times c_{\mathrm{D}} \times c_{\mathrm{Cl^-}}} = \frac{K_{\mathrm{Br^-}} \times c_{\mathrm{Br^-}}}{K_{\mathrm{Cl^-}} \times c_{\mathrm{Cl^-}}} \tag{9}$$

$$K_{\mathrm{Cl^-}} = \frac{K_{\mathrm{Br^-}} \times c_{\mathrm{Br^-}} \times c_{\mathrm{D\cdot Cl^-}}}{c_{\mathrm{D\cdot Br^-}} \times c_{\mathrm{Cl^-}}} \tag{10}$$

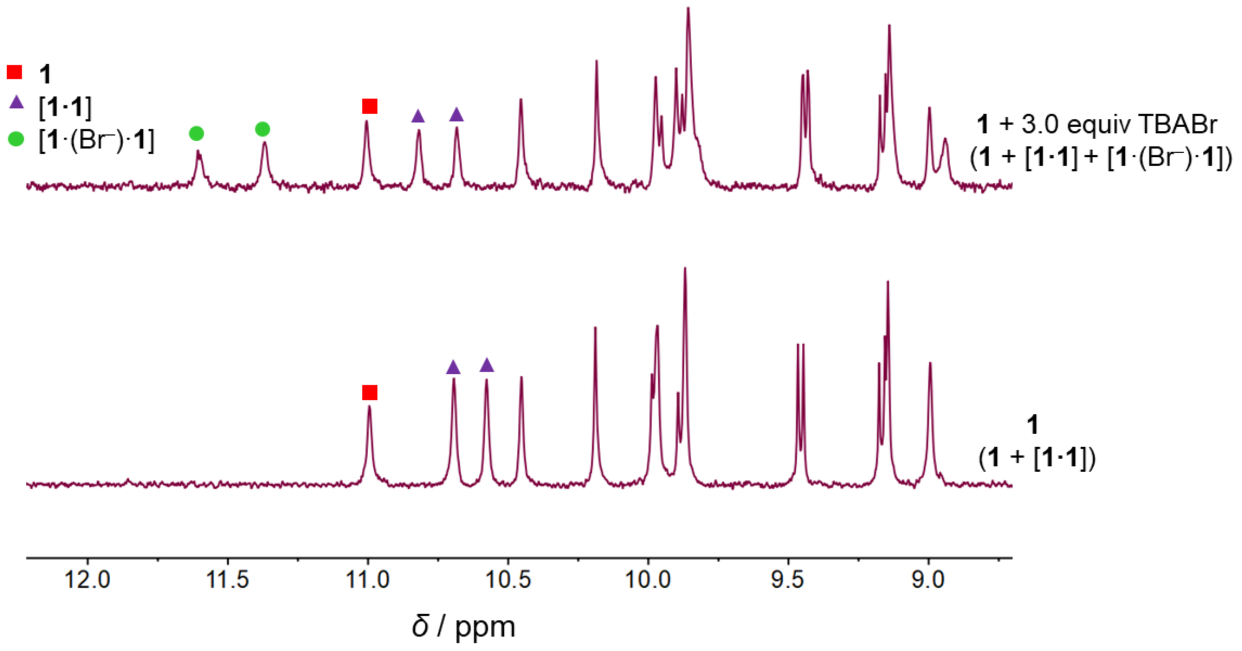

Supplementary Figure 4. ^{1}H NMR spectra of **1** and TBABr ((c(**1**) = 2.6×10^{-4} M), c(TBABr) = 3 × c(**1**), 295 K, 400 MHz) in 1:1 Tol-d_8:MeCN-d_3 showing the formation of [**1**·(Br^-)·**1**] and [**1**·**1**]. Individual spectrum of [**1**·**1**] is also shown. Only the protons in the single benzene hole are marked for clarity. A binding constant of $K_{Br^-} = (1.2 \pm 0.4)\cdot10^3$ M^{-1} was evaluated from the binding experiment.

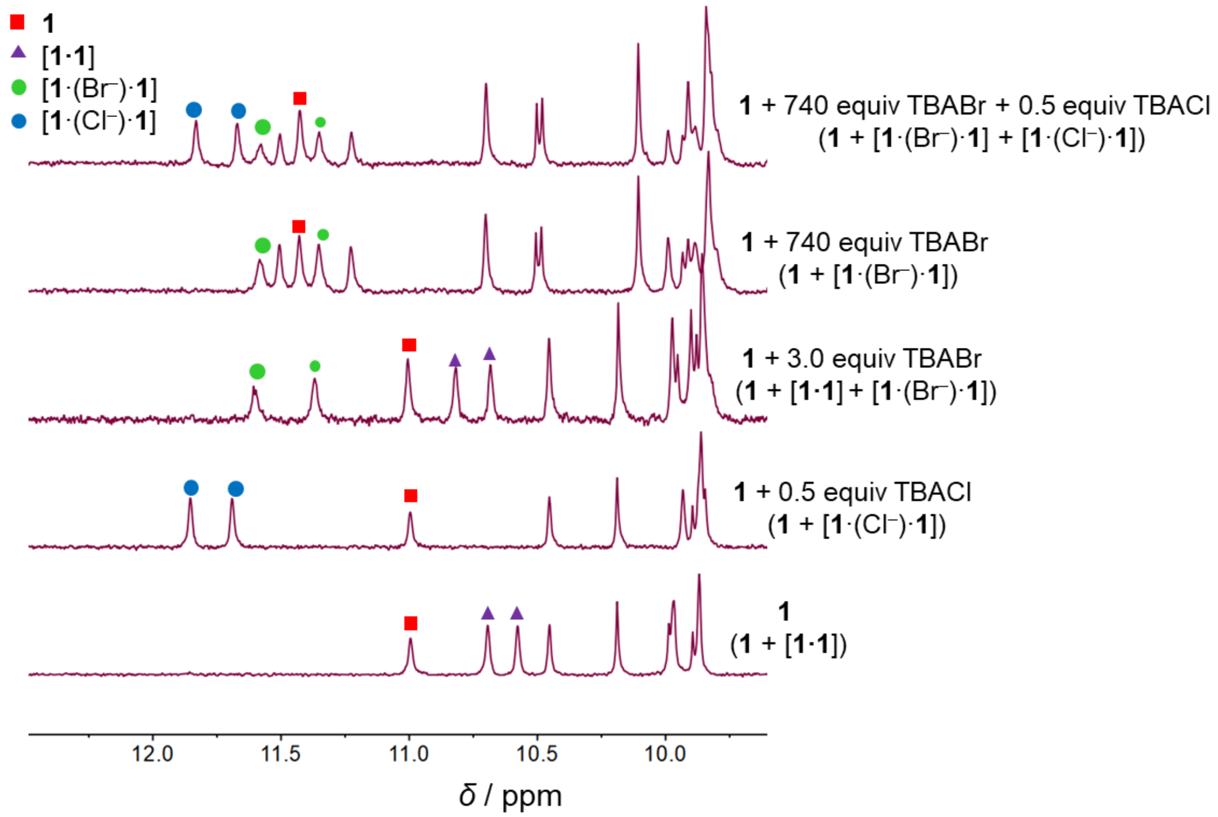

Supplementary Figure 5. ^{1}H NMR (c(**1**) = 2.3 × 10^{-4} M, 295 K, 400 MHz) spectra showing the competition titration of chloride (TBACl) and bromide (TBABr) with [**1**·**1**] in 1:1 Tol-d_8:MeCN-d_3. Each individual spectra of [**1**·**1**], [**1**·(Cl^-)·**1**], [**1**·(Br^-)·**1**] are also shown. Only the protons in the single benzene hole are marked for clarity. Some extra downfield signals seen on addition of large excess of TBABr could be due to additional binding of bromides to other protons in the nanographene **1**. A binding constant of K_{Cl^-} = (1.0 ± 0.3)·10^{7} M^{-1} was evaluated from the competition binding experiment.

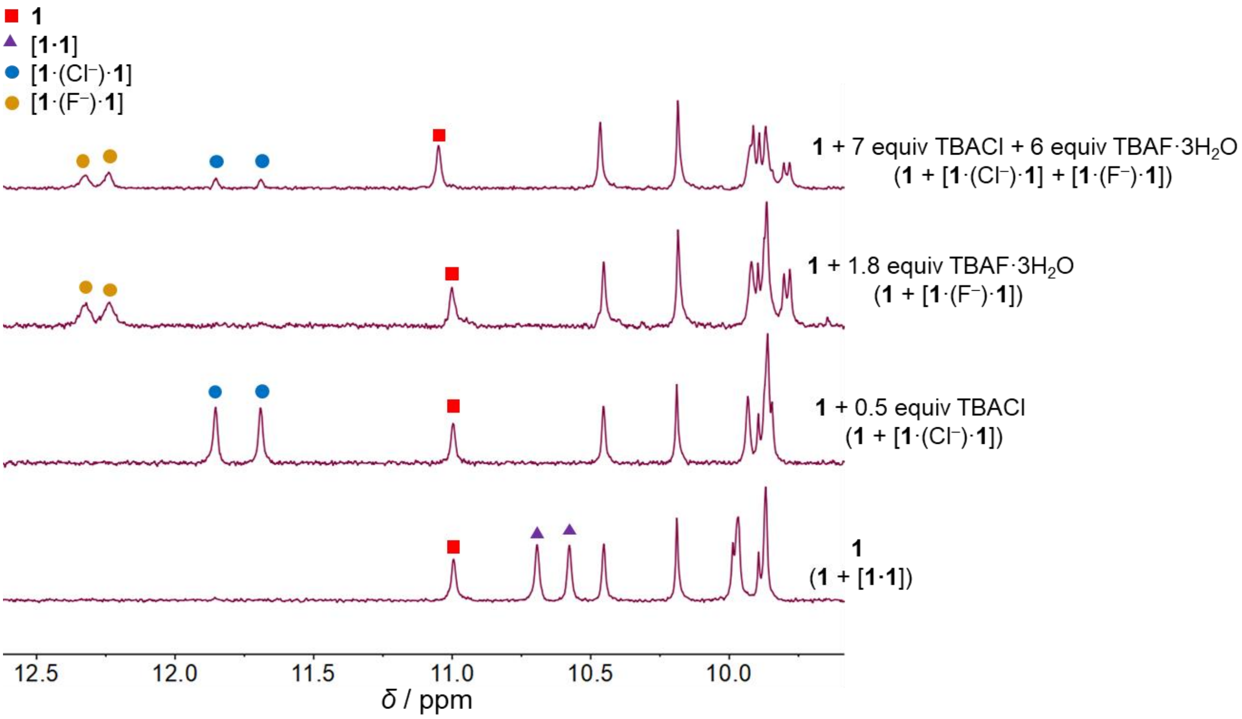

Supplementary Figure 6. ^{1}H NMR (c(**1**) = 2.4 × 10^{-4} M, 295 K, 400 MHz) spectra showing the competition titration of fluoride (tetra-n-butylammonium fluoride trihydrate, TBAF·3H_2O) and chloride (TBACl) with [**1**·**1**] in 1:1 Tol-d_8:MeCN-d_3. Each individual spectra of **1**, [**1**·**1**], [**1**·(F^-)·**1**], [**1**·(Cl^-)·**1**] are also shown. Only the protons in the single benzene hole are marked for clarity. A binding constant of K_{F^-} = (6.8 ± 1.5)·10^7 M^{-1} was evaluated from the competition binding experiment.

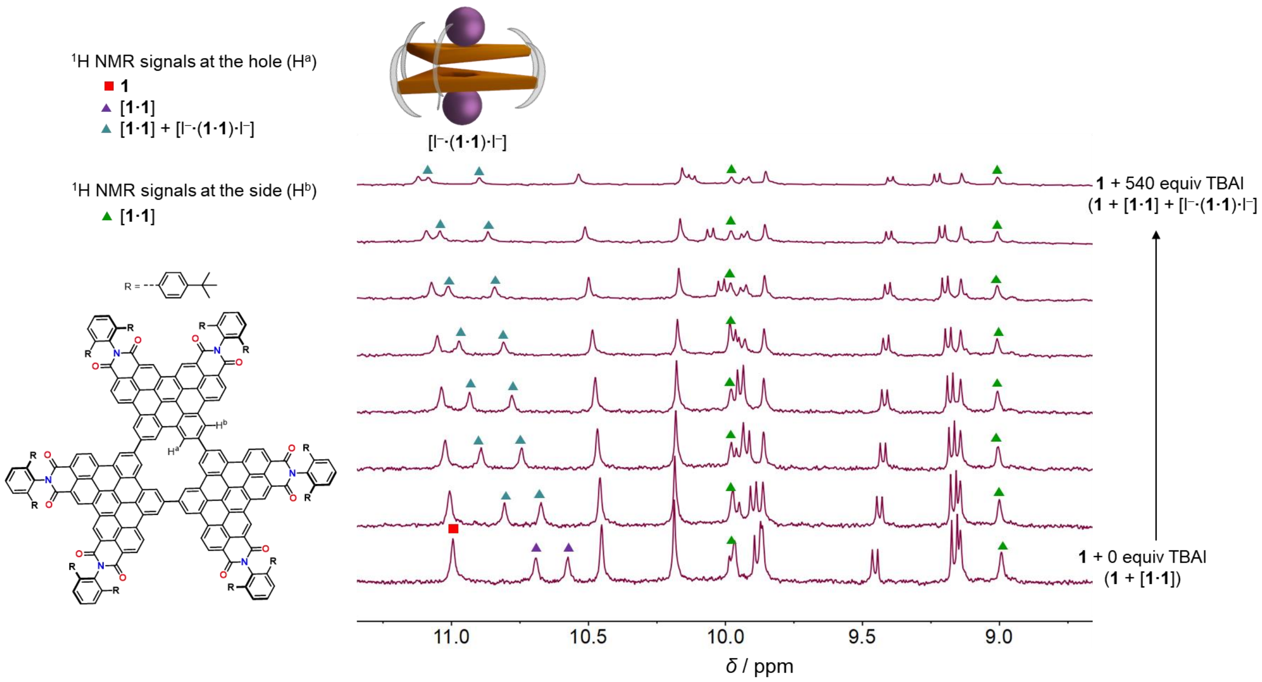

Supplementary Figure 7. ^{1}H NMR (c(**1**) ≈ 2.3 × 10^{-4} M, 295 K, 400 MHz) spectra showing the titration of iodide (tetra-n-butylammonium iodide, TBAI) with [**1**·**1**] in 1:1 Tol-d_8:MeCN-d_3. The protons located in the benzene hole (H^a) shift upon addition on TBAI, while the protons outside of this hole (H^b) remain unaffected. The shifting of ^{1}H NMR signals of the protons in the hole indicates a weak binding of I^- outside of the dimer's cavity to form complex of the type [I^-·(**1**·**1**)·I^-] which is also observed in the crystal structure. If I^- were to

bind inside of the cavity formed by single benzene hole, a new set of signals would be expected that are in slow exchange with the free dimers ([**1**·**1**]) due to the higher barrier to exchange. Here, no additional signals corresponding to the hypothetical complex [**1**·(I^-)·**1**] were observed, even upon addition of a large excess of TBAI. This indicates that the I^- is impermeable through the single benzene hole in [**1**·**1**]. Moreover, no shift in the signals for the protons outside of the hole (H^b) were observed, indicating that the iodide does not bind on the side.

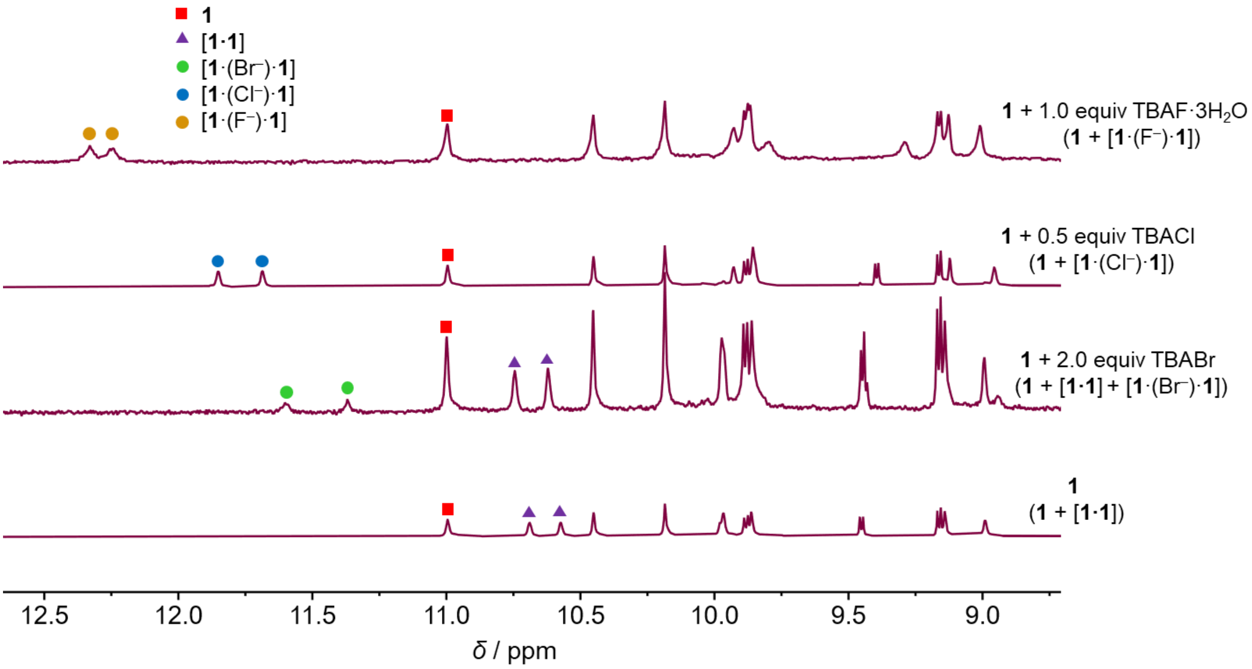

Supplementary Figure 8. Stacked ^{1}H NMR (c(**1**) = 1.5×10^{-4} M, 295 K, 600 MHz, 1:1 Tol-d_8:MeCN-d_3) spectra showing shift in the proton signals between free dimer and halide bound dimers. Only the protons in the single benzene hole are marked for clarity. All spectra with halide binding were measured immediately after addition of halide to the dimer solution.

Supplementary Table 5. Rate constant of dimerization of **1** at different temperatures in Tol:MeCN (1:1).

**Temperature (K)**	$\boldsymbol{k_d}$ **($M^{-1}s^{-1}$)**[a]
295	0.138
303	0.370
313	0.968
323	2.907

[a]k_d was calculated by fitting second order rate equation on UV/Vis absorption data and error was obtained from the non-linear fit.

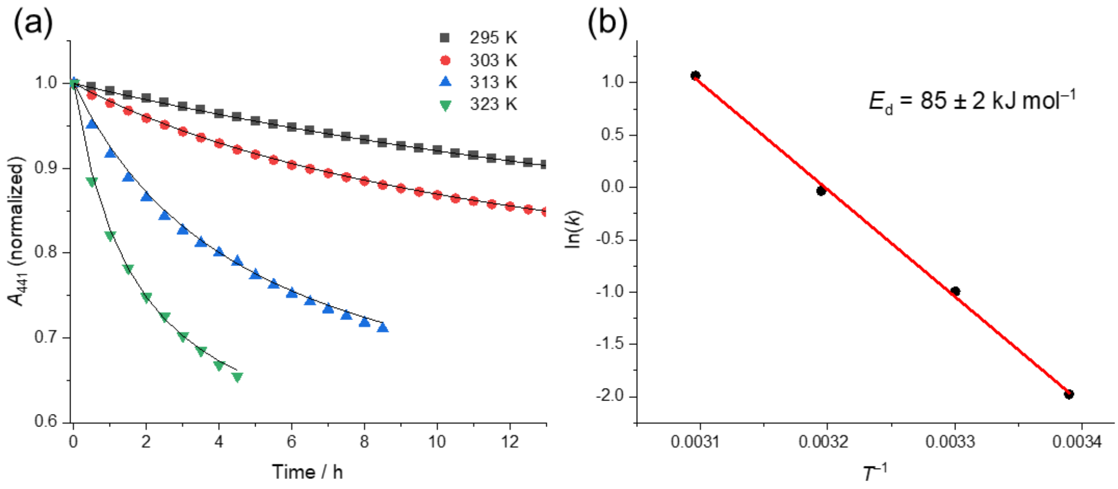

Supplementary Figure 9. (a) Time dependant dimerization of **1** at different temperatures (c(**1**) ~ 5×10^{-5} M, 1:1 Tol:MeCN) with second order kinetics fit. (b) Plot of rate constant at different temperatures. A linear fit of ln(k) to 1/T gives the activation energy (E_d) from the slope according to Arrhenius equation (3).

6 2D EXSY NMR experiments

All 2D EXSY experiments with the bromide and chloride complex were carried out after sample equilibration for 20 h (1:1 Tol-*d*$_8$:MeCN-*d*$_3$) at 600 MHz with a $^{13}C/^{1}H$ cryoprobe. First, we measured the ^{1}H spin-lattice relaxation times T_1 individually for each sample and each temperature using the inversion recovery pulse sequence (Bruker pulse program t1ir) with a recycle delay of minimum 20 s. EXSY spectra were recorded with the Bruker pulse sequence noesygpphpp[8] at 295, 323, 333 and 343 K for mixing times τ_m between 3 and 1250 ms. All EXSY data used for quantitative evaluation were acquired with recycle delays of at least 5 × T_1 of the protons in the single benzene holes. In the middle of the mixing time τ_m, the mentioned pulse sequence contains a 180° radio frequency pulse flanked by two pulsed magnetic field gradients of opposite sign (bipolar gradient pulse pair). This building block prevents the use of a mixing time of zero (we used 3 ms as the shortest τ_m value for this pulse sequence). Since the subsequent evaluation of the EXSY data with the EXSYCALC software (available at https://mestrelab.com/software/freeware/) requires a reference experiment with zero or very short mixing time and to perform a general cross-check of our EXSY data, we recorded additional EXSY spectra for one bromide sample at 343 K for mixing times of 0, 200 and 750 ms with the pulse sequence noesyphpp. It corresponds to noesygpphpp with the exception that the 180° RF pulse and the two pulsed magnetic field gradients during the mixing time are missing and thus an EXSY spectrum with τ_m = 0 could be acquired. The quantitative evaluation of the EXSY spectra recorded with the pulse sequence noesyphpp yielded rate constants that correspond to the pulse sequence noesygpphpp. Therefore, only the data acquired with the latter will be presented and discussed in the following section.

We have chosen the protons in the single benzene hole to determine rate constants from the ^{1}H–^{1}H EXSY measurements. At 295 K, 2D ^{1}H–^{1}H EXSY spectra show very low intensity exchange cross-signals between protons in the single benzene hole of [**1**·**1**] and [**1**·(Br$^-$)·**1**] (c(**1**) = 1.5 × 10^{-4} M, c(TBABr) = 5.6 × 10^{-4} M), both for short and long mixing times (EXSY spectra were recorded for τ_m values between 50 and 1250 ms at this temperature). Thus, we increased the temperature to 323 K to get higher quality data (higher temperature would increase the exchange rate and thereby increase the cross-signal intensities). At 323 K (Supplementary Figure 10) for mixing times of 200 ms and 950 ms, each of the two proton signals in the single benzene hole of [**1**·**1**] shows an exchange cross signal with the corresponding proton in [**1**·(Br$^-$)·**1**] (c(**1**) = 1.5 × 10^{-4} M, c(TBABr) = 1.5 × 10^{-4} M). However, as compared to τ_m = 200 ms, the EXSY spectrum with τ_m = 950 ms contains additional cross signals that connect the abovementioned exchange cross peaks with each other. We attribute these additional cross signals at the long mixing time to a combined exchange/NOE effect: After a proton has performed an exchange process, it stays in spatial proximity with adjacent inner protons in the single benzene hole of the other species. For determining the rate constants from the intensities of diagonal and cross signals, an optimum mixing time can be estimated according to $\tau_{m,opt} \approx 1/(T_1^{-1} + k_{+Br}^{M} + k_{-Br}^{M})$,[9, 10] where k_{+Br}^{M} and k_{-Br}^{M} are the magnetization rate constants. Since the latter are unknown, but small as compared to T_1^{-1}, we used a mixing time of ≈ 90% of the average T_1 values, resulting in 950 ms for 323 K. A full relaxation matrix analysis[11, 12] using the EXSYCALC software was performed to obtain the

magnetization rate constants which were converted to chemical exchange rates. We also used τ_m = 200 ms to evaluate the rate constants. Since the magnetization rates calculated from these two mixing times are very similar, we concluded that the additional cross signals that arise at the long mixing time might not be influencing the evaluated signal intensities. We also measured EXSY spectra at 333 K (c(**1**) = 1.5 × 10^{-4} M, c(TBABr) = 1.6 × 10^{-4} M) and 343 K (c(**1**) = 1.5 × 10^{-4} M, c(TBABr) = 1.5 × 10^{-4} M). Their evaluation allowed us to estimate the activation energy according to Arrhenius equation (3). The results are summarized in Supplementary Table 6.

To examine if exchange exists (on the timescale detectable by EXSY experiments) for the chloride complex, we investigated a sample (1:1 Tol–d_8:MeCN–d_3) with c(**1**) = 2.0 × 10^{-4} M and c(TBACl) = 4 × 10^{-5} M (i. e. 0.5 equiv. chloride with respect to the dimer concentration, assuming 80% dimer formation) at 343 K. As expected, an EXSY spectrum with a mixing time of 800 ms (95% of the average T_1 values of the four signals of the protons in the single benzene hole) did not show any of those exchange signals discussed above for the bromide bound dimer.

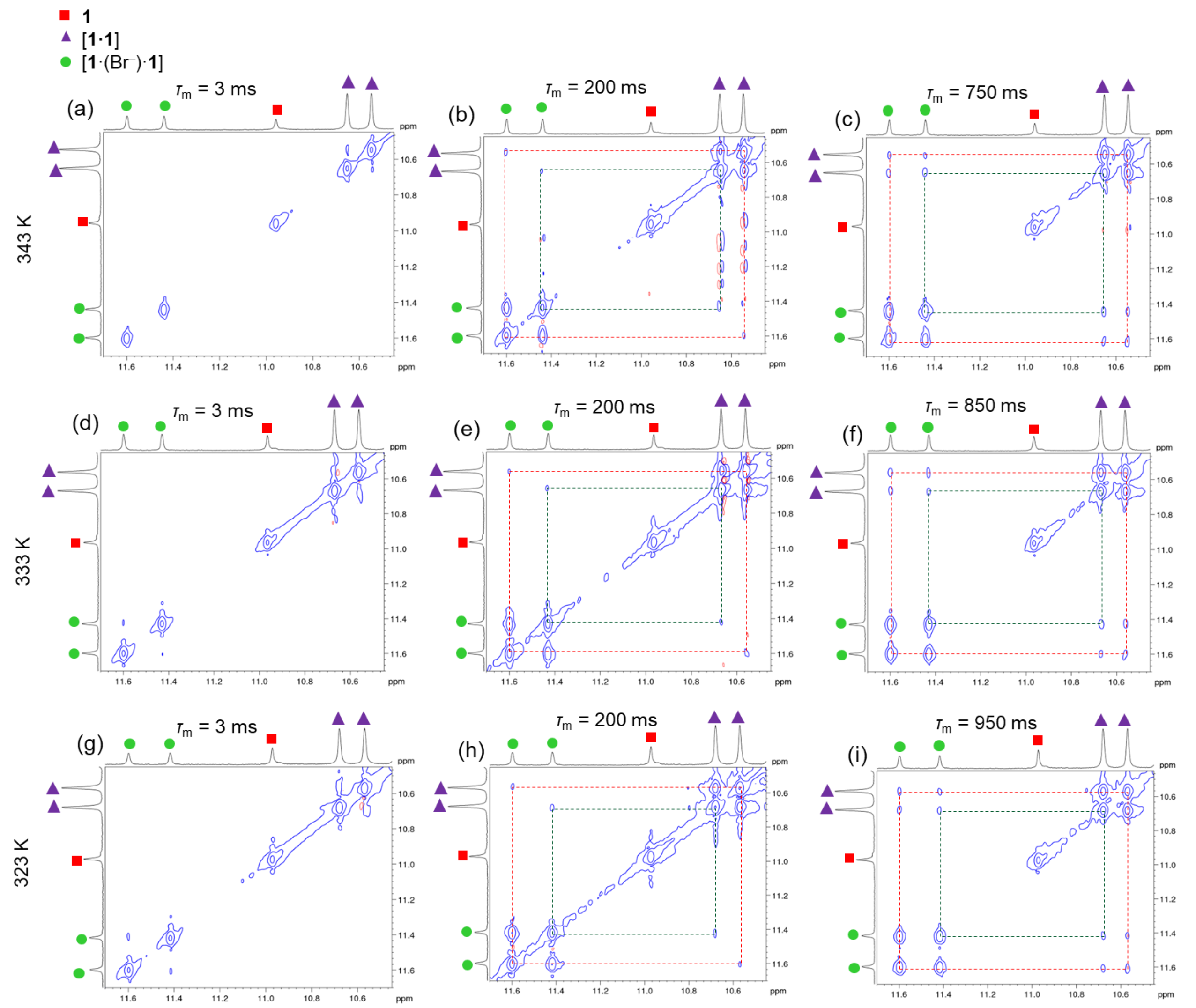

Supplementary Figure 10. Excerpts from 2D EXSY spectra (showing separate signals for **1**, [**1**·**1**] and [**1**·(Br$^-$)·**1**]) measured at three different temperatures for the evaluation of activation energy (600 MHz, 1:1 Tol-d_8:MeCN-d_3). 2D EXSY at 343 K (c(**1**) = 1.5 × 10^{-4} M, c(TBABr) = 1.5 × 10^{-4} M) for mixing times τ_m of (a) 3 ms, (b) 200 ms, (c) 750 ms. 2D EXSY at 333 K (c(**1**) = 1.5 × 10^{-4} M, c(TBABr) = 1.6 × 10^{-4} M) for mixing times τ_m of (d) 3 ms, (e) 200 ms, (f) 850 ms. 2D EXSY at 323 K (c(**1**) = 1.5 × 10^{-4} M, c(TBABr) = 1.5 × 10^{-4} M) for mixing times τ_m of (g) 3 ms, (h) 200 ms, (i) 950 ms. With the exception of τ_m = 3 ms, exchange signal between [**1**·**1**] and [**1**·(Br$^-$)·**1**] can be seen for all mixing times.

Supplementary Table 6. Experimental rate constants calculated for bromide binding from 2D EXSY measurements at 343 K, 333 K and 323 K.

Temperature (K)	Mixing time (τ_m)	k^M_{+Br} (s^{-1})	k^M_{-Br} (s^{-1})	k_{+Br} ($M^{-1}s^{-1}$)	k_{-Br} (s^{-1})
343[a]	200 ms	0.034	0.106	223	0.106
	750 ms	0.027	0.090	184	0.090
333[b]	200 ms	0.021	0.081	131	0.081
	850 ms	0.022	0.073	137	0.073
323[c]	200 ms	0.018	0.053	121	0.053
	950 ms	0.015	0.055	101	0.055

[a]Experimental conditions: 600 MHz, 1:1 Tol-d_8:MeCN-d_3, c(**1**) = 1.5 × 10^{-4} M, c(TBABr) = 1.5 × 10^{-4} M. [b]Experimental conditions: 600 MHz, 1:1 Tol-d_8:MeCN-d_3, c(**1**) = 1.5 × 10^{-4} M, c(TBABr) = 1.6 × 10^{-4} M. [c]Experimental conditions: 600 MHz, 1:1 Tol-d_8:MeCN-d_3, c(**1**) = 1.5 × 10^{-4} M, c(TBABr) = 1.5 × 10^{-4} M. Diagonal and cross peak intensities obtained from 2D EXSY measurements were evaluated using the EXSYCALC software to obtain magnetization rate constants. k^M_{+Br} and k^M_{-Br} are forward and reverse magnetization rate constants, while k_{+Br} and k_{-Br} are chemical rate constants. Forward chemical rate constants were calculated according $k_{+Br} = k^M_{+Br} / c(\text{TBABr})$ and reverse chemical rate constants were calculated according to $k_{-Br} = k^M_{-Br}$.

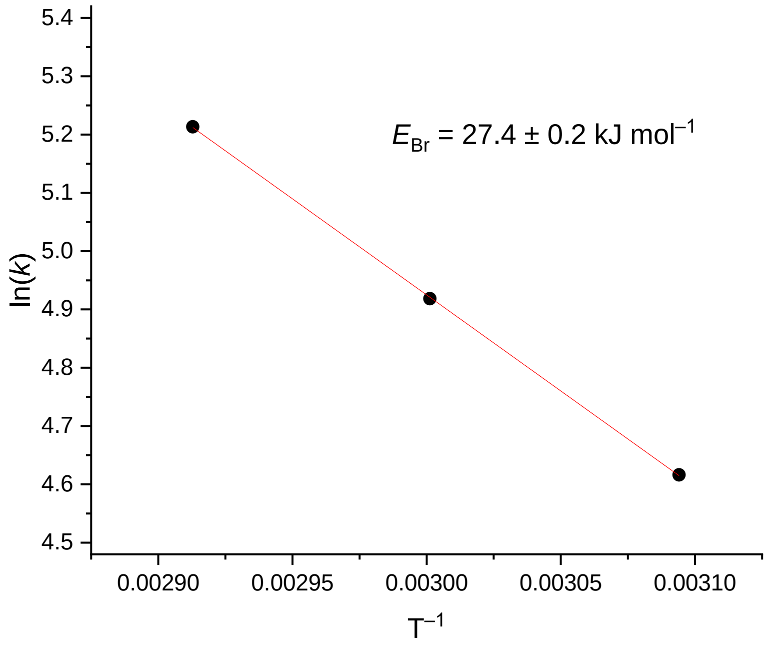

Supplementary Figure 11. Plot of rate constants (k_{+Br}) at different temperatures obtained from 2D EXSY experiments at the longest mixing time. A linear fit of ln(k) vs. 1/T gives the activation energy (E_{Br}) from the slope according to Arrhenius equation (3). Error shown is the error obtained from the linear regression analysis.

7 Theoretical Calculations

Computational Methods

Pore diameter in the bilayer holey nanographene was calculated using pywindow[13] and the subnanometer channel was constructed using molovol program (two probes mode, small probe: 0.5 Å, large probe: 5 Å).[14] Nudged Elastic Band - Climbing Image (NEB-CI) method was used to estimate the energy barrier of halide binding in the bilayer nanographene. NEB-CI was performed using semi-empirical GFN2-xTB level of theory as the size of bilayer nanographene was prohibitive to do higher level DFT calculations. NEB trajectory shows an almost barrierless transition for fluoride, while chloride and bromide shows high barriers. The calculated barrier energies are higher than the experimental barrier energy obtained for bromide binding. This is expected as the calculation is semi-empirical and does not include entropic contribution and solvation effects. Nevertheless, a qualitative picture of increasing barrier with increasing size of halide is clearly seen in the NEB calculations. For chloride complex, a transition state calculation was also performed from the converged structure obtained from NEB-CI calculation.

For iodide, the optimized structures (GFN2-xTB) show that [**1**·**1**] structure is more stable than [**1**·(I^-)·**1**] by 51 kJ mol^{-1} and thus supporting the experimental observation that iodide does not bind inside [**1**·**1**] even at large excess of TBAI.

Programs used

ALMO-EDA calculations were performed using Q-Chem 5.1[15] with B3LYP-D3/ 6-311G(d) level of theory. NEB-CI calculations were performed using ORCA 5.0.[16] Semiempirical GFN2-xTB calculations were performed using xtb program.[17]

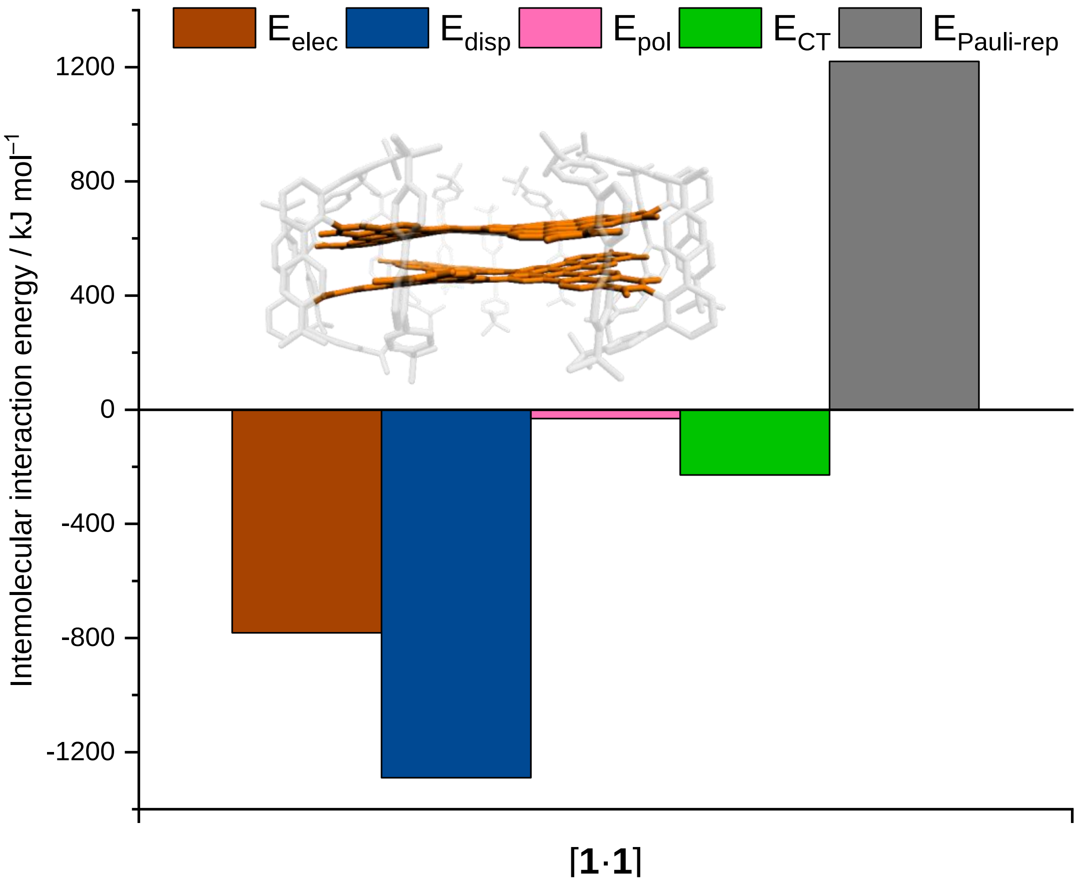

Supplementary Figure 12. Bar plot showing ALMO-EDA of crystal structure of [**1**·**1**].

Supplementary Table 7. ALMO-EDA interaction energies between monomers in dimer [**1**·**1**]. Absolute values of the plot in Supplementary Figure 12.

structures	E_{total} (10^2 kJ·mol^{-1})[a]	E_{elec} (10^2 kJ·mol^{-1})[b]	E_{disp} (10^2 kJ·mol^{-1})[c]	E_{pol} (10^2 kJ·mol^{-1})[d]	E_{CT} (10^2 kJ·mol^{-1})[e]	$E_{Pauli\text{-}rep}$ (10^2 kJ·mol^{-1})[f]
[**1**·**1**][g]	-11.1	-7.8	-13.0	-0.31	-2.3	12.2

[a]Total interaction energy. [b]Electrostatic component. [c]Dispersion component. [d]Polarization component. [e]Charge-transfer component. [f]Pauli-repulsion component. [g]EDA done on crystal structures.

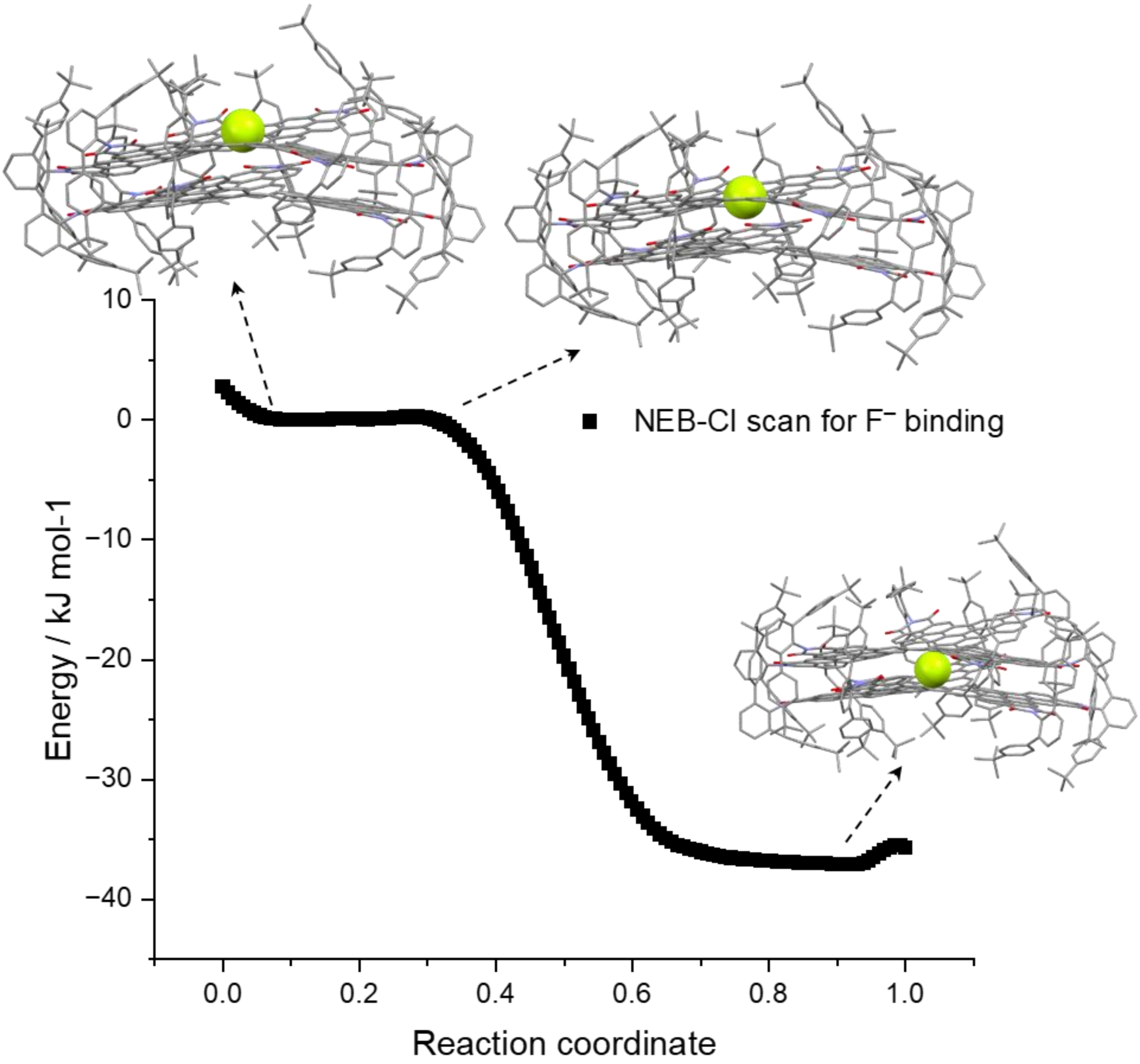

Supplementary Figure 13. Computed NEB-CI trajectory of F^- binding to [**1**·**1**]. Relevant structures are also shown. NEB calculations show that F^- binding to [**1**·**1**] is almost barrier free. Computed energy includes only electronic energy.

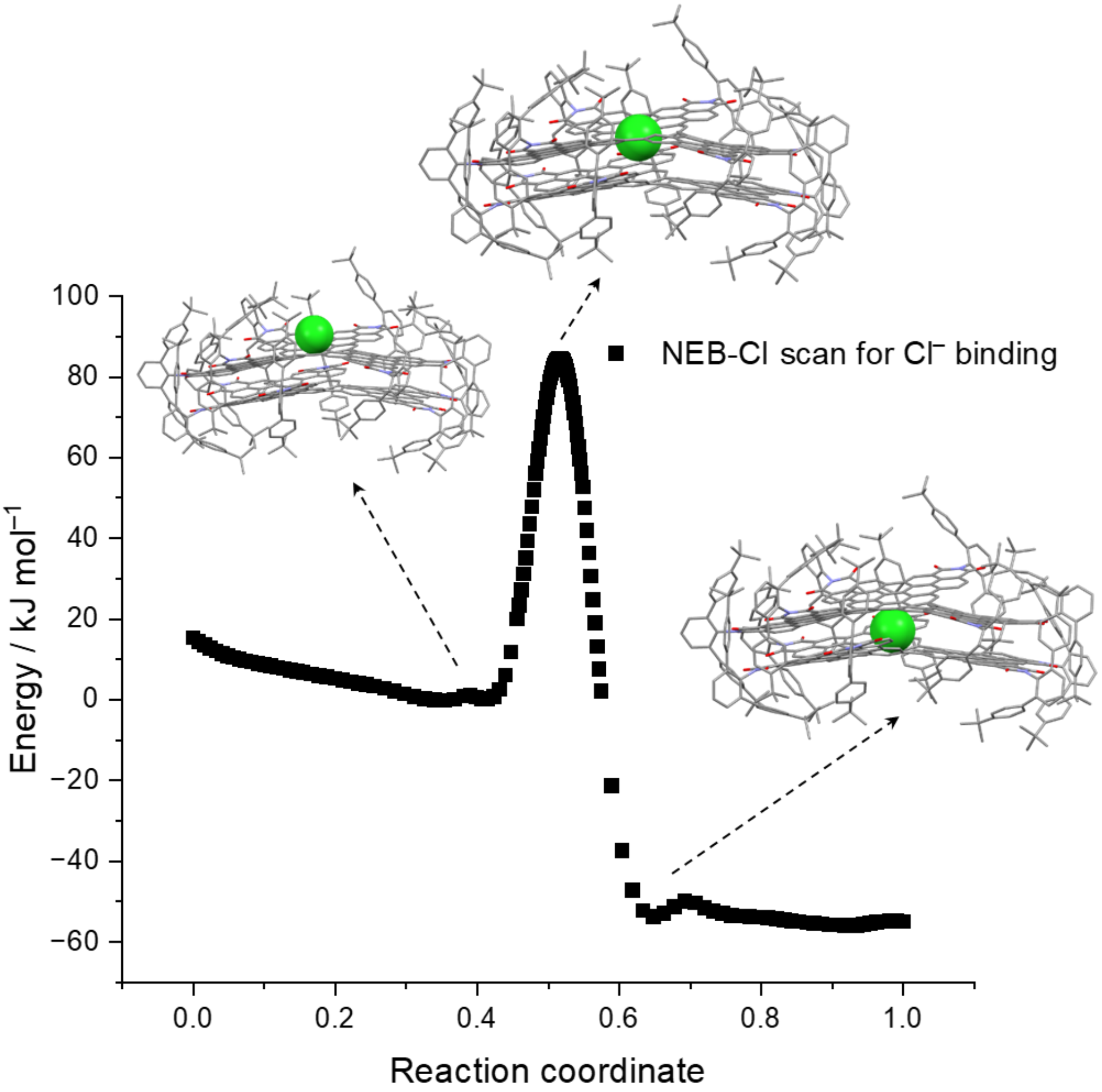

Supplementary Figure 14. Computed NEB-CI trajectory of Cl^- binding to [**1**·**1**]. Relevant structures are also shown. NEB calculations show that Cl^- binding to [**1**·**1**] has a barrier of 85 kJ mol^{-1}. Computed energy includes only electronic energy.

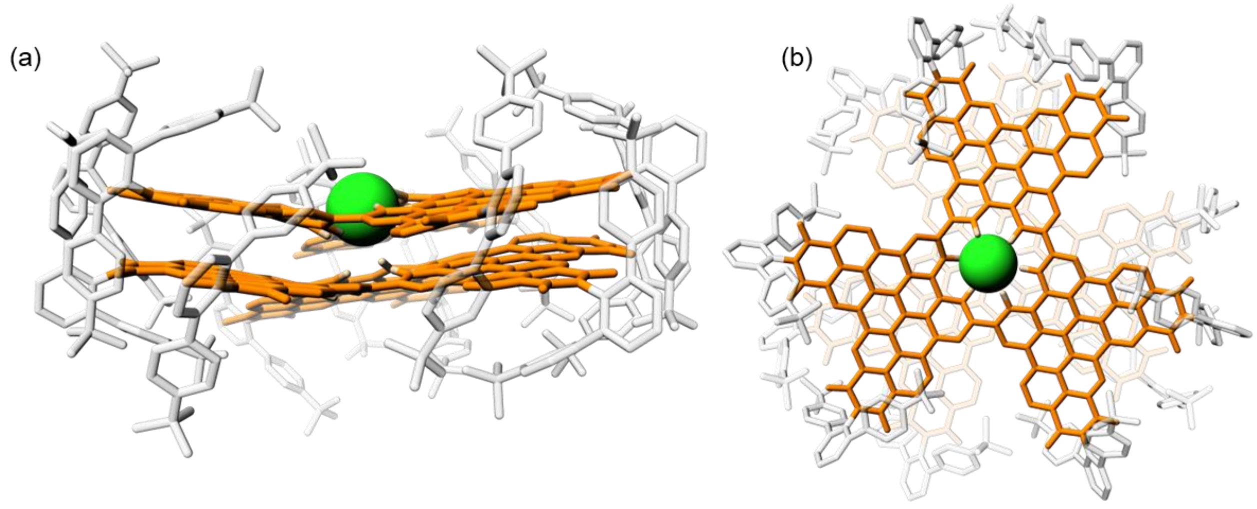

Supplementary Figure 15. Calculated transition state of chloride binding in the bilayer nanographene. (a) Side view showing the occupancy of chloride at the centre of single benzene hole. (b) Top view showing the distortion of C–H hydrogens at the transition state.

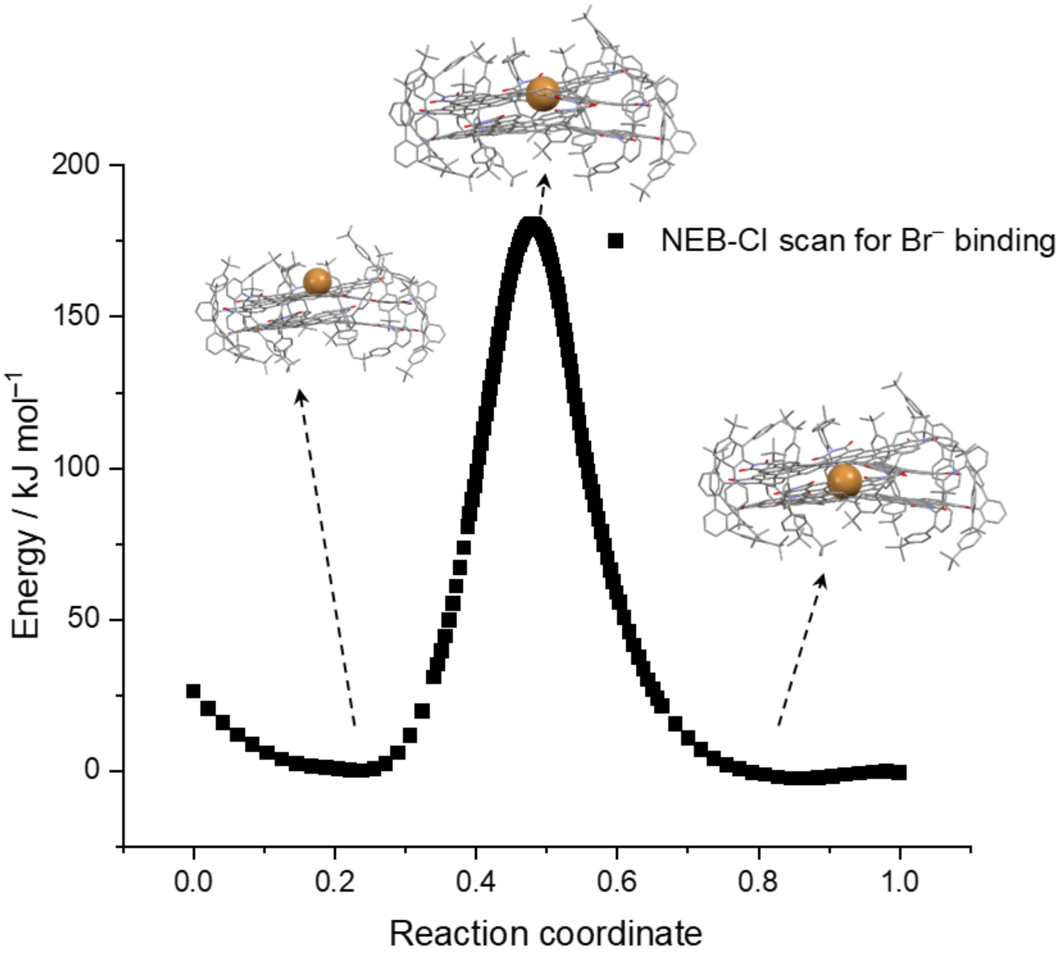

Supplementary Figure 16. Computed NEB-CI trajectory of Br^- binding to [**1**·**1**]. Relevant structures are also shown. NEB calculations show that Br^- binding to [**1**·**1**] has a barrier of 180 kJ mol^{-1}. Computed energy includes only electronic energy.

8 Alternate mechanisms

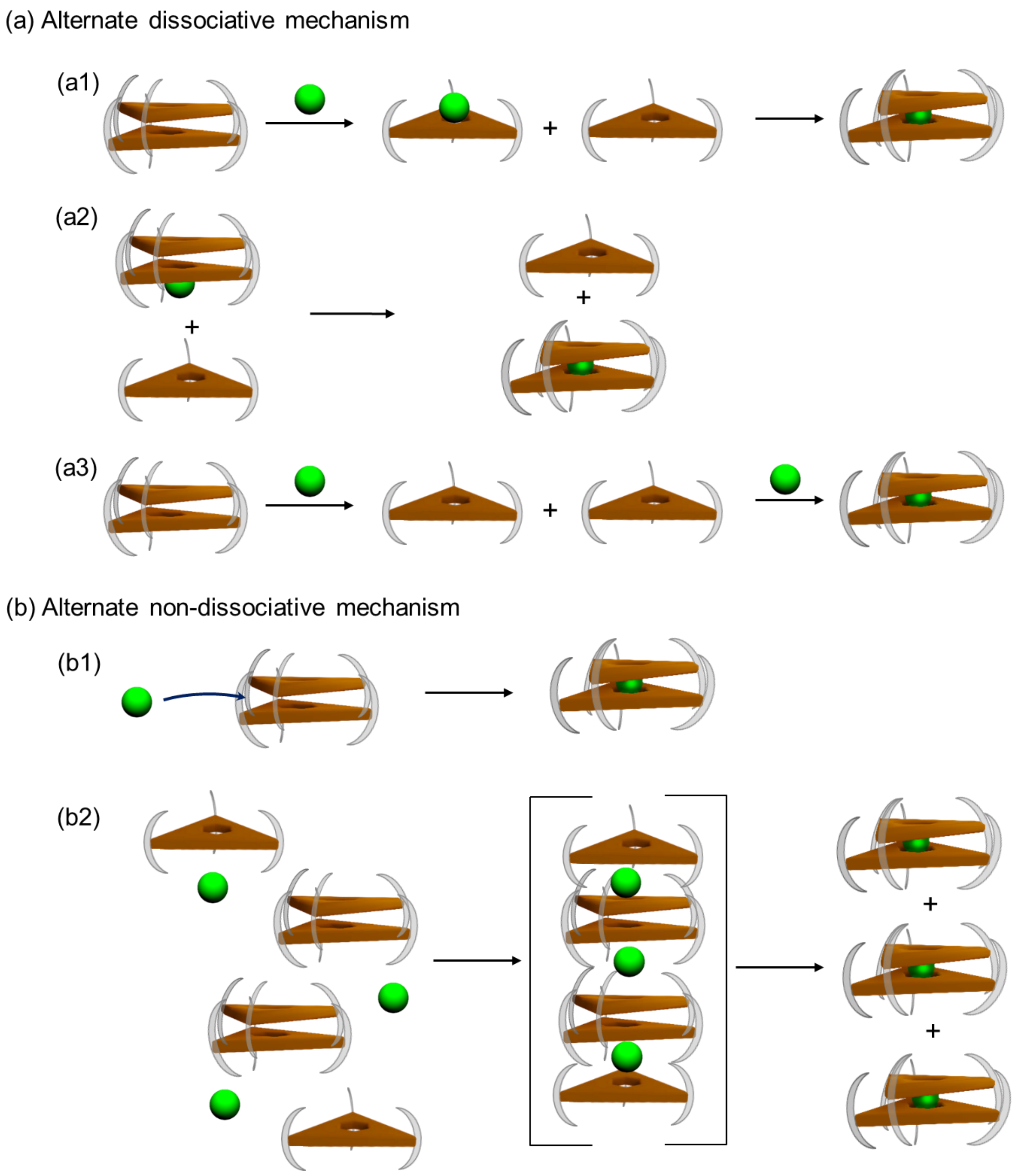

Supplementary Figure 17. Alternate mechanistic scenarios that we considered. (a) Alternate dissociative mechanisms. (a1) Dissociation of [**1**·**1**] into monomers and forming monomer-halide ([**1**·X^-]) intermediates. (a2) Involvement of monomeric **1** left in the solution to assist in forming [**1**·(X^-)·**1**] through [**1**·**1**]·X^- intermediate. (a3) Halide exchange caused by a dissociative mechanism assisted by the halide. (b) Non-dissociative mechanisms. (b1) Lateral insertion of halide in the dimer. (b2) Structural rearrangement of higher-order assemblies without converting to monomers. Figure shows only one representative schematic of several possible assemblies involving multiple dimer-complexes. Both dissociative and non-dissociative mechanisms are discarded based on the experimental observations and theoretical analysis detailed below.

Here we discuss the alternate mechanisms we considered for the observed halide binding.

Alternate dissociative mechanism

1. Dissociation of [1·1] into monomers and forming monomer-halide ([1·X$^-$]) intermediates (Supplementary Figure 17a1)

We did not see any evidence for the existence of [**1**·X$^-$] during complexation. We discarded this mechanism due to following reasons. Halide was added after the dimer formation and if the dimer dissociates in monomer, that will take several hours (half lifetime of dimerization is 6.6 h at 2.8×10^{-4} M, so the dissociation would be even longer) and thus we would not see an immediate binding. Furthermore, after halide addition, we do not see any increase in the concentration of monomers. Since we see an immediate binding of halides to [**1**·**1**], we concluded that there is no dissociation due to halide addition.

2. Involvement of monomeric 1 left in the solution to assist in forming [1·(X$^-$)·1] through [1·1]·X$^-$ intermediate (Supplementary Figure 17a2)

If this mechanism is true, a fast formation of [**1**·(X$^-$)·**1**] should happen if we add halides already with monomeric **1** and measured time-dependent dimerization. But we do not see any speeding up of dimerization by addition of halides. Identical dimerization rate constant (Figure 2d) and apparent rate constant obtained with 1 equiv of TBACl (Supplementary Figure 18) indicate that monomer is not involved in the halide binding mechanism. Thus, we discarded the alternate mechanism of involvement of monomer in assisting the formation of [**1**·(X$^-$)·**1**].

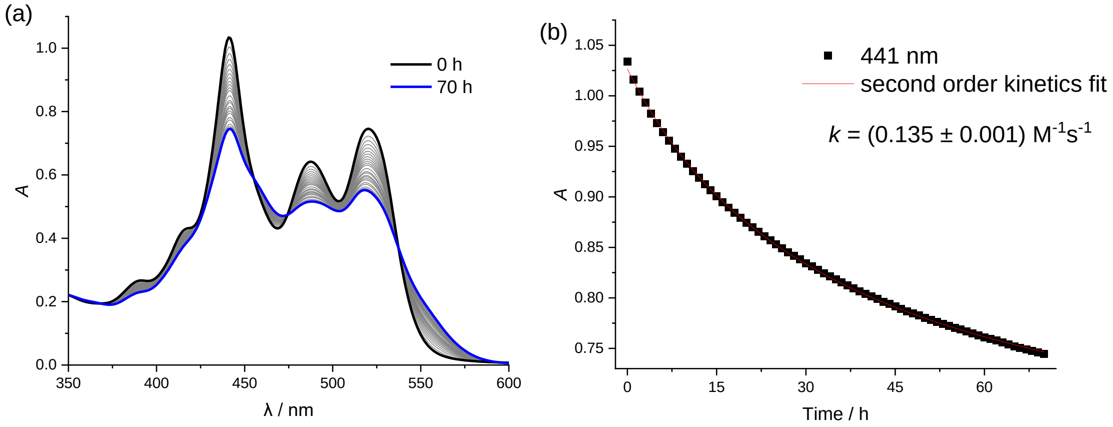

Supplementary Figure 18. (a) Time dependant UV/Vis absorption spectra with of **1** and 1 equiv TBACl (c(**1**) = 5.7×10^{-5} M, 295 K)) showing change in spectra with time indicating dimerization. (b) Time-dependent absorbance at 441 nm with second order kinetics fit.

3. Halide exchange caused by a dissociative mechanism assisted by the halide (Supplementary Figure 17a3).

A dissociative mechanism assisted by halide could cause the halide exchange we observed in EXSY experiments. Here we consider Br$^-$ as an example because of its moderate rate constant and binding

constant that allows the EXSY experiment feasible. The results should also be the same for Cl^- and F^-. If the addition of bromide causes a fast dissociation of dimer [**1**·**1**] followed by fast association of components to form [**1**·(Br^-)·**1**] complex, then the exchange signals would not correspond to a halide permeation event but instead due to a dissociation. If the exchange signals are due to a dissociation caused by halide, the exchange rate will increase with an increase in concentration of halide. Such a fast dissociative mechanism is improbable if the rate stays same or decreases (the latter could occur if a "hole-blocking effect" is active due to the excess of bromide itself (Supplementary Figure 19), *vide infra*). To test this possibility, we performed 2D EXSY on different concentrations of bromide. The resulting rate constants are given in Supplementary Table 8. EXSY experiments revealed an apparent decrease in rate constant ($k_{+\mathrm{Br}}$) on increasing concentration of TBABr that allowed us to discard the possibility of a dissociative mechanism of exchange. We attributed the decrease in exchange rate with increase in bromide concentration to a hole-blocking effect caused by the electrostatic repulsion of the permeating bromide by the externally blocked bromide. On increasing the concentration of bromide, ^{1}H NMR signals of protons at the hole (H^a) in [**1**·**1**] showed a shift (Supplementary Figure 19). Such a shift is similar to the shift observed on addition of iodide to [**1**·**1**] (Supplementary Figure 7), for which crystal structure showed the formation of [I^-·(**1**·**1**)·I^-]. Thus, we attributed the shift of protons at the hole (H^a) in [**1**·**1**] to the formation of [Br^-·(**1**·**1**)·Br^-] complex where the bromide binds outside the hole-entry and thereby is blocking the hole. However, there was no shift for the protons at the side (H^b) showing that the bromide does not bind on the sides (protons at the side (H^b) were identified from ^{1}H–^{1}H COSY experiment (Supplementary Figure 33)). Thus, a decreasing rate of halide exchange with increasing concentration of halide convincingly discards the fast dissociative mechanism assisted by halide.

Supplementary Table 8. Experimental rate constants calculated for bromide binding from 2D EXSY measurements at different concentrations of TBABr at 343 K. A decrease in chemical exchange rates with increasing concentration of TBABr discards the possibility of a fast dissociative mechanism assisted by halide.

c(TBABr)	$k_{+\mathrm{Br}}$ ($M^{-1}s^{-1}$)	$k_{-\mathrm{Br}}$ (s^{-1})
1.5×10^{-4} M	184	0.090
3.0×10^{-4} M	163	0.063
6.1×10^{-4} M	95.5	0.054
1.2×10^{-3} M	55.8	0.033

Experimental conditions: 600 MHz, 1:1 Tol-d_8:MeCN-d_3, c(**1**) = 1.5×10^{-4} M, τ_m = 750 ms, 343 K. Diagonal and cross peak intensities obtained from 2D EXSY measurements were evaluated using the EXSYCALC software to obtain magnetization rate constants ($k^M_{+\mathrm{Br}}$ and $k^M_{-\mathrm{Br}}$) which are then converted to chemical rate constants ($k_{+\mathrm{Br}}$ and $k_{-\mathrm{Br}}$). $k_{+\mathrm{Br}}$ and $k_{-\mathrm{Br}}$ corresponds to forward and reverse chemical rate constants for bromide binding in the dimer. Forward chemical rate constants were calculated according $k_{+\mathrm{Br}} = k^M_{+\mathrm{Br}}/c(\mathrm{TBABr})$ and reverse chemical rate constants were calculated according to $k_{-\mathrm{Br}} = k^M_{-\mathrm{Br}}$.

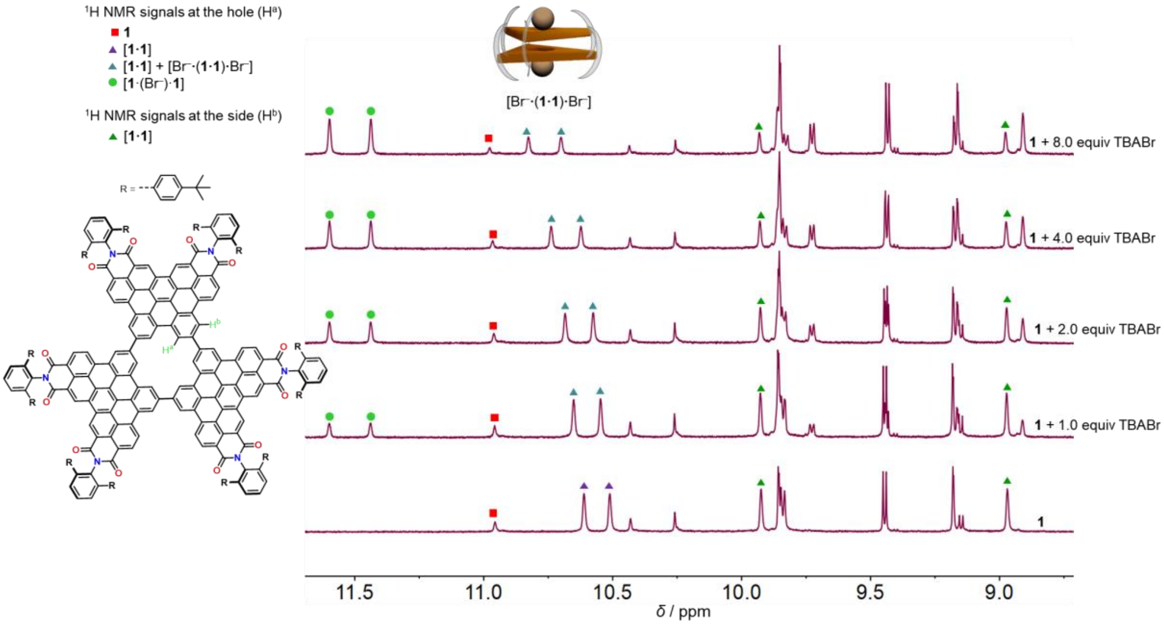

Supplementary Figure 19. ^{1}H NMR spectra **1** with increasing concentration of TBABr ((c(**1**) = 1.5×10^{-4} M), 343 K, 600 MHz, in 1:1 Tol-d_8:MeCN-d_3). A shift in the [**1**·**1**] signals indicates the blocking of hole by excess bromide and thereby forming [Br^{-}·(**1**·**1**)·Br^{-}] complex. While bromide binds on the outside of hole-entry, no such shift was observed on the side protons (H^{b}) indicating that bromide does not bind outside the lateral-entry.

Alternate non-dissociative mechanism

1. Lateral insertion of halide (Supplementary Figure 17b1)

Following theoretical and experimental analysis was used to assess the possibility of a lateral insertion of halide ion.

Theoretical analysis shows very high energy barrier for lateral entry

We have used semi-empirical GFN2-xTB method to assess the energetic barrier for the lateral entry of chloride and bromide ions, which showed a very high energetic requirement of ca. 350 and 610 kJ mol^{-1}, respectively (Supplementary Figure 20). These high energy barriers also led to an unsuccessful estimation of transition state for the lateral entry. The energies for the hypothetical scenario of lateral halide insertion are as high as or higher than the bond dissociation energy of C–C bonds (347 kJ mol^{-1}). Thus, during the calculations of reaction pathway, undesired carbon–halide bonds were formed between the aromatic surface of **1** and halide ion. In other attempts for the estimation of transition state, unrealistic geometries (high degree of bending/distortion) with extremely high barrier energies above 1000 kJ mol^{-1} were observed (we note here that for these calculations constraints were applied to disallow dissociation of dimer). Because we do not observe any dissociation/bond formation on addition of halide to dimers and indeed we see a fast binding of halides that is inconsistent with the high energetic requirement for a lateral entry, we concluded that a chloride or bromide cannot insert laterally into the dimer under the experimental conditions.

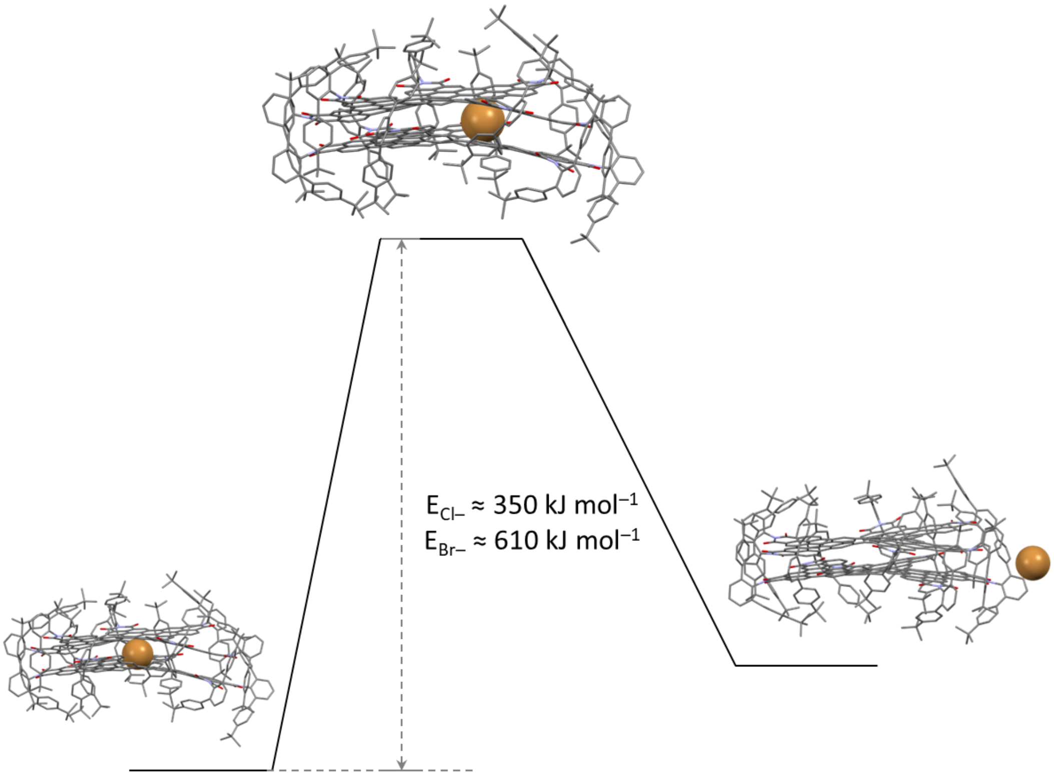

Supplementary Figure 20. Schematic representation of the estimated energetic barrier required for a non-dissociative chloride entry through the lateral side. Potential energy scan was done using GFN2-xTB method.

Experimental analysis shows a significant decrease in the rate of halide binding when the holes are blocked

We hypothesized that if the lateral entry of halide ion would be possible, the rate constant for halide binding should be unaffected when the hole is (partially) blocked from the outside. If the rate of halide binding decreases with the blocker, then an entry through the hole is likely occurring. If the rate of halide binding remains same with the blocker, a halide entry through the side (lateral entry) or any other mechanism(s) that doesn't involve the hole is plausible. A requirement for a good blocker is that it should only bind outside the hole, and it should not bind other positions especially inside the hole and outside the lateral entry (Supplementary Figure 21). Thus, we selected iodide as the blocker as it cannot bind inside and can bind outside the hole as evident from ^{1}H NMR shift of C–H protons at the hole in the dimer (Supplementary Figure 7) on increasing the iodide concentration and from X-ray single crystal structure obtained for the iodide complex of dimer ([I^{-}· (**1**· **1**)· I^{-}] (Supplementary Table 4, Supplementary Figure 3, see also discussion below for an additional iodide found for the crystal structure in the small cavity at the periphery of dimer). Moreover, there was no shift for the protons at the side (H^{b}, Supplementary Figure 7) showing that the iodide does not block the lateral entry side (Protons at the side (H^{b}) was identified from ^{1}H–^{1}H COSY experiment (Supplementary Figure 33)). Further, we performed 2D EXSY experiments to calculate the rate constant for bromide binding with varying concentration of iodide. The results are summarized in Supplementary Table 9. An apparent decrease in rate constant ($k_{+\mathrm{Br}}$) on increasing the concentration

of TBAI shows that the entry of bromide is blocked by the binding of iodide outside the hole. Thus, a reduction in exchange rate when the hole is blocked indicates that the bromide passes through the hole for binding in the cavity and rejects the possibility of lateral entry as well as other entry pathways that are not influenced by blocking the entry through the hole.

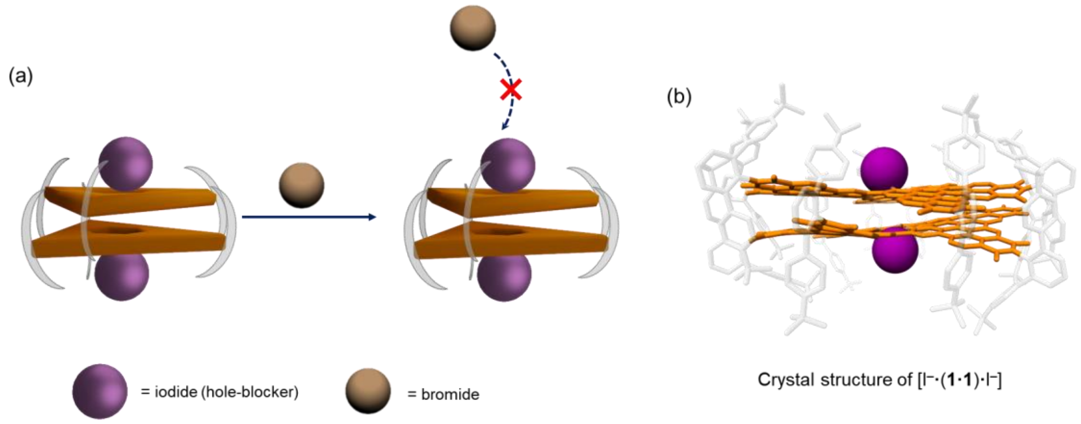

Supplementary Figure 21. (a) Schematic representation of blocking the hole using iodide. Once the hole is blocked by the iodide, the apparent rate of exchange by bromide will be affected if the bromide passes through the hole. (b) X-ray crystal structure of [I^-·(**1**·**1**)·I^-] complex shows the hole blocking by iodide.

Supplementary Table 9. Experimental rate constants calculated for bromide binding from 2D EXSY measurements at different concentrations of TBAI as hole-blocker at 343 K. Decrease in rate constant ($k_{+\mathrm{Br}}$) on increasing the concentration of blocker indicates the halide entry through the hole.

c(TBAI)	$k_{+\mathrm{Br}}$ ($M^{-1}s^{-1}$)
0	95.5
7.8×10^{-4} M	77.8
7.8×10^{-3} M	46.6

Experimental conditions: 600 MHz, 1:1 Tol-*d*8:MeCN-*d*3, *c*(**1**) = 1.5×10^{-4} M, *c*(TBABr) = 6×10^{-4} M, τ_m = 750 ms, 343 K. Diagonal and cross peak intensities obtained from 2D EXSY measurements were evaluated using the EXSYCALC software to obtain magnetization rate constant that was then converted to chemical rate constant $k_{+\mathrm{Br}}$ ($k_{+\mathrm{Br}} = k^M_{+\mathrm{Br}}/c(\mathrm{TBABr})$, where $k^M_{+\mathrm{Br}}$ is the forward magnetization rate constant).

Single crystal of [I^-· (**1**· **1**)· I^-] was obtained by mixing **1** and tetraphenylphosphonium iodide (TPPI) in 1:1 Tol:MeCN (*c*(**1**) ≈ 3 ×10^{-4} M, *c*(TPPI) ≈ 3 ×10^{-3} M, 293 K) and slow diffusion of MeCN over a week. In the crystal structure of [I^-· (**1**· **1**)· I^-], the two holes are blocked by partially occupied iodide (19% and 14% for the two sides). There was another iodide ion found at the periphery of the dimer (Supplementary Figure 3) with an occupancy of 7%. We note here that this peripheral iodide unlikely influences the hypothetical event of the lateral insertion of halide because this bay position is more densely packed than the two neighbouring bays that are likely the more favoured positions for halide entry if it should happen.

2. Structural rearrangement of large assemblies involving multiple dimer complexes (Supplementary Figure 17b2)

We considered the structural rearrangement of large assemblies without the dissociation of dimers to monomers as a possible mechanism that lead to the binding of halide. If the binding is through the single benzene hole in [**1·1**], the rate of halide binding ($v_{+\mathrm{Br}} = k_{+\mathrm{Br}} \times c([\mathbf{1 \cdot 1}]) \times c(\mathrm{X}^{-})$) will be directly proportional to the concentration of [**1·1**] (at a constant halide concentration). By contrast, if the binding of halide involves a higher-order assembly that may rearrange to bind the halide, the rate of halide binding would exhibit an order greater than two (due to the involvement of multiple dimer species). Such higher-order rate should show a significant deviation from linearity upon changing the concentration of [**1·1**]. Thus, we performed 2D EXSY experiments at different concentrations of [**1·1**] at a constant bromide concentration (Supplementary Table 10, Supplementary Figure 22). We found a linear correlation of forward rate ($v_{+\mathrm{Br}}$) against the concentration of [**1·1**], supporting the bromide permeation through the benzene hole and rejecting the possibility of higher-order kinetics involved in the binding of bromide to [**1·1**] such as the mechanism shown in Supplementary Figure 17b2 (Supplementary Figure 22).

Supplementary Table 10. Experimental rate constants and forward rate ($v_{+\mathrm{Br}}$) calculated for bromide binding to [**1·1**] from 2D EXSY measurements at different concentrations of [**1·1**] at 343 K in 1:1 Tol-d_8:MeCN-d_3.

$c([\mathbf{1 \cdot 1}])$ (M)	$k_{+\mathrm{Br}}$ ($\mathrm{M^{-1}s^{-1}}$)	Forward rate ($v_{+\mathrm{Br}}$) ($\mathrm{Ms^{-1}}$)
2.2×10^{-5}	172	5.7×10^{-7}
3.4×10^{-5}	237	1.2×10^{-6}
7.1×10^{-5}	184	1.9×10^{-6}
1.4×10^{-4}	163	3.4×10^{-6}

Experimental conditions: 600 MHz, 1:1 Tol-d_8:MeCN-d_3, c(TBABr) = 1.5×10^{-4} M, τ_m = 750 ms, 343 K. Diagonal and cross peak intensities obtained from 2D EXSY measurements were evaluated using the EXSYCALC software to obtain magnetization rate constant that was then converted to chemical rate constant $k_{+\mathrm{Br}}$ ($k_{+\mathrm{Br}} = k^{M}_{+\mathrm{Br}}/c(\mathrm{TBABr})$, where $k^{M}_{+\mathrm{Br}}$ is the forward magnetization rate constant).

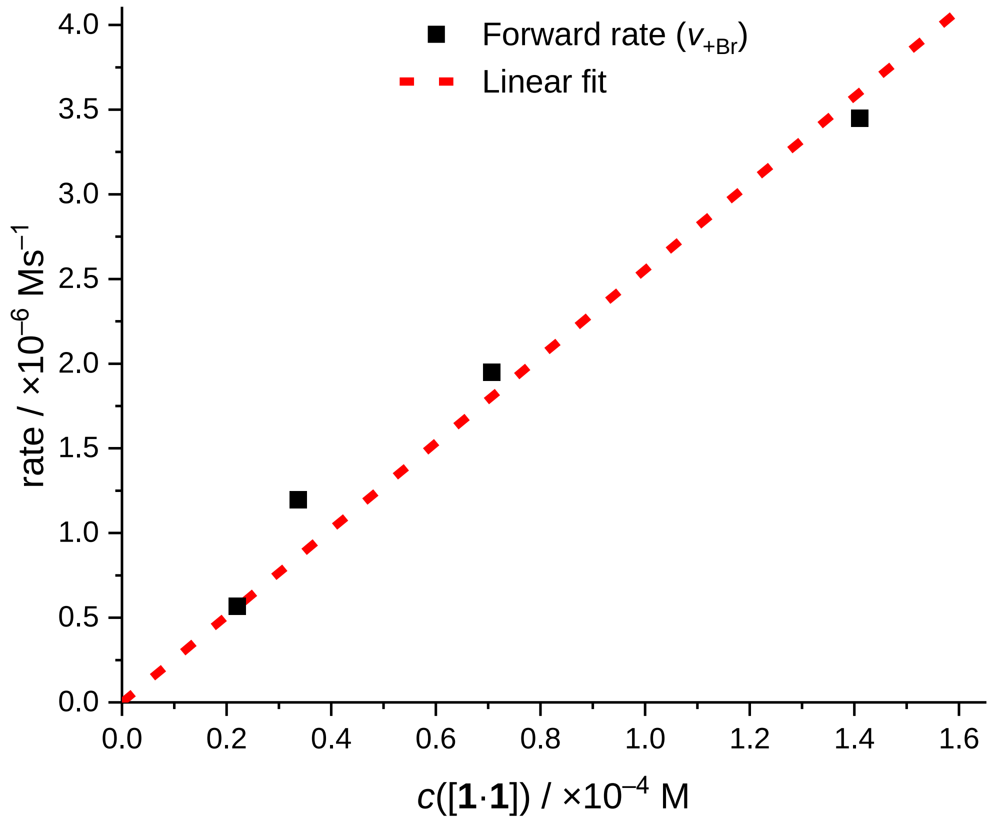

Supplementary Figure 22. Plot of bromide binding rate at different concentrations of [**1**·**1**] (1:1 Tol-d_8:MeCN-d_3, c(TBABr) = 1.5 × 10^{-4} M, 343 K). A linear increase in rate with respect to concentration of [**1**·**1**] supports the entry of bromide through the benzene hole.

9 NMR Spectroscopy

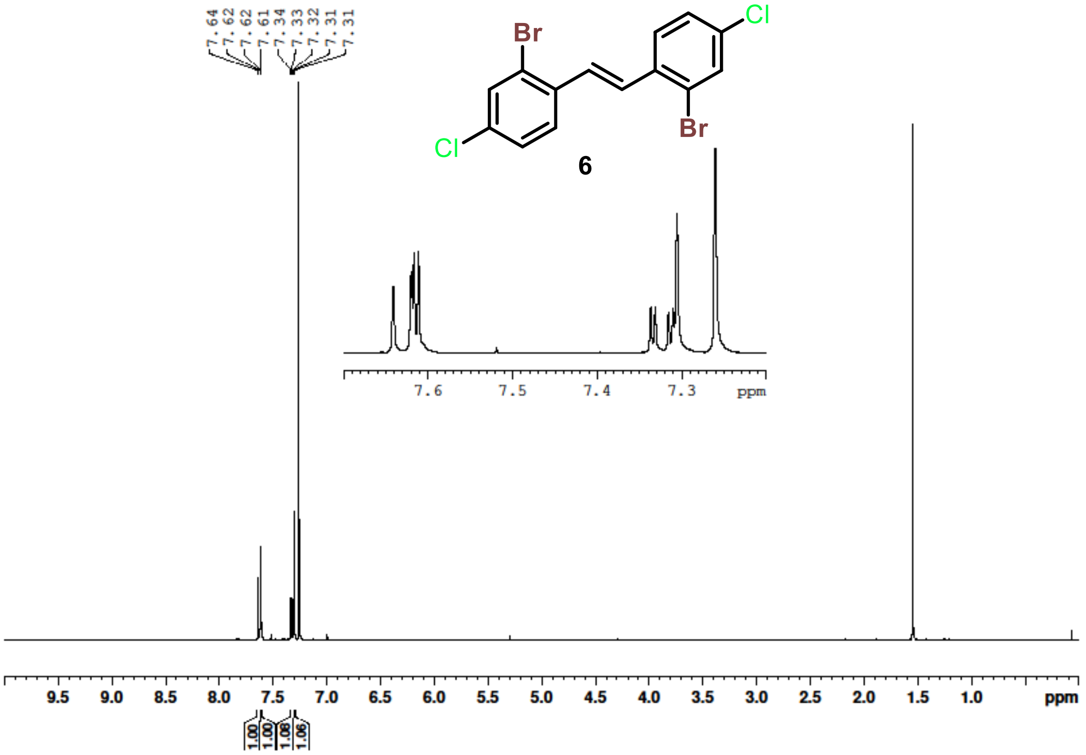

Supplementary Figure 23. ^{1}H NMR (400 MHz) of **6** in $CDCl_3$ at 295 K.

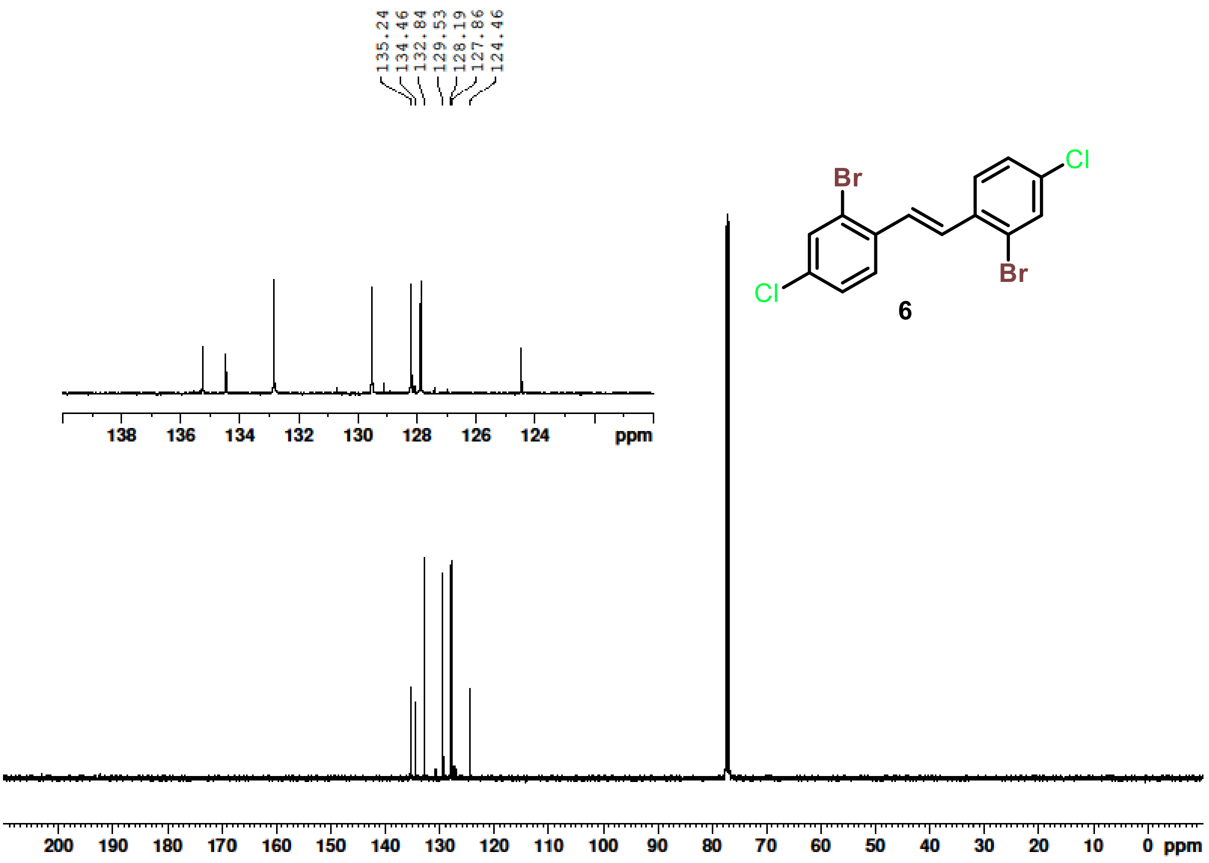

Supplementary Figure 24. ^{13}C NMR (101 MHz) of **6** in $CDCl_3$ at 295 K.

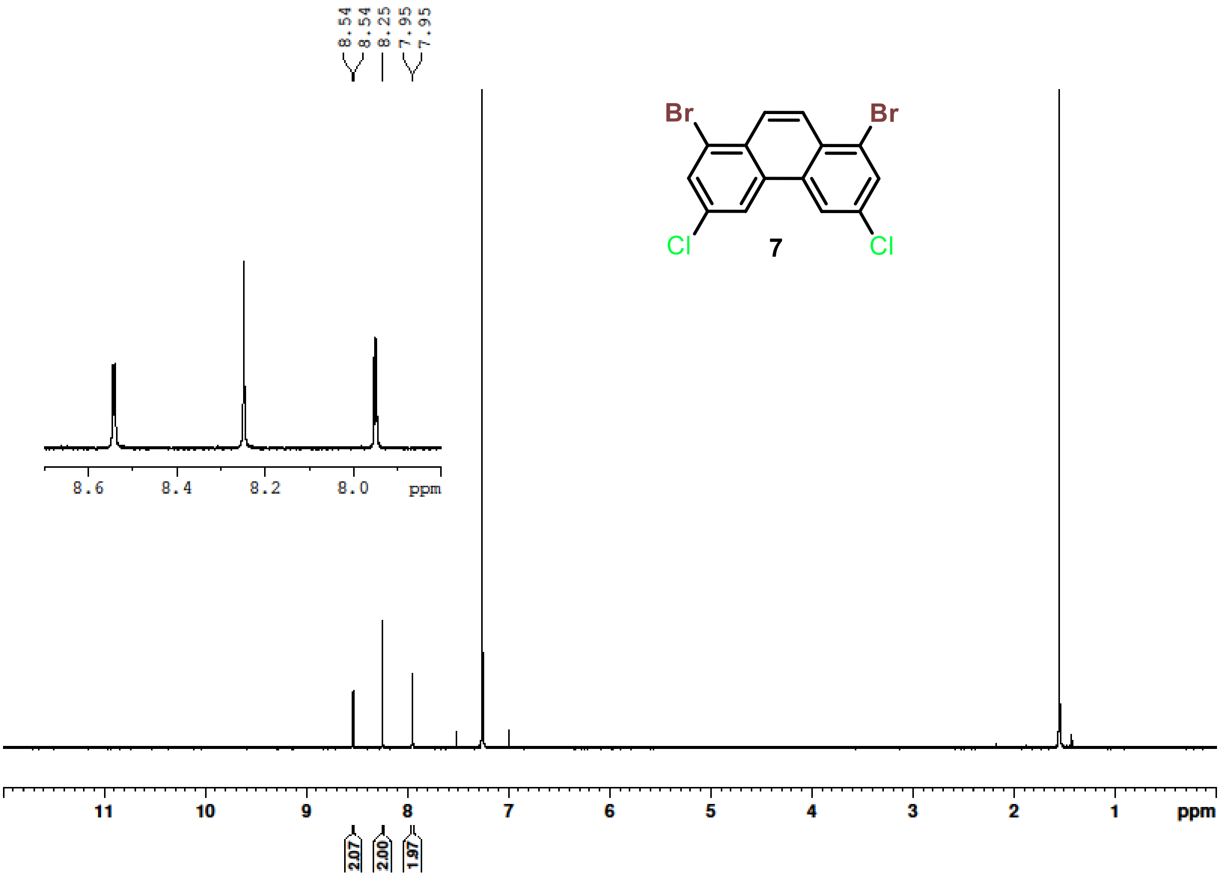

Supplementary Figure 25. ^{1}H NMR (400 MHz) of **7** in $CDCl_3$ at 295 K.

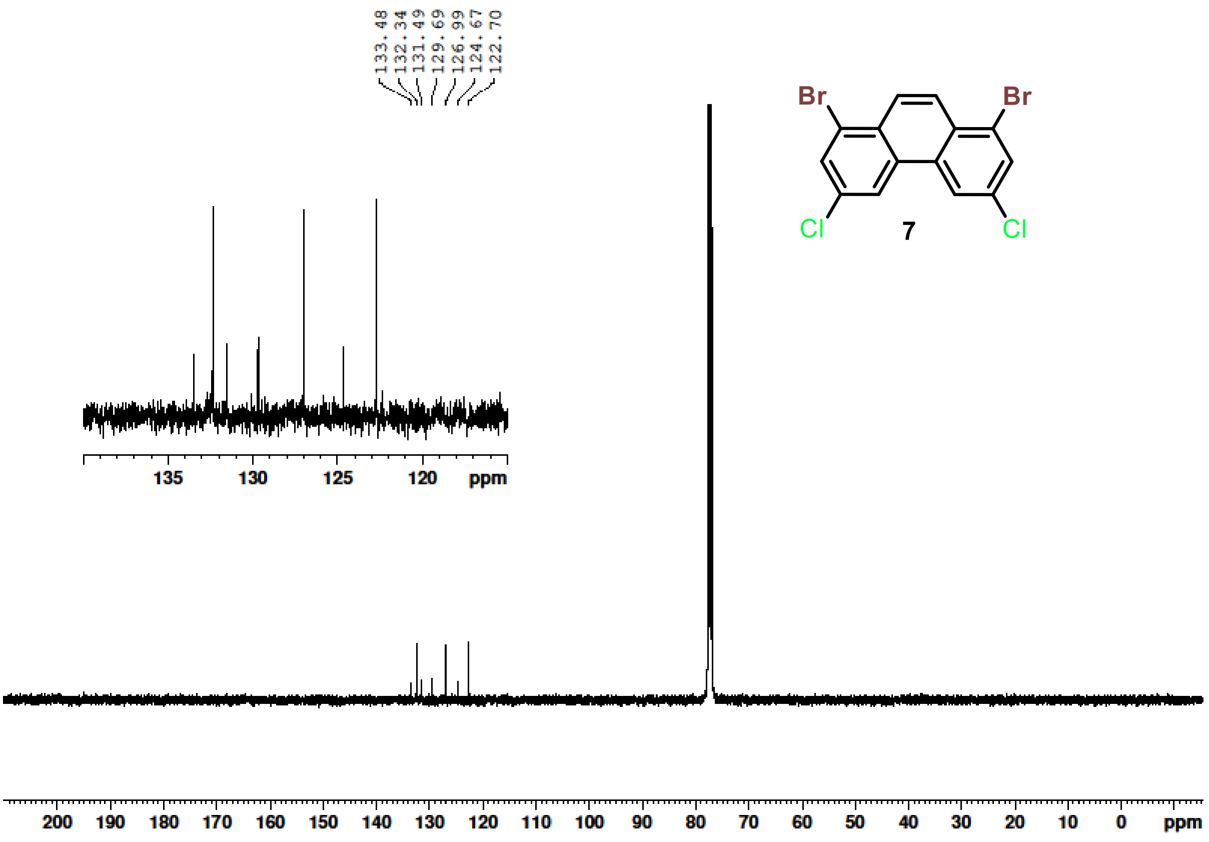

Supplementary Figure 26. ^{13}C NMR (101 MHz) of **7** in $CDCl_3$ at 295 K.

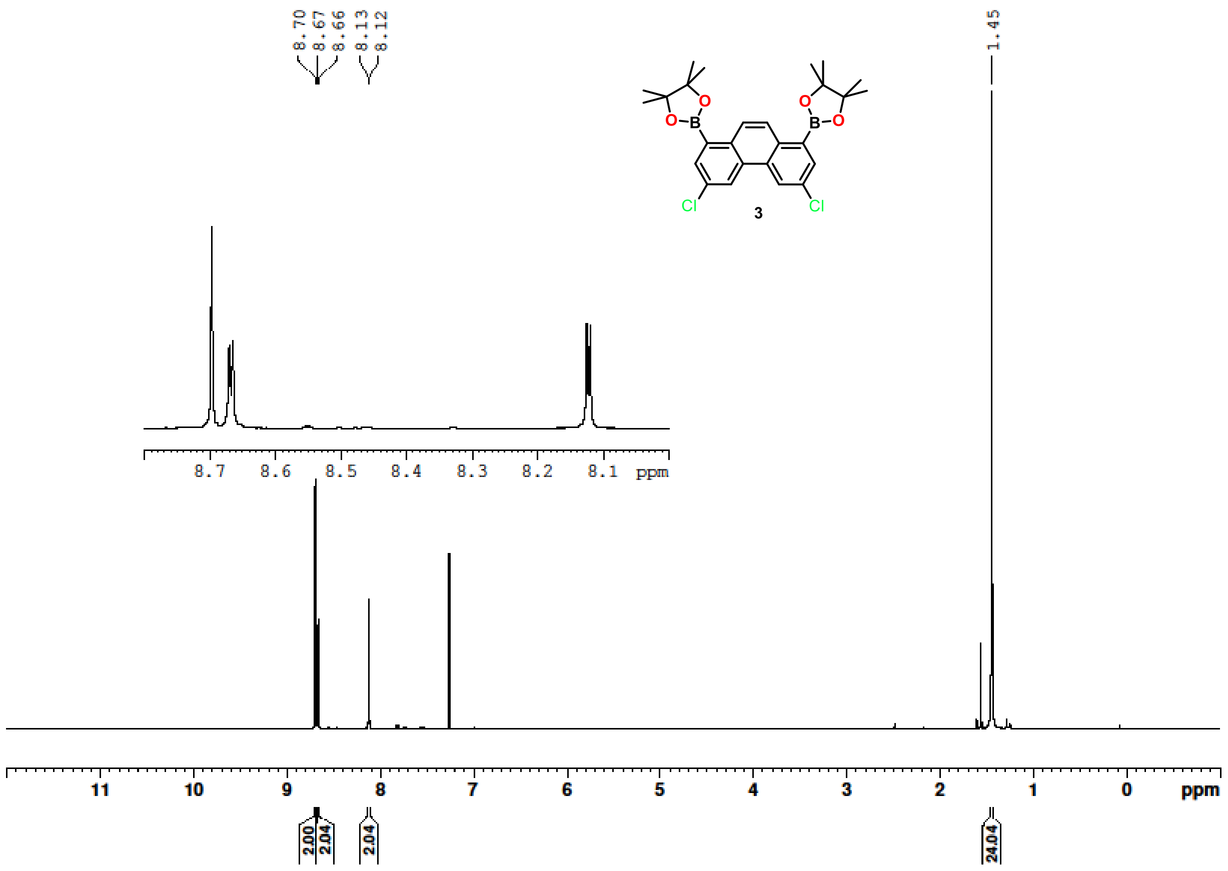

Supplementary Figure 27. ^{1}H NMR (400 MHz) of **3** in $CDCl_3$ at 295 K.

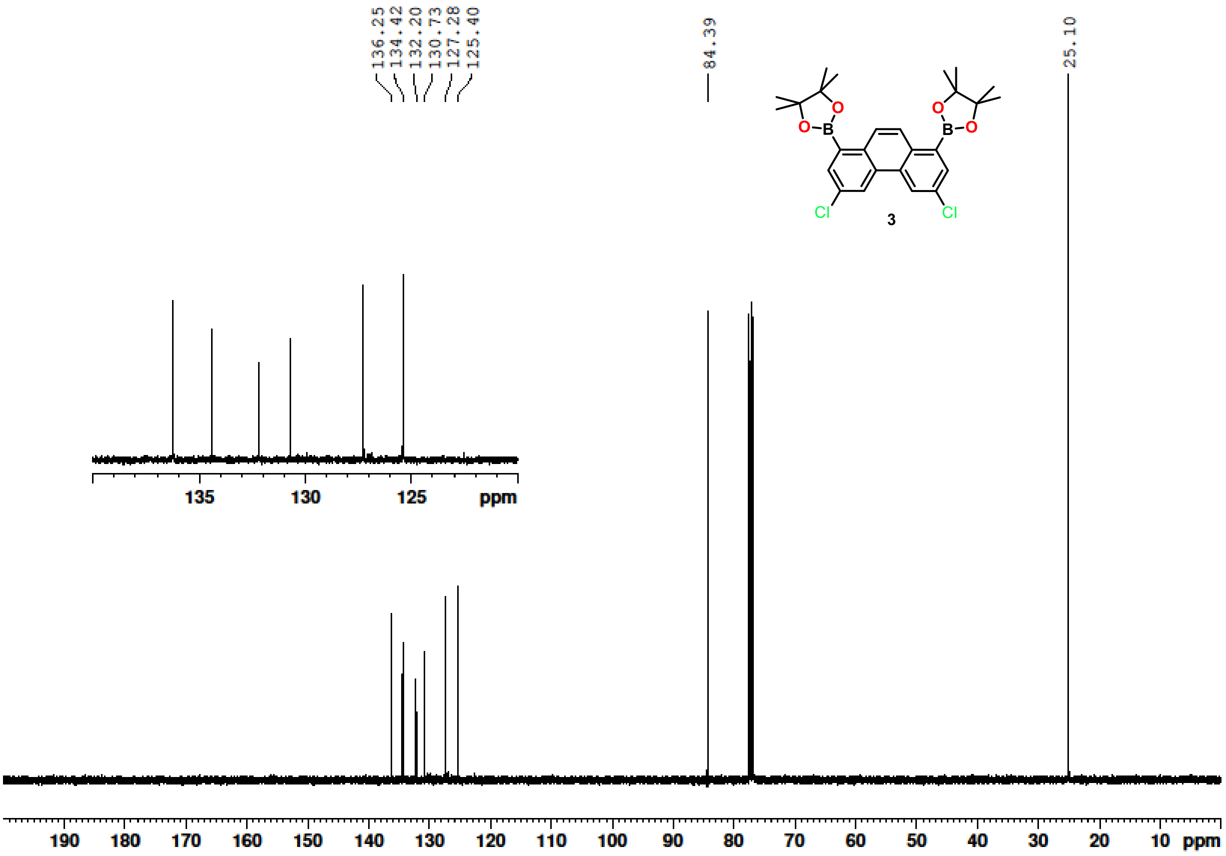

Supplementary Figure 28. ^{13}C NMR (101 MHz) of **3** in $CDCl_3$ at 295 K.

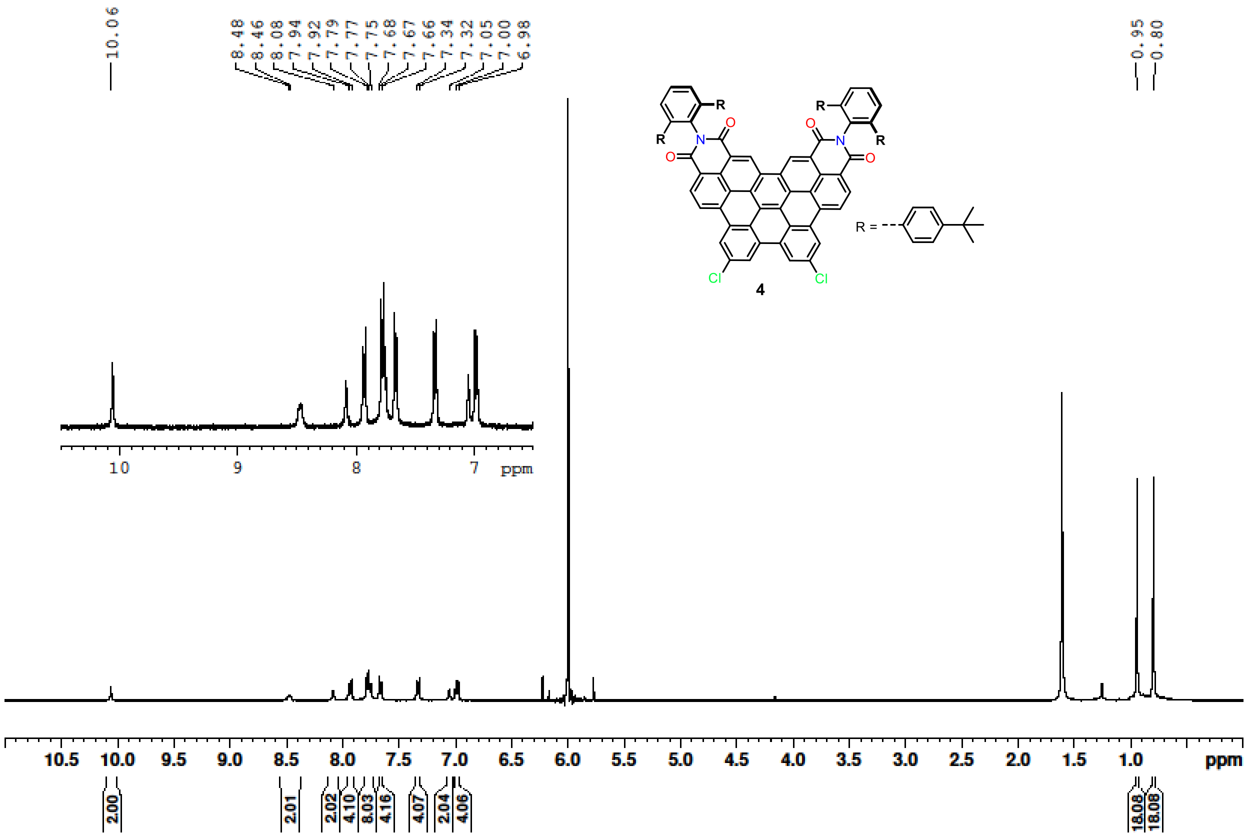

Supplementary Figure 29. ^{1}H NMR (400 MHz) of **4** in TCE-d_2 at 295 K.

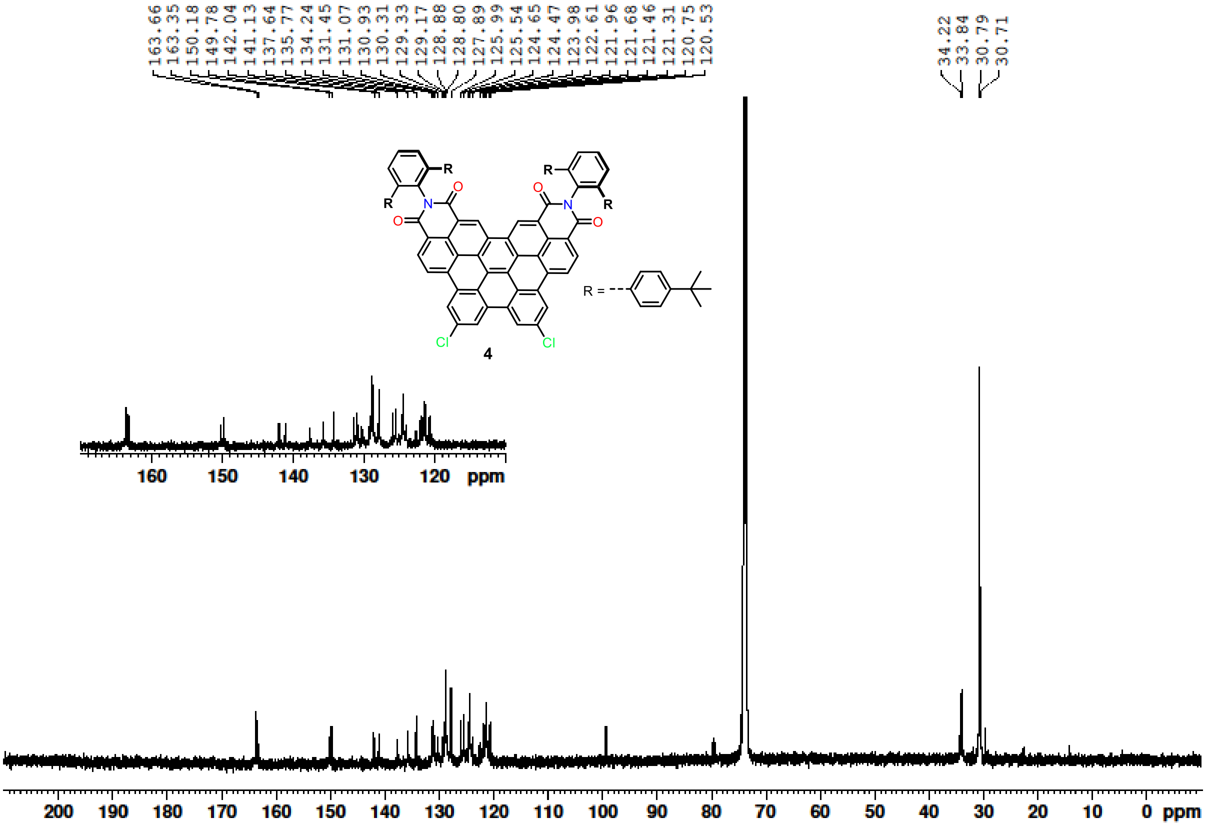

Supplementary Figure 30. ^{13}C NMR (101 MHz) of **4** in TCE-d_2 at 295 K.

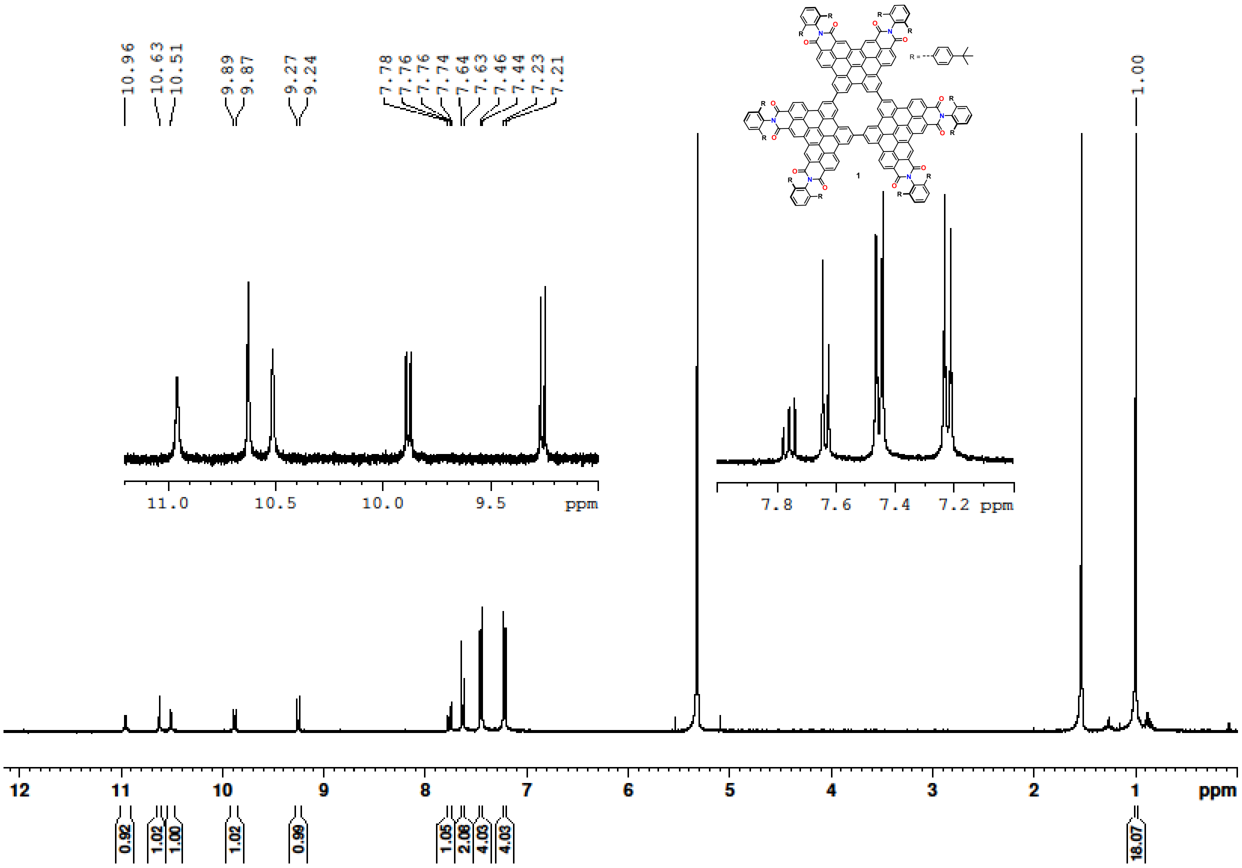

Supplementary Figure 31. ^{1}H NMR (400 MHz) of **1** in CD_2Cl_2 at 295 K.

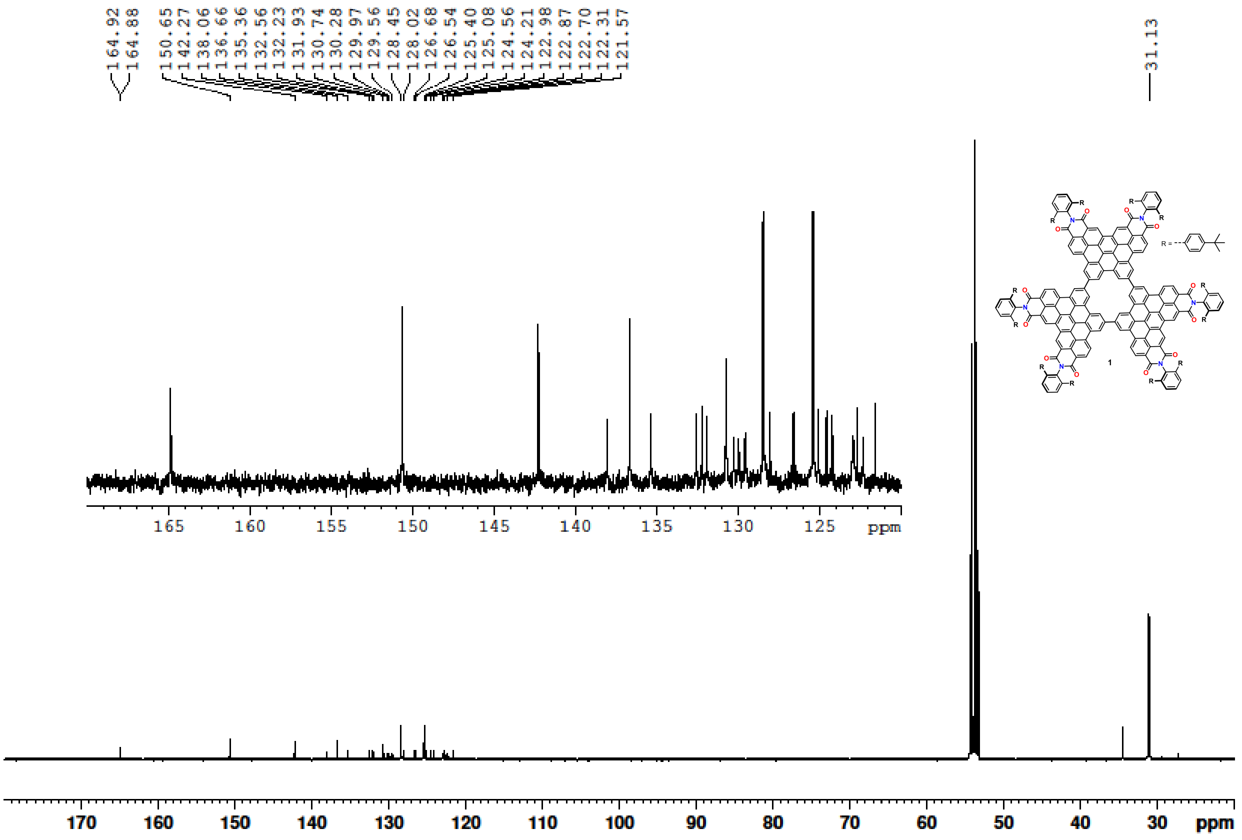

Supplementary Figure 32. ^{13}C NMR (101 MHz) of **1** in CD_2Cl_2 at 295 K.

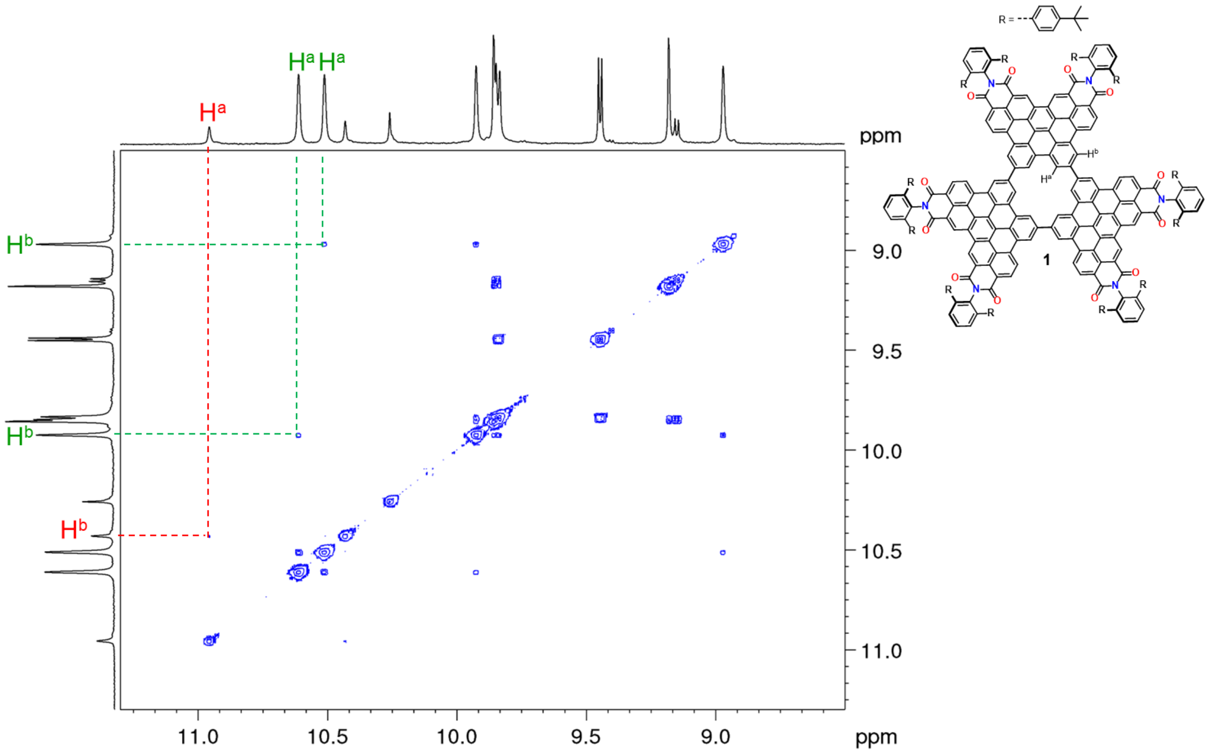

Supplementary Figure 33. ^{1}H–^{1}H COSY spectrum of **1** in 1:1 Tol-d_8:MeCN-d_3 (c(**1**) = 1.5 × 10^{-4} M, 600 MHz, 343 K) showing the coupling between H^a and H^b in monomeric **1** (in red) and dimeric [**1**·**1**] (in green).

10 Mass Spectrometry

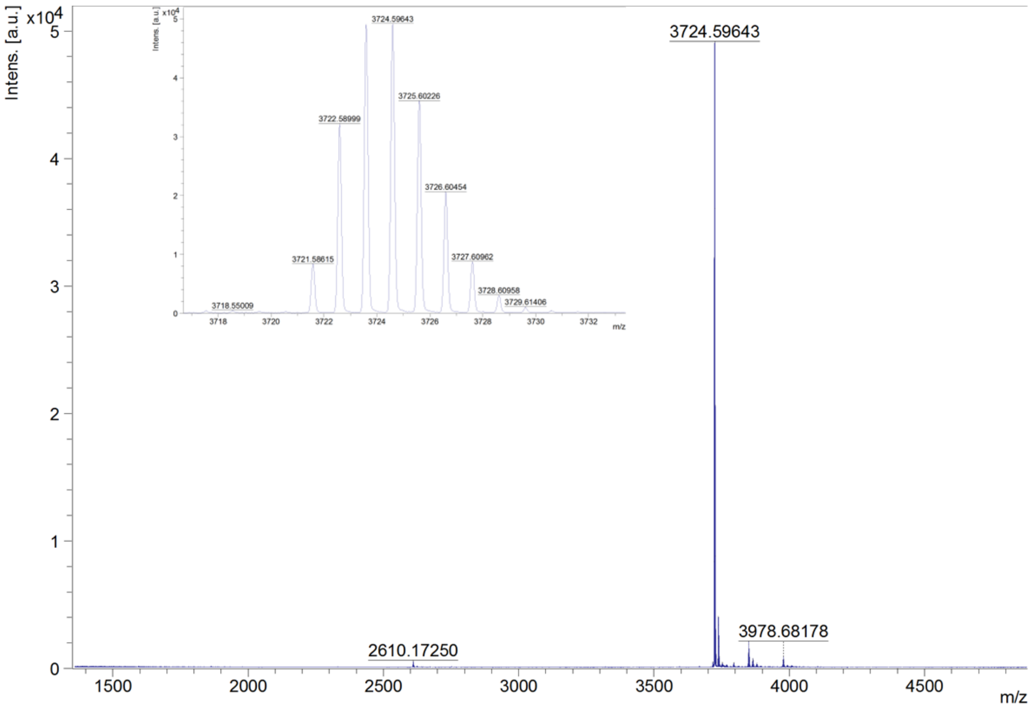

Supplementary Figure 34. Mass (MALDI-TOF, negative mode, DCTB in chloroform) spectrum of **1** in $CHCl_3$ showing the mass of monomeric **1**.

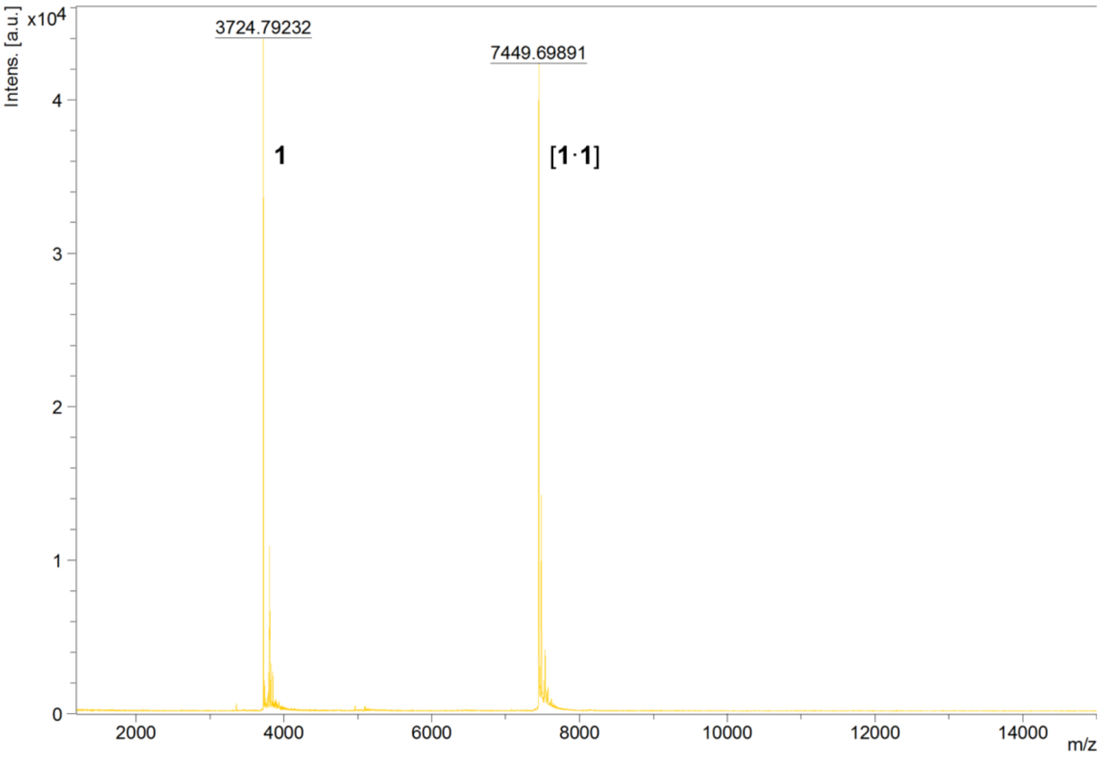

Supplementary Figure 35. Mass (MALDI-TOF, negative mode, DCTB in chloroform) spectrum of **1** in 1:1 Tol:MeCN (c(**1**) = 1 × 10^{-4} M) showing the masses of dimer ([**1**·**1**]) and monomer (**1**).

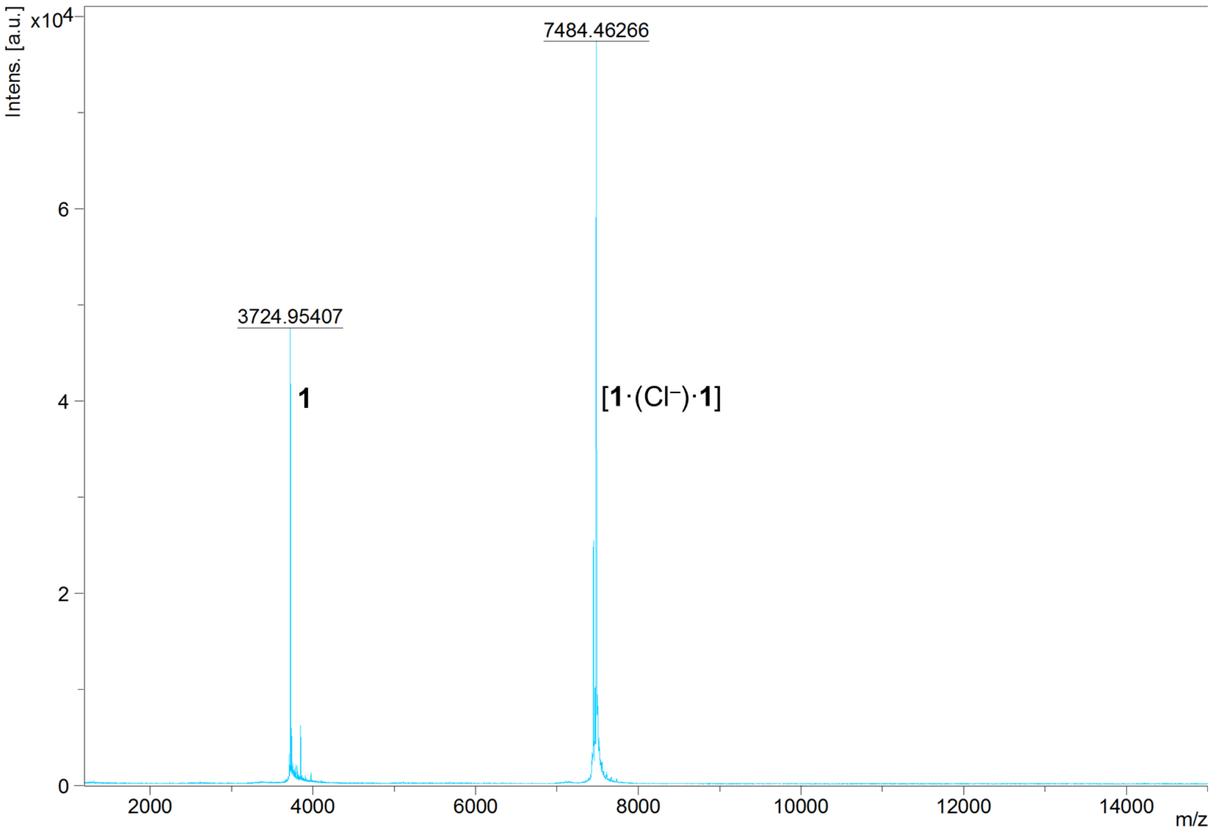

Supplementary Figure 36. Mass (MALDI-TOF, negative mode, DCTB in chloroform) spectrum of **1** and excess TBACl in 1:1 Tol:MeCN (c(**1**) = 1 × 10^{-4} M) showing the masses of dimer-chloride complex ([**1**·(Cl^{-})·**1**]) and monomer (**1**).

11 References

1. Zalesskiy, S. S. & Ananikov, V. P. $Pd_2(dba)_3$ as a Precursor of Soluble Metal Complexes and Nanoparticles: Determination of Palladium Active Species for Catalysis and Synthesis. *Organometallics* **31**, 2302–2309 (2012).
2. Mahl, M., Niyas, M. A., Shoyama, K. & Würthner, F. Multilayer stacks of polycyclic aromatic hydrocarbons. *Nat. Chem.* **14**, 457–462 (2022).
3. Sheldrick, G. M. SHELXT – Integrated space-group and crystal-structure determination. *Acta Crystallogr. A* **1**, 3–8 (2015).
4. Sheldrick, G. M. A short history of SHELX. *Acta Crystallogr. A* **1**, 112–122 (2008).
5. Guzei, I. A. An idealized molecular geometry library for refinement of poorly behaved molecular fragments with constraints. *J. Appl. Crystallogr.* **47**, 806–809 (2014).
6. Spek, A. L. Single-crystal structure validation with the program PLATON. *J. Appl. Crystallogr.* **1**, 7–13 (2003).
7. Evans, P. R. & Murshudov, G. N. How good are my data and what is the resolution? *Acta Crystallogr. D* **69**, 1204–1214 (2013).
8. Wagner, R. & Berger, S. Gradient-Selected NOESY—A Fourfold Reduction of the Measurement Time for the NOESY Experiment. *J. Magn. Reson., Ser. A* **123**, 119–121 (1996).
9. Perrin, C. L. & Dwyer, T. J. Application of two-dimensional NMR to kinetics of chemical exchange. *Chem. Rev.* **90**, 935–967 (1990).
10. Pavlović, R. Z., Lalisse, R. F., Hansen, A. L., Waudby, C. A., Lei, Z., Güney, M., Wang, X., Hadad, C. M. & Badjić, J. D. From Selection to Instruction and Back: Competing Conformational Selection and Induced Fit Pathways in Abiotic Hosts. *Angew. Chem. Int. Ed.* **60**, 19942–19948 (2021).
11. Zolnai, Z., Juranić, N., Vikić-Topić, D. & Macura, S. Quantitative Determination of Magnetization Exchange Rate Constants from a Series of Two-Dimensional Exchange NMR Spectra. *J. Chem. Inf. Comput. Sci.* **40**, 611–621 (2000).
12. Lu, J., Ma, D., Hu, J., Tang, W. & Zhu, D. Nuclear magnetic resonance spectroscopic studies of pyridine methyl derivatives binding to cytochrome c. *J. Chem. Soc., Dalton Trans.* 2267–2273 (1998).
13. Miklitz, M. & Jelfs, K. E. pywindow: Automated Structural Analysis of Molecular Pores. *J. Chem. Inf. Model.* **12**, 2387–2391 (2018).
14. Maglic, J. B. & Lavendomme, R. MoloVol: an easy-to-use program for analyzing cavities, volumes and surface areas of chemical structures. *J. Appl. Crystallogr.* **55**, 1033–1044 (2022).
15. Shao, Y. et al. Advances in molecular quantum chemistry contained in the Q-Chem 4 program package. *Mol. Phys.* **113**, 184–215 (2015).
16. Neese, F., Wennmohs, F., Becker, U. & Riplinger, C. The ORCA quantum chemistry program package. *J. Chem. Phys.* **152**, 224108 (2020).
17. Bannwarth, C., Ehlert, S. & Grimme, S. GFN2-xTB-An Accurate and Broadly Parametrized Self-Consistent Tight-Binding Quantum Chemical Method with Multipole Electrostatics and Density-Dependent Dispersion Contributions. *J. Chem. Theory Comput.* **3**, 1652–1671 (2019).
